# Sequelae of child maltreatment: Umbrella synthesis of 148 meta‐analyses on the mental health correlates

**DOI:** 10.1002/jcv2.70081

**Published:** 2026-01-15

**Authors:** Barry Coughlan, Robbie Duschinsky, Marian J. Bakermans‐Kranenburg, Lianne Bakkum, Guy C. M. Skinner, Alfred Markham, Helen Beckwith, Marinus H. van Ijzendoorn

**Affiliations:** ^1^ Department of Public Health and Primary Care University of Cambridge Cambridge UK; ^2^ Department of Business and Social Sciences National College of Ireland Dublin Ireland; ^3^ ISPA ‐ University Institute of Psychological Social and Life Sciences Lisbon Portugal; ^4^ Centre for Attachment Research The New School for Social Research New York New York USA; ^5^ Facultad de Psicología y Humanidades Universidad San Sebastián Valdivia Chile; ^6^ Department of Educational and Family Studies Vrije Universiteit Amsterdam Amsterdam Netherlands; ^7^ Tavistock and Portman NHS Foundation Trust London UK; ^8^ School of Psychology University of Southampton Southampton UK; ^9^ Department of Psychiatry University of Oxford Oxford UK; ^10^ Research Department of Clinical Faculty of Brain Sciences Education and Health Psychology UCL University of London London UK

**Keywords:** abuse, child development, child harm, meta‐analysis review, neglect, psychiatric disorder

## Abstract

**Background:**

Numerous meta‐analyses have established associations between child maltreatment (CM) and mental health difficulties (MH). However, variation exists between meta‐analyses regarding the magnitude of these predictions.

**Methods:**

A systematic, quantitative umbrella synthesis (i.e., meta‐analysis of meta‐analyses) was undertaken to describe the associations between various types of CM and MH. Meta‐analyses were included if they examined CM, including but not limited to retrospective reports in adulthood, and MH at any point. Included forms of CM were: physical abuse, emotional abuse, sexual abuse, neglect, and exposure to intimate partner violence. MH outcomes were: externalising problems, internalising problems, thought problems, suicidal distress, substance misuse, and other psychological difficulties. Searches were run in January 2024. Random effects models were created in R version 4.2.0.

**Results:**

We analysed and combined effect sizes from 148 quantitative meta‐analyses, including 668 effect sizes and over 9.5 million data points. CM was associated with all MH outcomes: (1) externalising problems (*r* = 0.21; 95% CI = 0.18–0.24; *k* = 32), (2) internalising problems (*r* = 0.22; 95% CI = 0.20–0.24; *k* = 46), (3) thought problems (*r* = 0.24; 95% CI = 0.21–0.27; *k* = 38), (4) suicidal distress (*r* = 0.23; 95% CI 0.18–0.28; *k* = 19), (5) substance misuse (*r* = 0.19; 95% CI = 0.13–0.26; *k* = 13), (6) other psychological difficulties (*r* = 0.24; 95% CI = 0.20–0.28; *k* = 50). Associations tend to be of similar magnitude for different forms of CM.

**Conclusion:**

CM is robustly associated with MH. A parsimonious explanation for these findings would be a common mechanism(s) or a general psychopathology factor conferring high‐risk for different mental health difficulties following CM. The results possibly question the conventional wisdom that suggests some forms of maltreatment are intrinsically more harmful to mental health than others. However, further work is required to understand how potentially confounding factors (e.g., age, measurement of CM) influence these associations.

## Key points

**What's known**

- Child maltreatment (CM) is regarded as an important risk factor for various adverse outcomes, especially mental health issues.

**What's new**

- This umbrella synthesis found strong, often equivalent, associations between CM and all examined forms of mental ill‐health.
- Different types of CM appear to have comparably negative effects on mental health at the population level.

**What's relevant**

- If replicated, these findings may make us reconsider conventional wisdom that some forms of CM are less harmful than others.

## Graphical abstract

Our umbrella synthesis found strong, often equivalent, associations between child maltreatment and all examined mental health difficulties. Different types of maltreatment appear to have comparably negative effects on mental health. If replicated, these findings may cause us to reconsider conventional wisdom that suggests some forms of CM are less harmful to mental health than others.

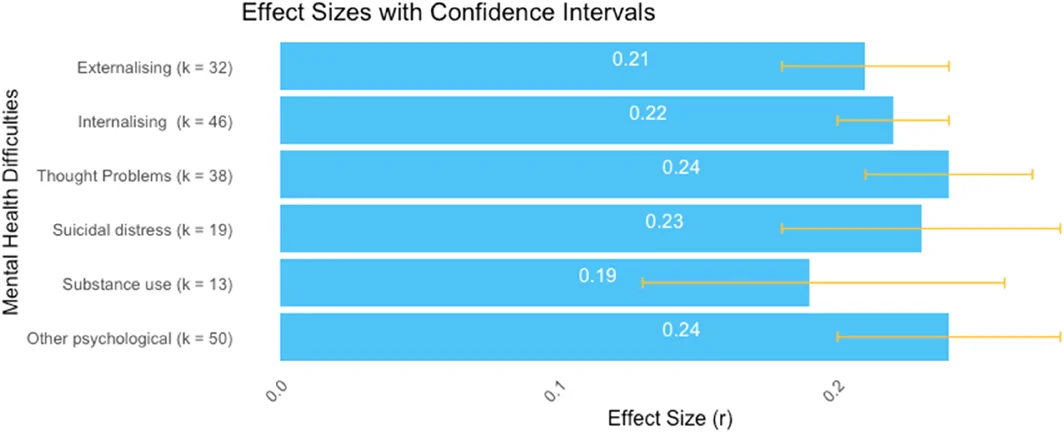

## INTRODUCTION

Child maltreatment (CM), defined as behaviours and situations that are likely to result in ‘harm to the child's health, survival, development or dignity in the context of a relationship of responsibility, trust or power’ (World Health Organisation, 2020) impacts millions of people around the world (Stoltenborgh et al., 2015). Numerous meta‐analytic reviews have documented associations between CM in its various forms and mental health outcomes such as externalising and internalising problems (Hughes et al., 2017; Petruccelli et al., 2019). It is widely accepted that meta‐analytic findings are regarded as a potent form of empirical evidence. However, there is often material variation in the magnitude of effect sizes reported in meta‐analyses on CM (e.g., Li et al., 2022; Tang et al., 2019), creating potential challenges for policy‐makers, researchers, and clinicians alike.

For instance, variations in evidence can make it challenging to determine the overall magnitude of associations at a population level, thus potentially hindering effective evidence‐based policy‐making. For researchers, clarity around the average effect is useful in terms of providing a robust empirical referent for hypothesis‐driven work on CM and mental health, including examining mechanisms. Additionally, variation between effect sizes reported in meta‐analyses creates a practical challenge for researchers conducting a priori power analyses, for which a previous effect is required and is often gleaned from existing meta‐analytic studies. From a clinical perspective, knowledge of the magnitude of these associations can inform treatment decisions and assessment practices, for example, providing empirically grounded prior probabilities in clinical reasoning.

Umbrella synthesis is one useful method for managing and interpreting variation between meta‐analyses by providing an overview of all meta‐analytic effect sizes on a specific topic (Ioannidis, 2009). According to Fusar‐Poli and Radua (2018) umbrella syntheses present one of the highest levels of evidence in biomedical sciences, providing important insights in putative associations at a population level. Such insights provide a firm foundation for translation to policy or (clinical) practice, although they may not be directly applicable at the individual level (Van IJzendoorn & Bakermans‐Kranenburg, 2024).

To date, several umbrella syntheses have examined the association between aspects of CM and aspects of mental health. These efforts have mostly adopted a ‘predictor’ or ‘outcome approach’, focusing on a specific form of maltreatment or a specific mental health outcome (Hailes et al., 2019; Hutchens & Kearney, 2020; Sahle et al., 2021). Recently, Kim and Royle (2025) conducted a large umbrella synthesis (*k* = 99) on the associations between CM and various health outcomes, including mental health broadly defined, finding that CM was a robust predictor of mental health difficulties. While these studies have produced valuable information about the sequelae of CM, it remains unclear: a) whether CM is more strongly associated with some mental health sequelae (e.g., internalising problems) than others (e.g., externalising problems), b) whether certain forms of CM (e.g., physical abuse [PA]) are more strongly associated with specific mental health sequelae than others (e.g., emotional abuse [EA]). For example, until rather recently and in sharp contrast to sexual or PA, emotional neglect was considered a mild type of insensitive parenting without many negative consequences for child development, and its study was under‐developed (Stoltenborgh et al., 2012).

These two approaches, with either a focus on specific maltreatment forms or specific outcomes, or using mental health as an overall outcome, leave a research gap with potential theoretical and empirical consequences for hypotheses regarding multifinality and equifinality: the ideas that a given developmental experience can contribute to an array of outcomes (i.e., multifinality) and also that different developmental experiences can contribute to the same outcome (i.e., equifinality; Cicchetti & Rogosch, 1996). For instance, the question of whether certain forms of maltreatment (e.g., PA or EA) are more robust predictors of certain mental health difficulties (e.g., internalising or externalising problems) might support further hypothesis‐driven research on mechanisms (e.g., negative affectivity) by hinting at distinct developmental pathways and identifying transdiagnostic risk factors. It is this research gap that we address with our current umbrella synthesis.

Thus, the current umbrella synthesis aims to quantify and compare the associations between CM in its various forms and a range of mental health difficulties in what is, to our knowledge, the largest umbrella synthesis undertaken on the topic to date. In doing so, an objective of this synthesis is to provide robust empirical insights about these associations that may be useful for policy‐makers, researchers, and clinicians, and shed light on the issues of multifinality and equifinality.

## METHODS

### Overview

Following PRISMA guidelines for systematic and meta‐analytic reviews (Moher et al., 2009) and best practice guidelines for umbrella syntheses (Aromataris et al., 2015; Fusar‐Poli & Radua, 2018), we carried out a quantitative synthesis of 148 meta‐analyses that included effect sizes on the association between CM and various aspects of mental health. Overall, 668 effect sizes were extracted. A review protocol was submitted to PROSPERO on 15 July 2021 and registered on 10 August 2021 (CRD: 42021266037). This protocol outlined a series of umbrella syntheses investigating the association between CM and four types of outcome: mental health and related difficulties, social/educational, physical and neurobiological sequelae. The current paper focuses on mental health and related difficulties. An extended preprint of this paper was uploaded to PsyArXiv (see Coughlan et al., 2022).

### Search strategy

The search included three components:

1) Population (i.e., children)
2) Exposure (i.e., maltreatment and adversity)
3) Study design (i.e., quantitative review)

The full list of search terms and counts can be found in Supporting Information S1: Appendix S1. Nine electronic databases were systematically searched on July 19, 2021: Applied Social Science Index and Abstracts (via ProQuest), Dissertations and Theses (via ProQuest), Cochrane Library, Embase (via Ovid), Medline (via Ovid), PsycINFO (via Ebsco host), PubMed, Scopus, Web of Knowledge (all collections). We did not impose geographical parameters on the reviews. Reviews in English, German or Dutch were considered for inclusion. The search was then rerun in May 2023 for the period (2021–2023) and then again in January 2024 (2023–2024).

In the initial search, we identified *k* = 7300 records. 4908 duplicates were identified. We imported the remaining *k* = 2392 records to Rayyan, identifying an additional *k* = 219 duplicate citations. After removing these duplicates, a total of *k* = 2173 titles and abstracts were screened. Of these, *k* = 447 were brought to full‐text review, *k* = 125 of which were deemed to meet our inclusion criteria.

In the search conducted in May 2023 we identified *k* = 1441 citations, including *k* = 76 duplicates which were removed. Title and abstract screening was completed on the remaining *k* = 1365 citations, resulting in *k* = 97 citations being brought to full text review. Of these *k* = 44 were included in the current umbrella synthesis. A final search was conducted in January 2024. This search identified *k* = 1033 citations, *k* = 828 of which were identified as duplicates or had already been identified in a previous round of searching. Thus, *k* = 205 citations were considered for title and abstract screening. In this final phase of the search, an additional *k* = 14 meta‐analyses were included.

Eight meta‐analyses were excluded because they reported proportions or percentages that could not be converted to r due to a lack of information about both number of participants who experienced CM and the number who experienced the outcome. Nine meta‐analyses were excluded because they exclusively examined prenatal substance misuse, and 17 were excluded because they examined a non‐mental health outcome (e.g., victimisation, executive functioning).

### Selection criteria

#### Child maltreatment

Meta‐analyses were included if they provided either a prospective or retrospective account of CM during childhood or adolescence, including informant reports by Child Protective Services and retrospective accounts in adulthood. In this umbrella synthesis, we have drawn on the World Health Organisation's (2020) description of CM in the family context, including four types of harm: PA, EA, sexual abuse, and neglect. For this analysis, physical and emotional neglect were considered as ‘neglect’. We also included exposure to intimate partner violence (IPV, e.g., Carpenter & Stacks, 2009). Initially, we also included meta‐analyses on prenatal substance misuse but following reviewer suggestions we excluded prenatal substance misuse meta‐analyses from the current umbrella synthesis.

A further consideration regarding the measurement of CM is also relevant here. Since the publication of Felitti et al. (1998) landmark study, the adverse childhood experiences (ACEs) questionnaire has become popular to measure CM. Therefore, we have included studies that used the ACEs questionnaire. However, ACEs are presented as an aggregate score, hampering the disentanglement of the relative contribution of maltreatment versus other adversities (e.g., parental mental health problems). Findings will be interpreted in light of this methodological consideration and subgroup analyses have been conducted examining specific types of CM, excluding the broader category of CM and ACEs.

#### Mental health

Our inclusion criteria permitted any type of mental health difficulty. We distinguished six higher‐order categories of mental health difficulties: externalising problems, internalising problems, thought problems, suicidal distress, substance misuse, and other psychological difficulties (see Table 1). These categories were derived from various sources, including the developmental literature (Caspi et al., 2014) psychiatric classification systems (World Health Organisation, 2019), and theory (e.g., attachment theory). We did not exclude any studies based on the timing or type of mental health assessment.

**TABLE 1:** Grouping of mental health difficulties.

Outcome class	Description
Externalising problems	This category was derived from Caspi's et al. (2014) description of externalising problems and included the following outcomes: Conduct disorder, oppositional defiant disorder, externalising behavioural problems, aggressive behaviours, unspecific behavioural problems, delinquency, antisocial behaviour, violence in clinical populations, intimate partner violence perpetration, anger, and committing sexual offences.
Internalising problems	This category was derived from Caspi et al. (2014) description of internalising problems and included the following outcomes: Depression, anxiety, internalising behavioural problems (e.g. as defined by behavioural measures), postnatal depression, depression and anxiety in clinical populations (e.g. people with bipolar disorder), negative cognitive styles.
Thought problems	This category was derived from Caspi et al. (2014) description of thought problems and included the following outcomes: Dissociation, bipolar disorder, personality disorders, post‐traumatic stress disorder, schizophrenia, psychosis and symptom severity, eating disorders, and positive and negative symptoms associated with psychosis (e.g. hallucinations).
Suicidal distress	This category was derived from the representation of suicide and self‐harm in ICD‐11 and included the following outcomes: Suicide attempts, suicidal ideation, self‐harm, and suicide.
Substance misuse	Unlike Caspi et al. (2014), which conceptualised substance misuse as an externalising problem, we have included substance misuse as a separate category. This category included the following outcomes: Harmful drug and alcohol use and drug and alcohol disorders.
Other psychological difficulties	This category included various aspects of psychological functioning which are not exclusively linked to any of the aforementioned categories. This category included the following outcomes: Attachment insecurity, psychological dependence, emotional dysregulation, avoidance, emotional suppression, negative affect, daily living problems, socialisation, communication problems, moral reasoning, mistrust, abandonment, social isolation, enmeshment, self‐sacrifice, emotional inhibition, unrelenting standards, and entitlement.

#### Study design

Reviews were included if they reported a meta‐analysis and presented effect sizes (e.g., *r*, OR, Cohen's *d*) to quantify the association between CM and mental health outcomes.

**FIGURE 1:**
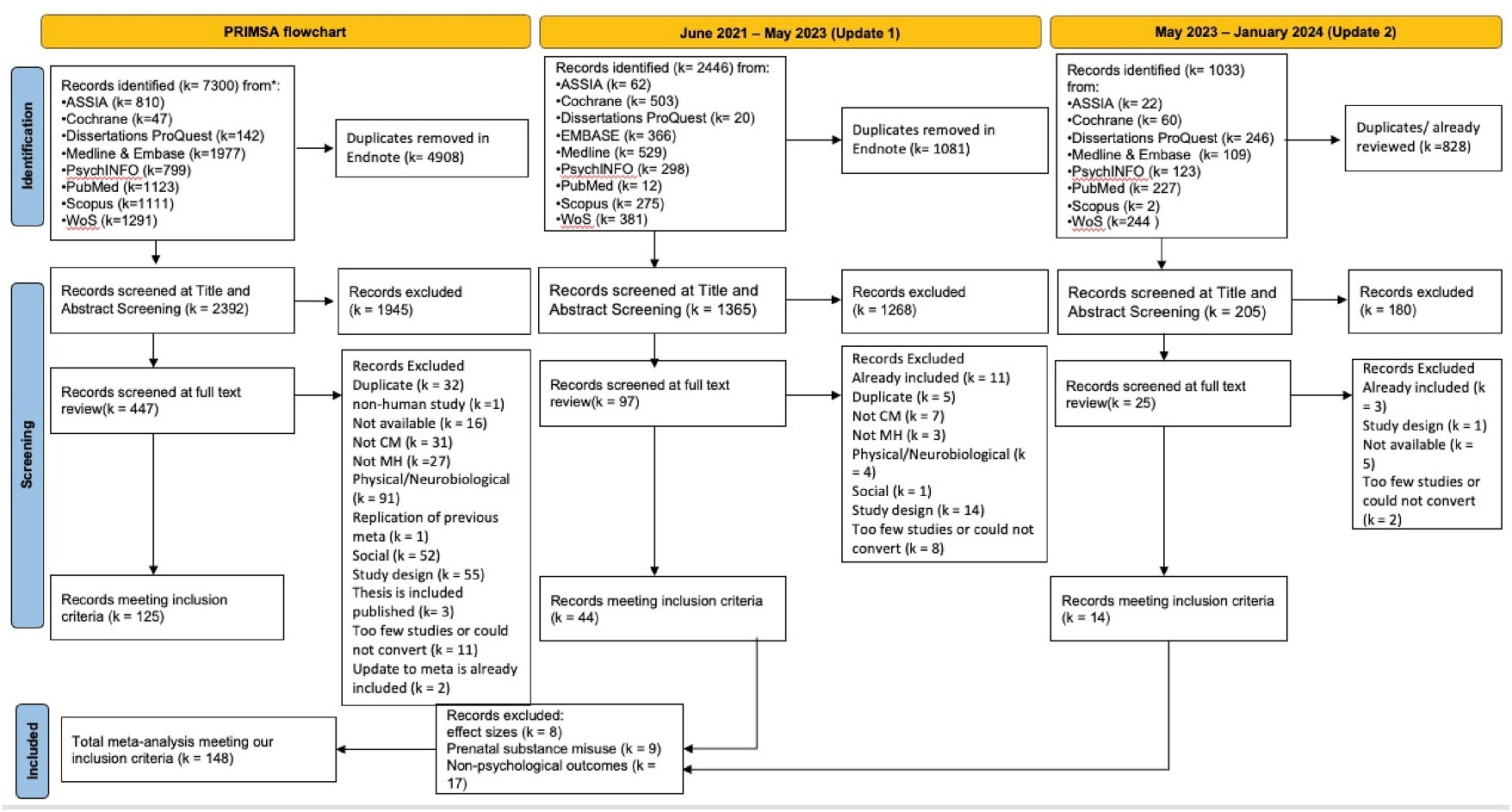
PRISMA flowchart.

Overall, 148 meta‐analyses and 668 effect sizes were included in this umbrella synthesis. A PRISMA flow chart illustrating the process, including reasons for exclusion, is presented in Figure 1. This synthesis focused on quantitative reviews (including *k* > 3 studies). We checked the extent to which meta‐analyses had overlap in the primary studies they drew upon (below or above 70%). Sensitivity analyses were planned when meta‐analyses had over 70% overlap in primary studies, examining the contribution of each meta‐analytic effect size to the overall average effect. Such sensitivity analyses were not required, as overlap never exceeded 70%.

### Data selection, extraction, and quality rating

Authors 1, 4 and 5 conducted title and abstract screening, using a decision flow‐chart (Supporting Information S1: Appendix S3). They independently piloted the flow chart and eligibility criteria on 5% of the records in the initial search. These records were the top 5% articles when sorted alphabetically by title. After pilot screening, Authors 1, 4, 5 independently coded *k* = 2075 citations and abstracts. Author 1 screened all citations. Author 5 provided second coding on *k* = 939 citations and abstracts. Author 4 provided second coding on *k* = 1112 citations and abstracts. An additional *k* = 24 citations and abstracts were coded as brought for discussion and were not included in the calculation of interrater reliability. Interrater reliability between Authors 1 and 5 and Authors 1 and 4 was kappa = 0.69 and kappa = 0.83, respectively. In total, there were *k* = 95 (9%) disagreements between Authors 1, 4, and 5 during the title and abstract screening. To resolve disagreements, they provided the rationale for their decision, and resolved the disagreements by discussion.

Authors 1, 4, and 5 reviewed the articles taken to the full‐text review. Author 6 conducted double coding on 45 full texts. Interrater agreement between authors 5 and 6 was kappa = 0.75 (*k* = 15). Inconsistencies were reviewed by Author 1. There was perfect agreement between authors 4 and 6 (kappa = 1; *k* = 15) and authors 1 and 6 (kappa = 1; *k* = 15). Three research assistants also provided an additional round of checks on all of the extracted effect sizes and numbers of participants.

Quality assessment was conducted using an adjusted version of the framework described by Van IJzendoorn et al. (2020). Nine indicators of quality can be found in Supporting Information S1: Appendix S3. The following indicators coded as 0 (lower quality) or 1 (higher quality) were aggregated in the quality rating: search strategy; sample size; test of homogeneity; publication bias (PB); quality assessment; and independent title and abstract screening; full‐text review; extraction of data; and quality assessment in the meta‐analysis. A score of six or higher on the resulting nine‐point scale may be interpreted as good quality. Interrater agreement between coders on data extraction and quality in the current umbrella synthesis was 90% exact agreement (*k* = 19). Many of the core components of this quality rating are also included in AMSTAR‐2 (Shea et al., 2017) which is a widely used but labour‐intensive instrument.

### Data synthesis and analysis

Data were extracted from 148 meta‐analyses. It was common for meta‐analyses to report more than one effect size for a given class of outcomes. To account for this data structure, we first conducted a meta‐analysis with all effect sizes (i.e., each individual effect sizes from each study included in each meta‐analyses). We then conducted a random effects multi‐level meta‐analysis with effects from the same meta‐analysis included as nested within that meta‐analysis. This model used standard errors and confidence intervals. Confidence intervals were not available for 65 effect sizes. As such, this analysis was based on *k* = 603 effect sizes.

For the remainder of the analyses we included the effect size with the largest total number of individuals when more than one effect size was presented for the same class of outcome (e.g., several effect sizes for externalising problems in one meta‐analysis). This is in line with approaches adopted in previous umbrella syntheses (Guo et al., 2022). The meta‐analyses identified in our search presented associations with a range of effect sizes including *r*, Fisher's *z*, Cohen's *d*, Hedges' *g*, odds ratios and risk ratios. In line with best practice guidance (Fusar‐Poli & Radua, 2018) all effect sizes were transformed to a common statistic to support interpretation. Correlation (*r*) was chosen as the common effect size because it was the most common effect size (310/668) and thus it limited the number of transformations required. In the vast majority of cases, transformations were conducted using Psychometrika ‐ an online converter (Lenhard, 2016). Proportions were also transformed using (MedCalc Software Ltd, 2022) and Fisher's *z* was transformed using an online stats book (Lane, n.d.).

Moderator analyses were conducted to examine whether transformation affected synthesised effect sizes. Transformations that assumed equal sample sizes were flagged for procedural moderator analysis to examine the influence of such transformations on the results of the umbrella synthesis. Efforts were made to extract raw participant numbers from each meta‐analysis for each predictor‐outcome pairing. Yet these data were not available for 213/668 effect sizes. For instance, some meta‐analyses did not report which studies were used in a given subgroup analysis. When the raw number of participants was not available, we used the overall number of participants in the meta‐analysis, estimated the average N per study, and multiplied this by the number of studies relevant to the predictor‐outcome pairing in that meta‐analysis. As above, when the number of participants was estimated this was coded for moderator analyses. We compared this approach to an approach that used standard errors, finding no material difference between the model estimates. For statistical (e.g., *k* < 3) and practical reasons (e.g., data not available) it was not possible to conduct subgroup analysis on gender, ethnicity, economic deprivation, age at reporting CM or mental health difficulties, study design differences (e.g., prospective vs. retrospective reports), and self‐report versus informant report, because such information too often could not be derived from the available meta‐analyses. Overlap in primary studies was calculated for each outcome class. To our knowledge there is no widely agreed threshold for analysing overlap in primary studies when combining meta‐analyses in an umbrella synthesis. In the current umbrella synthesis we set this threshold at 70%. We identified an overlap of over 70% in primary studies in only two meta‐analyses: Ng et al. (2018) and Liu et al. (2018). However, these two meta‐analyses remained in the umbrella synthesis because they reported effect sizes for different outcomes (self‐injury and suicide attempt, respectively). Due to variance in effect sizes between meta‐analyses, this umbrella synthesis used random effects models. Outlier analysis was conducted by checking meta‐analyses with effect sizes that were three standard deviations above or below the average overall effect size. Subgroup analysis was conducted to explore the relations between different forms of maltreatment and different outcomes. However, it is common for studies to report an effect size for ‘overall’, ‘any’ or ‘unspecified’ CM. To avoid overlapping subgroups, the ‘unspecified’ CM category, including studies using ACE measures for CM, was excluded from the subgroup analysis. In line with the inclusion criteria, subgroup analysis was only conducted when there were more than three meta‐analyses for a given predictor‐outcome pairing. As indicated above, subgroup analysis was also conducted on two procedural moderators: transformed effect size (yes or no) and estimated N (yes or no). For reproducibility, we have provided links to all data and R code in Supporting Information S1: Appendix S4. Adjustments to the protocol are discussed in Supporting Information S1: Appendix S5. Drawing from the approach outlined in Harrer et al. (2021), meta‐analytic outcomes were combined and analysed using R version 4.2.0 (R Core Team, 2022). The creation of random effects models and Forest plots was facilitated by the statistical packages ‘meta’ (Balduzzi S, 2019) and ‘metafor’ (Viechtbauer, 2010). Findings were discussed with experts‐by‐experience (i.e., young people with social care involvement) in the United Kingdom to explore whether interpretations of the results were consistent with their lived experience.

Benchmarks established by Funder and Ozer (2019) suggest that 0.1 < *r* < 0.3 point at a moderately strong association (see also Schuengel et al., 2021). These benchmarks were used to aid interpretation of effect sizes in this umbrella synthesis.

## RESULTS

### Overview

We identified 148 meta‐analyses examining the association between CM and mental health and related difficulties. From these 148 meta‐analyses, 668 effect sizes were extracted. The number of primary studies per meta‐analysis ranged between four (e.g., Silva et al., 2018) and 254 (Zhang, Gao, et al., 2022a; Zhang, Wang, et al., 2022b). The number of people per meta‐analytic effect size varied between 181 (Molendijk et al., 2017) and 3,190,071 (Gardner et al., 2019). A description of included studies can be found in Table 2. Additional information included type of assessment, definitions of study design is available in Supporting Information S1: Appendix S6.

**TABLE 2:** Description of included studies by effect sizes for each MH outcome.

Authors	CM	*k*	*N*	Extracted effect sizes	*r*	Outcome	MH group	*i* ^2^ or H	Q/*x* ^2^ (*p* <. 05)	PB tests	PB identified	Quality rating
Augsburger et al. (2019)	CM	34	32,349	*d* = 0.30 [0.20, 0.30]	0.15	Aggression	Externalising	nr	Yes	FP, ET, FSN	No	High
Baldwin et al. (2023)	CM	7	4719	*d* = 0.35 [0.24, 0.46]	0.17	Conduct problems	Externalising	nr	nr	FP, ET	Yes	High
Braga et al. (2017)	CM	26	23,973	*r* = 0.11 [No CIs]	0.11	Antisocial behaviour	Externalising	*I* ^2^ = 72%	Yes	ET, TaF	Yes	L/M
Braga et al. (2018)	CM	14	20,946	OR = 10.96 [10.42, 20.71]	0.18	Antisocial behaviour	Externalising	*I* ^2^ = 93%	Yes	ET, TaF	No	High
Cui and Liu (2020)	EA	8	5939	*d* = 0.23 [0.06, 0.40]	0.12	Aggression	Externalising	*I* ^2^ = 84.7	nr	FSN, FP, TaF	No	High
Evans et al. (2008)	IPV	53	7200	*d* = 0.47 [0.38, 0.56]	0.23	Externalising	Externalising	nr	Yes	nr	Yes	L/M
Fares‐Otero et al. (2023)	CM	6	679	*r* = 0.25 [0.18, 0.33]	0.25	Aggression	Externalising	*I* ^2^ = 0%	Yes	FP, ET, TaF	No	High
Fitton (2017)	ACES	18	62,407	OR = 10.8 [10.4, 20.4]	0.16	Violence	Externalising	*I* ^2^ = 92%	nr	BMT	No	L/M
Gershoff and Grogan‐Kaylor (2016)	PA	14	27,074	*d* = 0.41 [0.32, 0.50]	0.20	Externalising problems	Externalising	*I* ^2^ = 88%	nr	ET	No	High
Godbout et al. (2019)	PA	38	39,196	*r* = 0.21 [0.17, 0.25]	0.21	IPV perpetration	Externalising	*I* ^2^ = 84%	Yes	FP, TaF, FSN	No	L/M
Goncy et al. (2021)	CM	80	36,259	*r* = 0.16 [0.14, 0.17]	0.16	Dating abuse perpetration	Externalising	nr	nr	FP, ET, TaF	No	L/M
Green et al. (2017)	CM	11	2215	OR = 20.46 [10.91, 30.16]	0.24	Violence	Externalising	*I* ^2^ = 0%	Yes	FP, ET	Yes	High
Heerde and Hemphill (2019)	CM	42	11,681	OR = 40.34 [20.99, 60.30]	0.38	Violence	Externalising	*I* ^2^ = 95%	Yes	FP, FSN	Yes	High
Hughes et al. (2017)	ACES	8	27,935	OR = 80.10 [50.87, 110.18]	0.50	Violence perpetration	Externalising	*I* ^2^ = 68%	nr	BMT, ET, FP	Yes	High
Kitzmann et al. (2003)	IPV	45	2520	Average *d* = 0.43*	0.21	Externalising	Externalising	nr	No	nr	Yes	L/M
Lavi et al. (2019)	CM	38	9029	*d* = 0.53 [0.43, 0.62]	0.26	Aggression	Externalising	*I* ^2^ = 61%	Yes	FP, ET, TaF	Yes	L/M
Li, Zhao and Yu (2020)	PA	50	25,829	*r* = 0.17 [0.14, 0.20]	0.17	IPV perpetration	Externalising	nr	nr	FP, FSN, p‐curve	No	L/M
MacMillan and Rind (1999)	SA	5	1497	*r* = 0.11 [0.06, 0.16]	0.11	Hostility	Externalising	*H* = 11.22*	nr	nr	Yes	L/M
Magalhaes and Camilo (2023)	CM	8	2274	*r* = 0.10 [No CIs]	0.10	Externalising	Externalising	nr	nr	TaF	No	L/M
Martijn et al. (2020)	CM	21	3474	*d* = 0.32 [0.39, 0.25]	0.16	Committing sexual offence	Externalising	*I* ^2^ = 0%	No	MA	No	L/M
Mootz et al. (2022)	IPV	6	20,802	*r* = 0.37 [0.26, 0.47]	0.37	IPV perpetration	Externalising	nr	nr	FSN	No	L/M
Neumann et al. (1996)	SA	8	2706	*d* = 0.39 [0.25, 0.51]	0.19	Anger	Externalising	nr	nr	nr	Yes	L/M
Nilsen et al. (2020)	Neg	5	367,471	OR = 10.69 [10.33, 20.07]	0.14	Externalising behaviours	Externalising	*I* ^2^ = 82%	nr	FP, 1/*n* method	Yes	High
Norman et al. (2013)	PA	12	63,109	OR = 20.29 [10.76, 20.97]	0.22	Conduct problems	Externalising	*I* ^2^ = 31%	No	FP	Yes	High
Petruccelli et al. (2019)	CM	6	224,420	OR = 20.00 [10.74, 20.30]	0.19	Behaviour problem	Externalising	nr	nr	nr	Yes	High
Pinquart (2017a)	Neg	98	71,932	*r* = 0.19 [0.17, 0.22]	0.19	Externalising symptoms	Externalising	nr	No	MA, TaF	No	L/M
Ran et al. (2022)	CM	51	30,566	*r* = 0.24 [No CIs]	0.24	Aggressive behaviours	Externalising	nr	nr	FP, ET	No	L/M
Ranu et al. (2022)	PA	8	9610	OR = 20.28 [10.78, 20.92]	0.22	Violence in psychosis	Externalising	*I* ^2^ = 0%	nr	nr	Yes	High
Silva et al. (2018)	IPV	4	11,683	OR = 10.82 [10.28, 20.60]	0.16	Behavioural problems	Externalising	*I* ^2^ = 45%	nr	nr	Yes	High
Smith‐Marek et al. (2015)	IPV	84	89,134	*r* = 0.25 [0.25, 0.27]	0.25	IPV perpetration	Externalising	nr	nr	FSN, TaF	No	High
Vu et al. (2016)	IPV	74	46,121	*r* = 0.15 [No CIs]	0.15	Externalising problems	Externalising	nr	Yes	ET	No	High
Zhu, Racine, et al. (2023); Zhu, Ye, et al. (2023)	ACES	15	36,294	*r* = 0.17 [0.12, 0.22]	0.17	IPV perpetration	Externalising	*I* ^2^ = 95%	Yes	FP	No	L/M
Agnew‐Blais and Danese (2016)	CM	7	5091	OR = 10.90 [10.39, 20.61]	0.17	Anxiety	Internalising	*I* ^2^ = 73%	Yes	FP, ET, BMT, TaF	No	High
Alameda et al. (2021)	CM	13	2750	*r* = 0.24 [0.15, 0.34]	0.24	Depression	Internalising	*I* ^2^ = 82%	Yes	FP, ET, TaF	Yes	High
Amado et al. (2015)	SA	87	123,735	*r* = 0.24 [No CIs]	0.24	Depression	Internalising	nr	nr	nr	Yes	L/M
Baldwin et al. (2023)	CM	13	11,697	*d* = 0.22 [0.14, 0.30]	0.11	Depression	Internalising	nr	nr	FP, ET	Yes	High
Bellis et al. (2019)	ACES	7	91,112	RR = 20.69 [20.17, 30.33]	0.26	Depression	Internalising	*I* ^2^ = 97%	nr	nr	Yes	High
Braithwaite et al. (2017)	CM	13	8990	OR = 10.59 [=10.46, 10.75]	0.13	Depression	Internalising	*I* ^2^ = 80%	nr	FP, TaF	Yes	L/M
Cui and Liu (2020)	EA	16	51,775	*d* = 0.58 [0.56, 0.60]	0.28	Internalising	Internalising	*I* ^2^ = 95%	nr	FSN, FP, TaF	No	High
Evans et al. (2008)	IPV	58	7602	*d* = 0.48 [0.39, 0.57]	0.23	Internalising	Internalising	nr	No	nr	Yes	L/M
Gardner et al. (2019)	SA	17	3,190,071	OR = 10.92 [10.59, 20.31]	0.18	Anxiety	Internalising	*I* ^2^ = 68%	Yes	FP	Yes	L/M
Gershoff and Grogan‐Kaylor (2016)	PA	8	15,899	*d* = 0.24 [0.13, 0.35]	0.12	Internalising	Internalising	*I* ^2^ = 89%	nr	ET	No	High
Gibb (2002)	SA	10	1290	*r* = 0.17 [= 0.12, 0.22]	0.17	Negative cognitive styles	Internalising	H 25.54*	nr	FSN	No	L/M
Hughes et al. (2017)	ACES	13	104,672	OR = 40.40 [30.54, 50.46]	0.18	Depression	Internalising	*I* ^2^ = 80%	nr	BMT, ET, FP	No	High
Humphreys et al. (2020)	EA	81	29,038	Fisher's *z* = 0.38 [No Cis]	0.36	Depression	Internalising	*I* ^2^ = 87%	Yes	ET, TaF	Yes	L/M
Infurna et al. (2016)	EA	6	3120	SMD = 0.500 [0.22, 0.78]	0.24	Depression	Internalising	nr	nr	FSN	No	L/M
Ip et al. (2015)	PA	6	4700	OR = 10.68 [10.36, 20.08]	0.14	Depression	Internalising	*I* ^2^ = 70%	nr	ET, FP	No	High
Islas‐Preciado et al. (2021)	CM	5	3653	OR = 10.99 [10.58, 20.51]	0.19	Menstrual ‐related mood disorders	Internalising	*I* ^2^ = 27%	No	nr	Yes	High
Jumper (1995)	SA	20	3546	*r* = 0.22 [0.21, 0.35]	0.22	Depression	Internalising	nr	Yes	nr	Yes	L/M
Kitzmann et al. (2003)	IPV	47	2632	Average *d* = 0.5*	0.24	Internalising	Internalising	nr	No	nr	Yes	L/M
Lai et al. (2023)	ACES	40	57,422	*r* = 0.24 [0.20, 0.27]	0.24	Depression	Internalising	nr	Yes	FP, ET	No	High
LeMoult et al. (2020)	CM	62	44,066	OR = 20.5 [20.08, 30.00]	0.24	Depression	Internalising	*I* ^2^ = 90%	Yes	BMT, FP	No	L/M
Li et al. (2016)	CM	4	9586	OR = 20.70 [20.10, 30.47]	0.26	Anxiety	Internalising	*I* ^2^ = 55%	nr	ET, FP	No	High
Li, Chu, et al. (2020)	SA	13	8652	*g* = 0.44 [=0.29, 0.58]	0.21	Depression	Internalising	*I* ^2^ = 75%	Yes	FP, ET, FSN, TaF	No	High
Li et al. (2022)	CM	58	32,820	*r* = 0.17 [0.15, 0.18]	0.17	Depression	Internalising	*I* ^2^ = 89%	Yes	FP, ET, TaF	Yes	High
Lindert et al. (2014)	SA	13	85,435	OR = 20.52 [20.12, 20.98]	0.25	Anxiety	Internalising	*I* ^2^ = 59%	nr	FP, BMT	No	L/M
Liu, Deng, et al. (2023)	CM	29	43,982	*r* = 0.20 [0.17, 0.23]	0.20	Social anxiety	Internalising	*I* ^2^ = 77%	Yes	FP, FSN, ET	No	High
MacMillan and Rind (1999)	SA	22	7778	*r* = 0.12 [0.10, 0.14]	0.12	Depression	Internalising	*H* = 25.71	nr	nr	Yes	L/M
Magalhaes and Camilo (2023)	CM	15	4264	*r* = 0.14 [No CIs]	0.14	Internalising	Internalising	nr	nr	TaF, FP	Yes	L/M
Nelson et al. (2017)	SA	57	74,461	OR = 20.66 [20.38, 20.98]	0.26	Depression	Internalising	*I* ^2^ = 65%	Yes	FP, ET, TaF, MA	No	High
Neumann et al. (1996)	SA	24	8118	*d* = 0.41 [0.36, 0.46]	0.20	Depression	Internalising	nr	nr	nr	Yes	L/M
Norman et al. (2013)	PA	59	310,285	OR = 10.51 [10.27, 10.79]	0.11	Anxiety	Internalising	*I* ^2^ = 90%	Yes	FP	Yes	High
Paolucci et al. (2001)	SA	25	6417	*r* = 0.21 [No CIs]	0.21	Depression	Internalising	nr	nr	FDA	Yes	L/M
Petruccelli et al. (2019)	CM	14	523,648	OR = 30.16 [20.81, 30.54]	0.30	Depression	Internalising	nr	nr	nr	Yes	High
Pinquart (2017b)	Neg	95	69,730	*r* = 0.14 [0.11, 0.17]	0.14	Internalising symptoms	Internalising	nr	nr	MA, TaF	Yes	L/M
Racine et al. (2021)	CM	12	6116	*r* = 0.19 [0.13, 0.24]	0.19	Prenatal depressive symptoms	Internalising	*I* ^2^ = 74%	Yes	FP, ET, TaF	Yes	High
Shamblaw et al. (2019)	PA	35	24,580	*r* = 0.27 [0.243, 0.299]	0.27	Prenatal depressive symptoms	Internalising	nr	nr	FSN	No	High
Silva et al. (2018)	IPV	4	2943	OR = 20.10 [10.17, 30.76]	0.20	Internalising problems	Internalising	*I* ^2^ = 21%	nr	nr	Yes	High
Souama et al. (2023)	CM	13	217,929	OR = 20.82 [20.40, 30.30]	0.28	Depression	Internalising	*I* ^2^ = 92%	Yes	nr	Yes	L/M
Tan and Mao (2023)	ACES	9	14,238	OR = 20.75 [20.26, 30.34]	0.27	Depression	Internalising	*I* ^2^ = 69%	Yes	FP, ET	No	High
Tang et al. (2019)	CM	6	9938	*r* = 0.33 [0.25, 0.40]	0.33	Depression	Internalising	*I* ^2^ = 94%	nr	ET	No	High
Vibhakar et al. (2019)	CM	30	17,186	*d* = 0.51 [0.41, 0.61]	0.25	Depression	Internalising	*I* ^2^ = 93%	Yes	FP,	Yes	L/M
Vu et al. (2016)	IPV	74	46,121	*r* = 0.10 [No CIs]	0.10	Internalising problems	Internalising	nr	Yes	ET, FSN	No	High
Wang et al. (2022)	PA	9	20,910	OR = 10.73 [10.55, 10.95]	0.15	Elder depression	Internalising	*I* ^2^ = 95%	Yes	FSN, ET	No	High
Watters et al. (2023)	ACES	16	18,732	*r* = 0.26 [0.17, 0.35]	0.26	Depression	Internalising	*I* ^2^ = 98%	nr	ET	No	L/M
Yu et al. (2017)	SA	17	21,457	OR = 20.46 [10.94, 30.12]	0.24	Depression	Internalising	*I* ^2^ = 87%	Yes	FP, FSN, ET	No	L/M
Zhang et al. (2021)	CM	34	192,182	OR = 20.85 [ 20.03, 30.66]	0.28	Panic disorder	Internalising	*I* ^2^ = 93%	nr	FP	Yes	L/M
Mandelli et al. (2015)	SA	14	23,087	OR = 20.42 [10.94, 30.02]	0.24	Depression	Internalising	*I* ^2^ = 70%	nr	ET	Yes	L/M
Agnew‐Blais and Danese (2016)	CM	14	5733	OR 10.85 [10.43, 20.40]	0.17	Bi‐polar disorder	Thought problems	*I* ^2^ = 69	Yes	FP, ET, BMT, TaF	No	High
Alameda et al. (2021)	CM	27	4762	*r* = 0.16 [0.12, 0.20]	0.16	Positive symptom (psychosis)	Thought problems	*I* ^2^ = 39%	Yes	FP, ET, TaF	No	High
Bailey et al. (2018)	ACES	18	3857	*r* = 0.14 [No CIs]	0.14	Positive symptoms	Thought problems	*I* ^2^ = 40%	Yes	FP, ET, FSN	No	High
Baldwin et al. (2023)	CM	4	692	*d* = 0.34 [0.17, 0.51]	0.17	Psychosis	Thought problems	nr	nr	FP, ET	Yes	High
Bodicker et al. (2022)	CM	12	15,481	*r* = 0.21 [0.19, 0.23]	0.21	Body image	Thought problems	*I* ^2^ = 72%	nr	FP, BMT, ET	No	High
Boumpa et al. (2022)	SA	24	19,867	OR = 30.60 [20.75, 40.73]	0.33	PTSD	Thought problems	nr	nr	ET	No	L/M
Brewin et al. (2000)	CM	9	1746	*r* = 0.14 [0.07, 0.30]	0.14	PTSD	Thought problems	Not indicated	nr	nr	Yes	High
Caslini et al. (2016)	SA	25	8789	OR = 20.73 [10.96, 30.79]	0.27	Bulimia	Thought problems	*I* ^2^ = 62%	Yes	FP, ET, TaF	No	High
Cri»ôan et al. (2023)	ACES	40	80,575	*r* = 0.16, [0.118; 0.200]	0.16	Avoidant	Thought problems	*I* ^2^ = 91%	Yes	FP, ET, TaF	Yes	L/M
De Ruiter et al. (2022)	CM	32	8672	*r* = 0.20 [0.16, 0.24]	0.20	Psychopathy	Thought problems	*I* ^2^ = 57%	Yes	FP, ET, TaF	No	High
Dolan (2018)	SA	5	18,385	OR = 20.37 [10.37, 40.09]	0.23	Auditory hallucinations	Thought problems	*I* ^2^ = 81%	Yes	nr	Yes	L/M
Fossati et al. (1999)	SA	21	2479	*r* = 0.279 [ 0.242, 0.315]	0.28	BPD personality disorder	Thought problems	*X* ^2^ = 28.7	nr	nr	Yes	L/M
Gao, Yu, et al. (2023)	CM	15	9141	*r* = 0.20 [No CIs]	0.20	Vulnerable narcissism	Thought problems	nr	nr	FP, TaF	Yes	L/M
Gardner et al. (2019)	SA	11	45,219	OR = 30.54 [20.31, 50.41]	0.33	PTSD	Thought problems	*I* ^2^ = 89%	Yes	FP	Yes	L/M
Ip et al. (2016)	PA	12	9400	OR = 20.62 [20.13, 30.22]	0.26	Axis ii mental health disorders	Thought problems	I2 = < 50%	nr	ET, FP	No	High
Lee et al. (2022)	ACES	15	4438	*r* = 0.25 [0.21, 0.29]	0.25	BPD	Thought problems	*I* ^2^ = 42%	No	FP, p‐curve, FSN	No	L/M
Leiva‐Bianchi et al. (2023)	SA	12	9217	OR = 20.88 [20.05, 40.05]	0.28	Complex PTSD	Thought problems	*I* ^2^ = 100%	Yes	FSN	No	High
Longobardi et al. (2022)	ACES	57	4180	*r* = 0.28 [0.21, 0.35]	0.22	Body dysmorphic disorder	Thought problems	*I* ^2^ = 53%	Yes	FP, ET	No	High
MacMillan and Rind (1999)	SA	10	2998	*r* = 0.06 [0.02, 0.10]	0.06	Eating disorder	Thought problems	*H* = 9.92	nr	nr	Yes	L/M
Matheson et al. (2013)	CM	7	1681	OR = 30.60 [20.08, 60.23]	0.33	Schizophrenia	Thought problems	*I* ^2^ = 65%	nr	ET test	No	High
Molendijk et al. (2017)	CM	96	34,521	OR = 20.47 [20.15, 20.84]	0.24	Any eating disorder	Thought problems	*I* ^2^ = 57%	nr	ET, TaF	No	High
Neumann et al. (1996)	SA	7	2368	*D* = 0.34 [0.22, 0.46]	0.17	Obsessions and compulsions	Thought problems	nr	nr	nr	Yes	L/M
Norman et al. (2013)	PA	6	31,554	OR = 20.58 [10.17, 50.70]	0.25	Eating disorder	Thought problems	*I* ^2^ = 89%	Yes	FP	Yes	High
Ou et al. (2021)	EA	6	1246	Fisher's *z* = 0.11 [0.03, 0.19]	0.11	Obsessive compulsive disorer	Thought problems	*I* ^2^ = 43%	nr	FP	No	High
Palmier‐Claus et al. (2016)	ACES	19	78,737	OR = 20.63 [20.00, 30.47]	0.26	Bi‐polar disorder	Thought problems	*I* ^2^ = 77%	Yes	FP, ET, TaF	No	High
Paolucci et al. (2001)	SA	26	6860	*r* = 0.20 [No CIs]	0.20	PTSD	Thought problems	nr	nr	FDA	Yes	L/M
Pastore et al. (2022)	CM	15	85,006	OR = 20.20 [10.72, 20.81]	0.21	Psychosis	Thought problems	*I* ^2^ = 82%	Yes	FP, ET, BMT, FSN, TaF	Yes	High
Peh et al. (2019)	SA	5	1108	OR = 10.95 [0.99, 30.84]	0.18	Psychosis	Thought problems	*I* ^2^ = 69%	nr	nr	Yes	High
Porter et al. (2018)	PA	16	866	OR = 10.27 [No CIs]	0.54	BPD	Thought problems	*I* ^2^ = 51%	Yes	FP, ET, TaF	Yes	High
Porter et al. (2020)	SA	33	5736	OR = 60.60 [50.15, 80.47]	0.46	BPD	Thought problems	*I* ^2^ = 64%	Yes	FP, ET, TaF	Yes	High
Rafiq et al. (2018)	Neg	36	2639	*r* = 0.19 [0.14, 0.24]	0.19	Overall dissociation	Thought problems	*I* ^2^ = 44%	Yes	FP, ET, TaF	Yes	L/M
Smolak and Murnen (2002)	SA	30	13,145	*r* = 0.18 [No CIs]	0.18	Eating problems	Thought problems	nr	Yes	nr	Yes	L/M
Toutountzidis et al. (2022)	PA	13	7335	*r* = 0.20 [0.16, 0.25]	0.20	Schizotypy	Thought problems	*I* ^2^ = 73%	Yes	TaF	Yes	High
Trentacosti and Little (2021)	CM	13	4563	Fisher's *Z* = 0.34 [0.24, 0.37]	0.33	BPD	Thought problems	nr	Yes	nr	Yes	L/M
Trotta et al. (2015)	ACES	9	13,887	OR = 10.73 [10.26, 20.20]	0.15	Psychotic experiences	Thought problems	*I* ^2^ = 36%	nr	MA	No	L/M
Varese et al. (2012)	ACES	36	81,253	OR = 20.78 [=20.34, 30.31]	0.27	Psychosis	Thought problems	*I* ^2^ = 73%	nr	ET test	No	High
Vonderlin et al. (2018)	CM	32	8279	*d* = 0.53 [0.43, 0.62]	0.26	Dissociation	Thought problems	*I* ^2^ = 53%	Yes	MA	Yes	L/M
Winsper et al. (2016)	EA	7	6493	OR = 30.28 [20.67, 40.03]	0.31	BPD	Thought problems	*I* ^2^ = 0	No	FP	No	High
Agnew‐Blais Danese (2016)	CM	13	3422	OR 20.25 [10.88, 20.70]	0.22	Suicidal distress	Suicidal distress	*I* ^2^ = 0%	No	FP, ET, BMT, TaF	No	High
Angelakis et al. (2019)	SA	36	210,763	OR = 30.17 [20.76, 30.64]	0.30	Suicide attempts	Suicidal distress	*I* ^2^ = 68%	nr	FP, ET, TaF	No	High
Angelakis et al. (2020a)	CM	13	10,219	OR 30.09 [20.14, 40.45]	0.30	Suicide attempts	Suicidal distress	*I* ^2^ = 92%	nr	FP, ET, TaF	Yes	High
Angelakis et al. (2020b)	SA	48	253,638	OR = 30.41 [20.90, 40.00]	0.32	Suicide attempts	Suicidal distress	*I* ^2^ = 97%	nr	FP, ET, TaF	No	High
Baldini et al. (2023)	ACES	11	5639	OR = 10.92 [10.51, 20.45]	0.18	Suicidal distress	Suicidal distress	*I* ^2^ = 59%	nr	ET	Yes	High
Baldwin et al. (2023)	CM	4	5932	*d* = 0.40 [0.25, 0.54]	0.20	Suicidal ideation	Suicidal distress	nr	nr	FP, ET	Yes	High
Castellvi et al. (2017)	CM	32	43,622	OR = 20.25 [10.85, 20.73]	0.22	Suicide attempts	Suicidal distress	*I* ^2^ = 88%	nr	FP, ET, TaF	Yes	L/M
Duarte et al. (2020)	Neg	6	1033	*g* = 0.18 [No Cis]	0.09	Suicide attempt	Suicidal distress	*I* ^2^ = 0	No	FP, FSN, ET	No	High
Liu et al. (2017)	Neg	10	3371	SMD = 0.26 [0.07, 0.45]	0.13	Risk of suicidal behaviour	Suicidal distress	nr	Yes	BMT	No	High
Liu et al. (2018)	SA	63	48,246	OR = 2·65, 2·33, 3·03, *p* < ·0001	0.26	Non‐suicidal self‐injury	Suicidal distress	*I* ^2^ = 69%	nr	FSN, TaF, ET	Yes	L/M
MacMillan and Rind (1999)	SA	9	5425	*r* = 0.09 [0.06, 0.12]	0.09	Suicide	Suicidal distress	*H* = 10.94	nr	nr	Yes	L/M
Neumann et al. (1996)	SA	8	2706	*d* = 0.34 [0.24, 0.44]	0.17	Suicidality	Suicidal distress	nr	nr	nr	Yes	L/M
Ng et al. (2018)	SA	47	151,476	OR = 10.89 [0.66, 20.12]	0.17	Suicide attempt	Suicidal distress	*I* ^2^ = 84%	Yes	FP, ET	Yes	L/M
Norman et al. (2013)	PA	58	305,026	OR = 30.00 [20.07, 40.33]	0.29	Suicide	Suicidal distress	*I* ^2^ = 98%	Yes	nr	Yes	High
Paolucci et al. (2001)	SA	10	4008	*r* = 0.21 [No CIs]	0.21	Suicide	Suicidal distress	nr	nr	FDA	Yes	L/M
Petruccelli et al. (2019)	CM	6	224,420	OR = 70.30 [40.33, 120.3]	0.48	Suicidal ideation	Suicidal distress	nr	nr	nr	Yes	High
Witt et al. (2019)	SA	5	643	OR = 10.52 [10.02, 20.28]	0.11	Suicide behaviours	Suicidal distress	*I* ^2^ = 0%	nr	nr	Yes	High
Xiao et al. (2023)	EA	9	38,504	*d* = 0.48 [0.16, 0.80]	0.23	Suicidal ideation or attempt	Suicidal distress	*I* ^2^ = 12%	No	TaF	Yes	High
Zatti et al. (2017)	SA	6	11,607	OR = 30.73, [20.94, 40.75]	0.34	Suicide attempt	Suicidal distress	*I* ^2^ = 68%	nr	BMT, ET, TaF	No	L/M
Agnew‐Blais and Danese (2016)	CM	11	5469	OR = 10.84 [10.41, 20.39]	0.17	Substance misuse	SUD	*I* ^2^ = 61%	Yes	FP, ET, BMT, TaF	No	High
Baldwin et al. (2023)	CM	7	23,863	*d* = 0.29 [0.17, 0.41]	0.14	Alcohol abuse	SUD	nr	nr	FP, ET	Yes	High
Bellis et al. (2019)	ACES	5	85,745	RR = 1.81 [1.22, 2.68]	0.16	Harmful alcohol use (USA)	SUD	*I* ^2^ = 97%	nr	nr	Yes	High
De la Pena‐Arteaga et al. (2021)	SA	6	24,710	OR = 10.29 [10.08, 10.49]	0.07	Cannabis use	SUD	nr	nr	nr	Yes	High
Gershoff and Grogan‐Kaylor (2016)	PA	4	7392	*d* = 0.13 [0.08, 0.35]	0.07	Substance misuse (adulthood)	SUD	*I* ^2^ = 92%	nr	ET	No	High
Halpern et al. (2018)	SA	7	20,172	OR = 10.73 [10.24, 20.41]	0.15	Illicit substance use	SUD	*I* ^2^ = 42%	nr	ET, BMT, TaF	No	High
Hughes et al. (2017)	ACES	10	33,992	OR = 50.84 [30.99, 80.56]	0.44	Problematic alcohol use	SUD	*I* ^2^ = 80%	nr	BMT, ET, FP	No	High
Lucia et al. (2020)	SA	4	9578	OR = 20.35 [10.64, 30.35]	0.23	Cannabis abuse	SUD	*I* ^2^ = 78%	nr	nr	Yes	L/M
MacMillan and Rind (1999)	SA	8	1645	*r* = 0.07 [0.02, 0.12]	0.07	Alcohol problem	SUD	*H* = 2.97	nr	nr	Yes	L/M
Neumann et al. (1996)	SA	14	4735	*d* = 0.41 [0.31, 0.51]	0.20	Substance abuse	SUD	nr	nr	nr	Yes	L/M
Norman et al. (2013)	PA	43	226,140	OR = 10.92 [10.67, 20.20]	0.18	Drug use	SUD	*I* ^2^ = 69%	Yes	nr	Yes	High
Petruccelli et al. (2019)	CM	18	673,261	OR = 40.31 [30.90, 40.76]	0.37	Alcohol problem	SUD	nr	nr	nr	Yes	High
Zhu, Racine, et al. (2023)	ACES	43	380,198	OR = 20.19 [10.98, 20.43]	0.31	Drug use	SUD	*I* ^2^ = 96%	Yes	FP, TaF	Yes	High
Amado et al. (2015)	SA	91	125,555	*r* = 0.28 [No CIs]	0.28	Any mental health difficulty	Other psychological	nr	nr	nr	Yes	L/M
Baer and Martinez (2006)	CM	8	791	OR = 70.02 [50.10, 90.67]	0.47	Insecure attachment	Other psychological	nr	nr	nr	Yes	L/M
Baldwin et al. (2023)	ACES	5	26,574	*d* = 0.15 [0.02, 0.31]	0.15	Any mental health difficulty	Other psychological	nr	nr	FP, ET	Yes	High
Beaumont (2018)	IPV	49	14,963	*r* = 0.26 [0.22, 0.30]	0.26	Children's trauma symptoms	Other psychological	*I* ^2^ = 75%	Yes	FP, ET, FSN, TaF	No	L/M
Carmichael (2019)	SA	17	19,836	*r* = 0.16 [0.11, 0.23]	0.16	Paranoia	Other psychological	*I* ^2^ = 88%	nr	FP, LFK, TaF	Yes	L/M
Christy et al. (2023)	CM	28	6679	*r* = 0.11 [0.05, 0.16 ]	0.11	General functioning	Other psychological	*I* ^2^ = 73%	nr	FP, ET, TaF	No	High
Croft et al. (2021)	ACES	14	12,691	SMD = 0.40 [No Cis]	0.20	Locus of control	Other psychological	*I* ^2^ = 94%	nr	FP, ET	Yes	High
Cyr et al. (2010)	CM	10	456	*d* = 20.10 [10.82, 20.37]	0.72	Insecure attachment	Other psychological	nr	Yes	FP	No	L/M
Ditzer et al. (2023)	CM	75	36,141	*r* = 0.28 [0.17, 0.39]	0.23	Alexithymia	Other psychological	nr	No	ET, FP	No	High
Downing and Downing (2022)	CM	5	1423	*r* = 0.31 [0.36, 0.26]	0.31	Self‐compassion	Other psychological	*I* ^2^ = 100%	nr	FP	Yes	High
Fares‐Otero et al. (2023)	CM	7	975	*r* = 0.08 [−0.29, 0.13]	0.08	Social functioning	Other psychological	*I* ^2^ = 90%	Yes	FP, ET, TaF	No	High
Francis et al. (2023)	CM	6	2982	*r* = 0.16 [0.09, 0.22]	0.16	Any mental health difficulty	Other psychological	nr	nr	ET	No	High
Gao, Assinl, et al. (2023)	CM	24	5335	*r* = 0.23 [No CIs]	0.23	Rejection sensitivity	Other psychological	nr	nr	FP, TaF	Yes	High
Gershoff and Grogan‐Kaylor (2016)	PA	10	5122	*d* = 0.53 [0.42, 0.64]	0.27	Any mental health difficulty	Other psychological	*I* ^2^ = 76%	nr	ET	No	High
Gruhn and Compas (2020)	CM	8	4823	*r* = 0.25 [0.15, 0.35]	0.25	Avoidance	Other psychological	*I* ^2^ = 91%	Yes	FP, ET, TaF	No	L/M
Jumper (1995)	SA	23	6878	*r* = 0.27 [0.20, 0.32]	0.27	Any mental health difficulty	Other psychological	nr	Yes	nr	Yes	L/M
Kane and Bornstein (2018)	CM	14	38,265	*d* = 0.29 [No CIs]	0.14	Psychological dependence	Other psychological	nr	nr	FSN	Yes	L/M
Kautz‐Turnbull and Petrenko (2021)	Neg	23	4402	*d* = 10.12 [0.75, 10.49]	0.49	Social functioning	Other psychological	nr	Yes	ET	No	L/M
Khaleque (2015)	Neg	33	11,755	*r* = 0.45 [No CIs]	0.45	Psychological maladjustment	Other psychological	nr	No	FSN	No	L/M
Khan and Jaffee (2022)	CM	90	42,744	*r* = 0.22 [0.18, 0.25]	0.22	Alexithymia	Other psychological	*I* ^2^ = 92%	nr	FP, ET, TaF	No	High
Kim et al. (2021)	CM	56	19,946	Fisher's *Z* = 0.22 [0.19, 0.26]	0.22	Anxious attachment	Other psychological	*I* ^2^ = 80%	nr	FP, ET	No	High
Kitzmann et al. (2003)	IPV	33	1848	*d* = 0.35 [No CIs]	0.17	Any mental health difficulty	Other psychological	nr	No	nr	Yes	L/M
Lavi et al. (2019)	CM	38	10,099	*d* = 0.42 [0.32, 0.51]	0.21	Dysregulation	Other psychological	*I* ^2^ = 69%	Yes	FP, ET, TaF	Yes	L/M
Liu, Teh, et al. (2023)	ACES	74	304,093	*r* = 0.22 [0.19, 0.25]	0.22	Any mental health difficulty	Other psychological	nr	Yes	nr	Yes	High
Luke and Banerjee (2013)	CM	19	6155	*d* = 0.70 [0.40, 0.99]	0.33	Social understanding	Other psychological	nr	Yes	FSN	Yes	L/M
MacMillan and Rind (1999)	SA	20	7723	*r* = 0.09 [0.07, 0.11]	0.09	Sexual problems	Other psychological	*H* = 39.49*	nr	nr	Yes	L/M
McIntosh et al. (2019)	IPV	6	3394	*r* = 0.23 [0.04, 0.42]	0.23	Attachment security	Other psychological	nr	Yes	FP, TaF	Yes	L/M
McKay et al. (2021)	Neg	5	20,624	OR = 10.93 [10.31, 20.85]	0.18	Any mental health difficulty	Other psychological	*I* ^2^ = 58%	nr	FP, ET	Yes	L/M
Mitiku et al. (2024)	SA	4	2319	OR = 50.65 [40.32, 70.39]	0.43	Any mental health difficulty	Other psychological	*I* ^2^ = 0%	nr	ET	Yes	High
Neumann et al. (1996)	SA	16	5412	*d* = 0.36 [0.30, 0.42]	0.18	Sexual problems	Other psychological	nr	nr	nr	Yes	L/M
Noonan and Pilkington (2020)	IPV	10	3931	*r* = 0.22 [0.12, 0.32]	0.22	Attachment security	Other psychological	*I* ^2^ = 83%	nr	FP, ET	No	High
Petruccelli et al. (2019)	CM	5	187,017	OR = 20.67 [20.00, 30.55]	0.26	Poor quality of life	Other psychological	nr	nr	nr	Yes	High
Pilkington et al. (2021)	SA	14	3380	*r* = 0.21 [0.12, 0.30]	0.21	Abandonment	Other psychological	*I* ^2^ = 76%	nr	nr	Yes	High
Pinquart and Gerke (2019)	Neg	28	12,977	*r* = 0.28 [0.22, 0.34]	0.28	Self‐esteem	Other psychological	nr	No	ET, TaF	Yes	High
Pinquart and Fischer (2021)	Neg	4	877	*r* = 0.10 [0.09, 0.27]	0.10	Moral reasoning	Other psychological	*I* ^2^ = 65%	Yes	TaF	Yes	L/M
Tetik and Yalçınkaya Alkar (2021)	SA	12	1242	OR = 10.55 [10.14, 20.10]	0.12	Diagnosis of vaginismus	Other psychological	*I* ^2^ = 0%	nr	FP, TaF, ET	No	High
Todorov et al. (2023)	CM	29	9894	*r* = 0.23 [0.17, 0.30]	0.23	Callous‐unemotional traits	Other psychological	*I* ^2^ = 81%	nr	FP, 1/*n* method, FSN	No	High
Vu et al. (2016)	IPV	74	46,121	*r* = 0.15 [No CIs]	0.15	General functioning	Other psychological	nr	Yes	ET	No	High
Wang et al. (2022)	SA	15	14,619	OR = 10.68 [10.49, 10.87]	0.14	Sexual problems	Other psychological	*I* ^2^ = 21%,	nr	BMT, ET, FP	No	High
Wilson et al. (2010)	CM	24	1868	*d* = 0.29 [0.12, 0.46]	0.14	Aversive	Other psychological	nr	Yes	FSN	No	L/M
Wolfe et al. (2003)	IPV	41	3575	Fisher's *Z* = 0.28 [0.21, 0.32]	0.27	Any mental health difficulty	Other psychological	nr	nr	nr	Yes	L/M
Xiao et al. (2023)	EA	8	28,580	*d* = 0.36 [0.04, 0.69]	0.18	Depression or anxiety	Other psychological	*I* ^2^ = 25%	No	TaF	Yes	High
Yang and Huang (2024)	CM	23	3910	*r* = 0.21 [ 0.15, 0.26]	0.21	Mentalising	Other psychological	*I* ^2^ = 64%	Yes	FSN	No	High
Yeo et al. (2024)	ACES	90	206,354	*r* = 0.32 [0.01, 0.44]	0.32	Emotional wellbeing	Other psychological	*I* ^2^ = 95%	Yes	FP	Yes	High
Yu et al. (2022)	SA	8	99,907	OR = 10.21 [10.12, 10.31]	0.05	Sleep	Other psychological	*I* ^2^ = 81%	nr	nr	Yes	High
Zhang, Gao, et al. (2022)	CM	254	226,656	*r* = 0.19 [0.18, 0.20]	0.19	Self‐esteem	Other psychological	*I* ^2^ = 94%	Yes	FP, FAT, PET	No	High
Zhang Gao, et al. (2023)	CM	24	22,580	*r* = 0.15 [0.13, 0.17]	0.15	Empathy	Other psychological	*I* ^2^ = 91%	nr	FP, FSN, ET, TaF, FP, FAT	No	High
Zhang, Li, et al. (2023)	EA	9	3979	*r* = 0.28 [0.25, 0.31]	0.28	Compassion	Other psychological	*I* ^2^ = 70%	Yes	FSN, FP, TaF, ET, BMT	No	L/M
Zhang, Zhang, et al. (2023)	CM	31	55,585	*r* = 0.23 [No Cis]	0.23	Internet addiction	Other psychological	Indicated	nr	ET, FP	No	High
Zhu, Ye, et al. (2023)	CM	16	13,818	*r* = 0.311 [No Cis]	0.31	Gratitude	Other psychological	*I* ^2^ = 44%	Yes	FP	No	L/M

*Note*: *k*, number of meta‐analyses; Q/*x* ^2^ (*p* < .05) indicates significant Cochran's *Q* or Chi‐square test of heterogeneity.

Abbreviations: BMT, Begg‐Mazumdar test; EA, emotional abuse; ET, Egger's test; FAT, Funnel Asymmetry Test; FP, Funnel plot; FSN, Fail safe number; IPV, intimate partner violence; L/M, low/moderate quality; LFK, LFK Index Test for Asymmetry; MA, moderator analysis or meta‐regression; Neg, neglect; nr, not reported, PA, physical abuse; PB, publication bias; PET, Precision Effect Test; SA, sexual abuse; TaF, trim and fill.

Most meta‐analyses (*k* = 139/148; 94%) conducted a systematic search of the literature and included some form of structured quality assessment (*k* = 99/148; 69%). There was, however, variation regarding screening; for example, *k* = 72/148 (49%) and *k* = 75/148 (51%) of meta‐analyses respectively, carried out title and abstract screening and full‐text review with two or more coders. Regarding data extraction, *k* = 108/148 (73%) and *k* = 78/148 (53%) meta‐analyses conducted data extraction and quality assessment with two or more coders. In terms of associations, the majority of individual effect sizes (*k* = 579/668; 87%) were generated based on data from over 1000 participants. Heterogeneity of effect sizes tended to be high (significant *Q* or *I* ^2^ above 50%) or was not reported for the majority of associations (*k* = 489/668; 73%). Indications of PB were identified or were not reported in just over half the effect sizes (*k* = 338/668; 51%). Overall, 83 (56%) of meta‐analyses were regarded as high quality (scores six or higher). The procedural moderators are explained in Supporting Information S1: Appendix S7 and quality ratings are presented in Supporting Information S1: Appendix S8.

### Umbrella synthesis

Based on all extracted effect sizes (*k* = 668), an association of *r* = 0.21 (95% CI = 0.20–0. 21) between CM and mental health difficulties was identified. A random effects multi‐level meta‐analysis (*k* = 603) estimated an association of *r* = 0.21 (95% CI = 0.19–0.22) with between‐study variance of *σ*2 = 0.0015 and within‐study variances of *σ*2 < 0.0001. Therefore, we did not identify strong evidence that differences between or within studies contributed to significant variation.

The effect size generated by the largest number of participants in each meta‐analysis was then examined, resulting in an association of *r* = 0.23 (95% CI 0.22–0.25). We did not identify a statistically significant difference between maltreatment types (*Q* = 9.41, df = 4, *p* = 0.05). Model summaries detailing effect sizes for each type of maltreatment are presented in Table 3.

**TABLE 3:** Child maltreatment types and any MH difficulty.

Type of maltreatment	*k*	*r*	95% CI (L)	95% Ci (U)	Tau^2^	Tau	*I* ^2^
Emotional abuse	64	0.25	0.22	0.28	0.02	0.14	99.40%
Neglect	77	0.20	0.18	0.23	0.01	0.11	99.20%
Physical abuse	80	0.19	0.17	0.21	0.01	0.09	99.40%
Sexual abuse	97	0.20	0.18	0.22	0.01	0.09	99.50%
Intimate partner violence	22	0.20	0.17	0.23	0.01	0.07	99.20%

*Note*: *k* = number of meta‐analyses.

Next, we examined the associations between CM and the specific mental health difficulties. CM was associated with all examined outcomes (Table 4), with the weakest effect size identified for substance misuse, *r* = 0.19 (95% CI = 0.14–0.26) and the largest effect size for thought problems *r* = 0.24 (95% CI = 0.21–0.27). Findings for subgroup analyses on the type of CM and specific outcomes are presented in Table 5.

**TABLE 4:** Child maltreatment and specific mental health difficulties.

	*k*	*r*	95% CI (L)	95% CI (U)	Tau^2^	Tau	*I* ^2^
Externalising	32	0.21	0.18	0.24	0.01	0.09	99.5%
Internalising	46	0.22	0.20	0.24	0.01	0.07	99.8%
Thought problems	38	0.24	0.21	0.27	0.01	0.10	98.5%
Suicidal distress	19	0.23	0.18	0.28	0.01	0.11	99.9%
Substance use	13	0.19	0.13	0.26	0.01	0.12	99.9%
Other psychological	50	0.24	0.20	0.28	0.02	0.14	99.5%

**TABLE 5:** Child maltreatment types and specific mental health difficulties.

Mental health difficulty	Child maltreatment type	*k*	*r*	95% CI (L)	95% CI (U)	Tau^2^	Tau	*I* ^2^	*p* value
Externalising	Emotional abuse	7	0.16	0.08	0.24	0.01	0.08	97.30%	0.10
	Neglect	10	0.14	0.11	0.17	0.00	0.04	97.60%	
	Physical abuse	11	0.18	0.15	0.22	0.00	0.05	98.40%	
	Sexual abuse	11	0.16	0.13	0.18	0.00	0.03	86.20%	
	Intimate partner violence	8	0.21	0.14	0.28	0.01	0.09	99.30%	
Internalising	Emotional abuse	18	0.26	0.23	0.30	0.00	0.07	98.80%	0.01
	Neglect	16	0.22	0.17	0.27	0.01	0.10	99.20%	
	Physical abuse	21	0.18	0.14	0.21	0.00	0.07	99.20%	
	Sexual abuse	22	0.20	0.18	0.22	0.00	0.04	99.10%	
	Intimate partner violence	7	0.19	0.14	0.24	0.00	0.06	97.60%	
Thought problems	Emotional abuse	14	0.33	0.21	0.44	0.05	0.23	99.40%	0.57
	Neglect	16	0.25	0.17	0.33	0.03	0.16	99.00%	
	Physical abuse	19	0.26	0.20	0.31	0.02	0.13	97.80%	
	Sexual abuse	25	0.24	0.20	0.29	0.01	0.12	98.40%	
	Intimate partner violence	nr	nr	nr	nr	nr	nr	nr	
Suicidal distress	Emotional abuse	8	0.26	0.22	0.29	0.00	0.04	98.40%	<0.01
	Neglect	8	0.16	0.12	0.20	0.00	0.04	90.70%	
	Physical abuse	9	0.24	0.19	0.29	0.00	0.06	98.90%	
	Sexual abuse	13	0.23	0.18	0.28	0.01	0.09	99.60%	
	Intimate partner violence	nr	nr	nr	nr	nr	nr	nr	
Substance use	Emotional abuse	nr	nr	nr	nr	nr	nr	nr	0.59
	Neglect	nr	nr	nr	nr	nr	nr	nr	
	Physical abuse	5	0.12	0.07	0.18	0.00	0.04	98.30%	
	Sexual abuse	5	0.15	0.05	0.24	0.01	0.07	98.30%	
	Intimate partner violence	nr	nr	nr	nr	nr	nr	nr	
Other psychological	Emotional abuse	11	0.20	0.15	0.25	0.01	0.07	99.10%	<0.01
	Neglect	15	0.25	0.18	0.31	0.01	0.12	99.10%	
	Physical abuse	13	0.14	0.11	0.18	0.00	0.05	98.20%	
	Sexual abuse	20	0.16	0.11	0.20	0.01	0.09	99.50%	
	Intimate partner violence	6	0.22	0.17	0.27	0.00	0.05	97.50%	

*Note*: *k* = number of meta‐analyses.

Abbreviations: EA, emotional abuse; IPV, intimate partner violence; Neg, neglect; PA, physical abuse; SA, sexual abuse;.

We did not identify statistically significant differences in effect sizes related to different forms of CM for: externalising problems, thought problems, and substance use. Statistically significant differences were observed for three outcomes: (1) internalising problems (2) suicidal distress (3) other psychological difficulties. For internalising problems, PA had the weakest association (*r* = 0.18; 95% CI = 0.14–0.21) and EA the strongest association (*r* = 0.26; 95% CI = 0.22–0.29). For suicidal distress, neglect had the weakest effect size (*r* = 0.16; CI 95% CI = 0.12–0.20) and EA the largest (*r* = 0.26; 95% CI = 0.22–0.29). For other psychological difficulties, PA had the weakest effect size (*r* = 0.14; 95% CI = 0.11–0.18) and neglect had the largest effect (*r* = 0.25; 95% CI 0.18–0.31). Notably, there was not enough data to support subgroup analyses of the impact of specific forms of CM (e.g., IPV) on certain outcomes (e.g., thought problems). It should be noted that across the vast majority of meta‐analyses, tau‐squared was larger than 0.01 (Tables 1, 2, 3) indicating substantial dispersion of moderate meta‐analytic effect sizes.

Procedural moderator analyses (i.e., study quality, effect size conversation, estimated N, heterogeneity of variance and PB) and outlier analyses are presented in Supporting Information S1: Appendix S7. In general, these moderators and outliers did not seem to have a substantial influence on the magnitude of effect sizes. However, it is notable that there was a statistically significant difference between converted and non‐converted effect sizes for thought problems, with converted effect sizes (i.e., effect sizes that were converted to *r* from another metric) being related to significantly larger pooled effect sizes (*r* = 0.27; 95% CI = 0.22–0.31) than effect sizes already estimated as r (*r* = 0.19; 95% CI = 0.16–0.22).

## DISCUSSION

**FIGURE 2:**
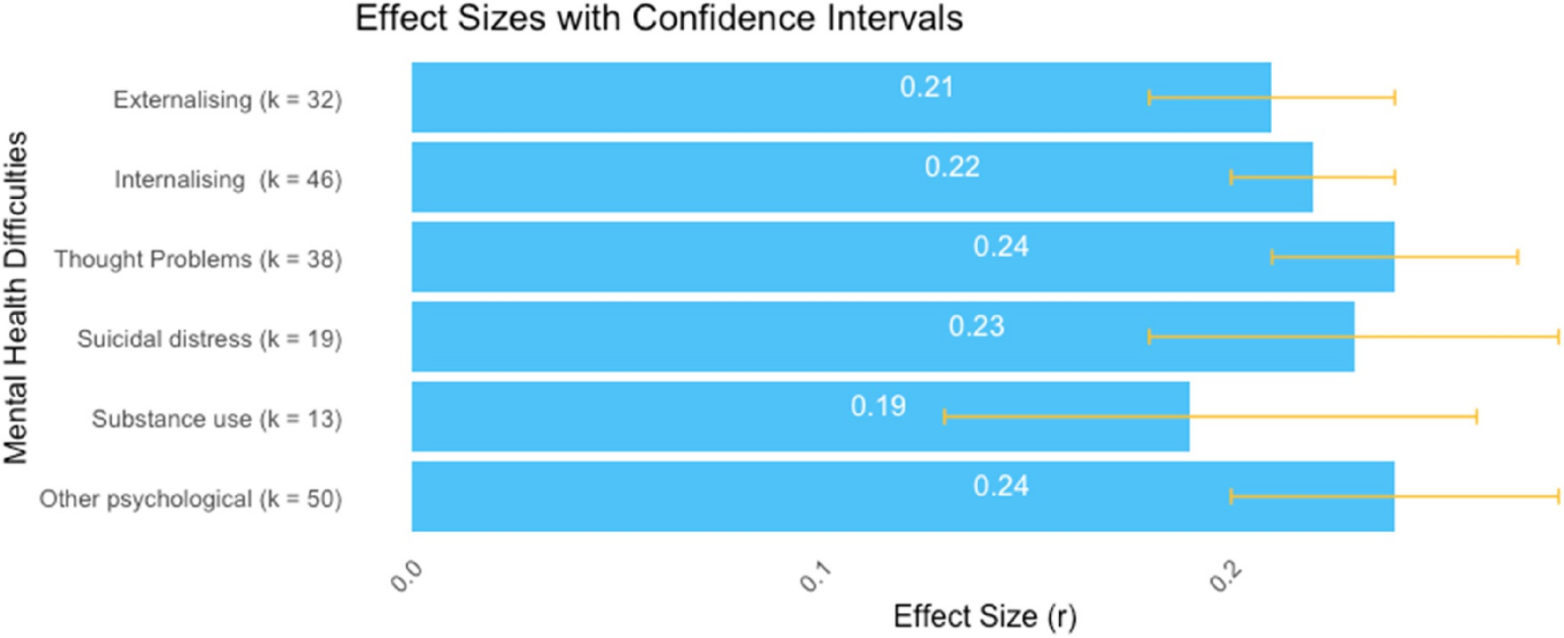
Umbrella effect sizes for the associations of child maltreatment with examined outcomes.

Drawing on data from 148 meta‐analyses, this umbrella synthesis documents that CM is significantly associated with mental health difficulties. The combined effect sizes for these associations are robust, with the smallest effect size being observed for substance misuse (*r* = 0.19) and the largest effect size for thought problems (*r* = 0.27, see Figure 2). Overall these results support previous work on the topic, documenting comparable associations to those reported in the umbrella synthesis literature (Hailes et al., 2019; Hutchens & Kearney, 2020; Kim & Royle, 2025; Sahle et al., 2021). The findings from the current umbrella synthesis extend previous umbrella syntheses by examining various forms of CM and a range of mental health difficulties. This umbrella synthesis enhances our knowledge in two key ways.

**FIGURE 3:**
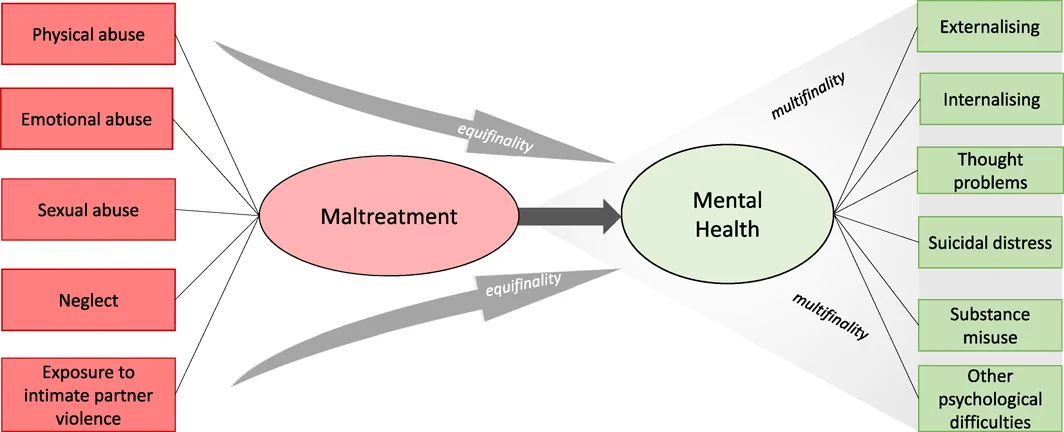
Model depicting equifinality and multifinality in maltreatment and mental health. Heuristic model depicting the associations between maltreatment and mental health, highlighting the potential for equifinality and multifinality.

First, rather than focusing on a specific outcome (e.g., internalising problems), the findings from the current synthesis show that, at least at a population level, CM is associated with a diverse range of mental health difficulties. This aligns with recent health service research on risk profiles in mental health services, which shows that adversity in its various forms is a transdiagnostic risk factor for various mental health difficulties (Coughlan et al., 2025). Our findings also align with the hypothesis of multifinality in developmental psychopathology (Cicchetti & Rogosch, 1996): the idea that a given developmental experience can contribute to an array of outcomes (Figure 3). We found, for instance, that EA is consistently associated with internalising problems, suicidal distress, and other psychological difficulties. Along similar lines, within the category other psychological difficulties we can see that CM is associated with various aspects of psychological functioning. One explanation that might be offered for our finding is that some common processes (e.g., negative affectivity or executive function problems) are involved across different mental illnesses despite their phenotypic variation.

However, an important consideration here is the role of comorbidity. It is well established in the psychiatric literature that mental health difficulties often co‐occur (e.g., Becker & Fogleman, 2020). Comorbidity has particular relevance here as it is possible that CM survivors have a heightened propensity to experience symptoms that cut across traditional nosological categories (Dvir et al., 2014). Accounting for this co‐occurrence, the general psychopathology (P) factor has been suggested, which can be understood as a general propensity to develop or experience mental health problems (Lahey et al., 2012; Neumann et al., 2016).

This would imply that traditionally separate categories of psychopathology might stem from common underlying processes. For instance, CM may lead to elevated thought problems, negative affectivity and/or lower effort control. For some children, these differences might be fertile soil to develop various and complex mental health issues. The umbrella synthesis effect sizes for the association between CM and these different mental health difficulties might support this hypothesis as these types of problems load substantially on the general P factor (Neumann et al., 2016). Although there is some longitudinal data to support this idea (Horn et al., 2018), most of the studies included in this umbrella synthesis are cross‐sectional and therefore further work is required to better understand the nature of the associations between CM and later mental health difficulties, potentially examining the moderating influence of other difficulties such as executive functioning problems.

A second key contribution is that, in general, we did not observe statistically significant differences between different forms of CM for most mental health outcomes. Because the meta‐analytic effect sizes showed heterogeneity, the identification of differences between subsets was more difficult, also in view of the modest overall number of effect sizes. Still, we did not find significant differences between different forms of CM as related to externalising problems, thought problems, and substance use. Aside from PA and some cases of neglect, associations between different forms of abuse were also comparable for internalising problems, suicidal distress, and other psychological difficulties. Furthermore, when examining associations between types of CM and any mental health difficulty, effect sizes were comparable (see Table 3). These findings may also have theoretical consequences for social learning theory which posits specific continuities between types of maltreatment and different outcomes (e.g., PA and externalising behaviour; Bandura, 1973). Internalising and thought problems may instead modulate a similar subjective experience of different objective maltreatment experiences leading to the same mental health issue later in life. This would be in line with studies showing large discrepancies between subjective and objective experiences and their developmental sequelae (Baldwin et al., 2019; Francis et al., 2023; Negriff et al., 2017).

These findings can also be interpreted through the lens of equifinality: the idea that various developmental experiences can contribute to the same outcome (Cicchetti & Rogosch, 1996; see Figure 2). For instance, all forms of CM are related to internalising problems. Such equifinality may suggest some common mechanism (e.g., affectivity) linking different forms of CM, despite their apparent diversity, to a specific mental health difficulty. An alternative explanation would be that distinct mechanisms and processes (e.g., negative affectivity, hostile attributional style, insecure attachment) play a role in the associations between different types of CM and specific mental health difficulties. This is possible, but seems a less parsimonious explanation.

If replicated, these findings might invite us to challenge conventional wisdom that suggests that some forms of CM are intrinsically more harmful to mental health than others. However, it is important to note that the effect sizes generated in this umbrella synthesis are measures of effect in a given population, which is impacted by the number of people in that population. Meta‐analytic work indicates that there may be substantial differences between the prevalence of different types of CM (Stoltenborgh et al., 2015).

Further complexity is added by the fact that this umbrella synthesis was not methodologically positioned to measure poly‐victimisation or duration of CM. Poly‐victimisation, defined as the experience of multiple types of maltreatment by the same individual, has been observed in more than half of maltreatment cases in several epidemiological studies (Euser et al., 2016; Finkelhor et al., 2009). Thus, the similarity between the associations of different forms of maltreatment may be shaped by poly‐victimisation, which was generally not addressed by the meta‐analyses considered in this umbrella synthesis. Therefore, future research should examine whether associations between forms of maltreatment and mental health remain comparable once poly‐victimisation is considered. Although at a population level maltreatment is robustly correlated with mental health difficulties, it is important to note that survivors of CM are by no means destined to experience mental ill‐health. We propose two speculative interpretations that should be tested in future research. The first interpretation is related to measurement error in the reporting of maltreatment experiences as well as the presence of mental health issues. Some ‐ although not all ‐ of what appears to be resilience following maltreatment may reflect unrecognised mental health needs. Many survivors of CM may not report such experiences. Second, survivors of CM might underreport their needs for support in coping with mental health problems arising from maltreatment. Of course, associations might also be weakened by acknowledgement of mental health issues without reporting maltreatment experiences.

From a clinical perspective, these findings offer a set of empirically grounded prior probabilities which clinicians can use to inform their clinical reasoning. Regarding intervention, Hailes et al. (2019) amongst others have emphasised the need for further work to establish whether a particular form of mental health support or intervention should be offered to survivors of CM. The findings from our umbrella synthesis suggest that the mental health trajectories of children and young people show variation. Of course, the current umbrella synthesis is not methodologically positioned to indicate which interventions would be most effective. Nevertheless, given the variation in sequelae, it seems reasonable to speculate that survivors of CM might benefit from being offered a flexible suite of assessments, mental health supports and interventions. This might involve adopting a transdiagnostic approach to service provision for survivors of CM. However, much more work is needed to uncover the pathways from maltreatment to mental health and related difficulties.

One of the main limitations is that it was not possible in this umbrella synthesis to assess how moderators such as gender, ethnicity, age at the assessment of CM and outcome, and socioeconomic status influence the association between CM and the outcomes examined. This might lead to over‐ or underestimation of effect sizes for some groups. In the absence of moderator analyses, overall combined effect sizes might not adequately represent the rather substantial dispersion of the true meta‐analytic effect sizes (Borenstein et al., 2021; see Figure 2). We regard this a critical area for further umbrella syntheses.

Furthermore, the majority of the meta‐analytic evidence reported in this umbrella synthesis is correlational and cannot control for possible pre‐existing psychiatric or genetic risk factors acting as confounders (Baldwin et al., 2023; Hailes et al., 2019; Warrier et al., 2021). This might be a recurrent challenge for the umbrella synthesis approach but increasing use of quasi‐experimental designs (Baldwin et al., 2023) or genetically informed designs (Warrier et al., 2021) might ultimately lead to umbrella syntheses with causal implications.

The methodological issue of overlapping primary studies is a material challenge of umbrella syntheses, precluding some authors from conducting quantitative synthesis of findings (e.g., Moncrieff et al., 2022). In this umbrella synthesis, we have set the threshold at 70%. Yet we acknowledge that this threshold is somewhat arbitrary. Further methodological work to establish thresholds would be useful. Another important consideration is the definition of maltreatment. We adopted a broad definition of maltreatment, but we were unable to provide an account of poly‐victimisation, severity and duration of abuse. Similarly, due to a lack of meta‐analyses, we were not able to disaggregate different types of neglect. Therefore, it remains unclear whether different forms of neglect (e.g., physical neglect, emotional neglect) are differentially associated with mental health and developmental outcomes. Furthermore, we could not differentiate between meta‐analytic results based on subjective versus objective maltreatment reports (Francis et al., 2023) and retrospective versus prospective designs (Baldwin et al., 2019) which might show diverging effect sizes. Lastly, age of reporting mental health difficulties (e.g., during adolescence, early adulthood, middle or older adulthood) may also influence effect sizes.

We also acknowledge that other ways of sorting mental health and related difficulties are possible. For example, Caspi et al. (2014) characterise substance misuse (as outcome) as an externalising disorder, whereas we regarded substance misuse as a separate outcome. Notably, the effect sizes were comparable, *r* = 0.21 and *r* = 0.19, respectively.

Finally, the most commonly used formulae for conversion between r, d and OR based on the assumption of equal sample size have the potential to introduce bias. In general, we did not identify statistically significant differences between transformed and untransformed effect sizes. However, significant differences were observed regarding thought problems.

Still, this umbrella synthesis represents the largest quantitative synthesis of the literature on CM undertaken to date. The synthesis of meta‐analyses shows that there is a consistent association between CM and various mental health difficulties. It is beyond reasonable doubt that CM is an important risk factor for mental health difficulties at a population level. Further, the associations tend to be of similar strength regardless of the mental health difficulty and the specific form of maltreatment. Taken together, these findings support the idea that there may be common mechanisms or pathways linking any CM to later mental health.

## AUTHOR CONTRIBUTIONS

**Barry Coughlan**: Conceptualization; methodology; software; formal analysis; investigation; resources; data curation; writing—original draft; visualization; project administration. **Robbie Duschinsky**: Conceptualization; methodology; writing—review and editing; supervision. **Marian J. Bakermans‐Kranenburg**: Conceptualization; methodology; writing—review and editing; visualization. **Lianne Bakkum**: Methodology; investigation; data curation; writing—review and editing. **Guy C. M. Skinner**: Methodology; investigation; data curation; writing—review and editing. **Alfred Markham**: Investigation; data curation; writing—review and editing. **Helen Beckwith**: Investigation; data curation; writing—review and editing. **Marinus H. van IJzendoorn**: Conceptualization; methodology; formal analysis; investigation; writing—review and editing; visualization; supervision.

## CONFLICT OF INTEREST STATEMENT

The authors declare no conflicts of interest.

## ETHICAL CONSIDERATIONS

This study reports an umbrella synthesis of existing meta‐analytic literature and thus ethical approval is not required.

## TRIAL REGISTRATION

A review protocol was submitted to PROSPERO on 15 July 2021 and registered on 10 August 2021 (CRD: 42021266037).

## Supporting information

Supporting Information S1

## Data Availability

Data can be found here: https://osf.io/mbj4c/?view_only=a8c3017f12fe4bd082e36ff62aad1c30/.
