# Supplementary material for: Sequelae of child maltreatment: Umbrella synthesis of 148 meta‐analyses on the mental health correlates
Source: JCPP Adv. 2026 Jan 15:e70081. Online ahead of print. doi: 10.1002/jcv2.70081 (PMC13338981; doi:10.1002/jcv2.70081)
Supplement: Supplementary file 1 — Supporting Information S1 [file JCV2-9999-e70081-s001.docx]

**Sequelae of Child Maltreatment: Umbrella synthesis of 148 meta-analysis on the mental health correlates**

**Supporting Information**

Table of Contents

[Appendix S1: Search Terms and Counts 2](#_Toc214720069)

[Appendix S2: Decision flow-chart 14](#_Toc214720070)

[Appendix S3: Quality coding form 15](#_Toc214720071)

[Appendix S4: Data and R code 16](#_Toc214720072)

[Appendix S5: Adjustment to Protocol 17](#_Toc214720073)

[Appendix S6: Additional information about included studies 18](#_Toc214720074)

[Appendix S7: Moderator and outlier analysis 57](#_Toc214720075)

[Appendix S8: Quality Rating Scores 63](#_Toc214720076)

# Appendix S1: Search Terms and Counts

Table 1: Search results 1900-July 2021

| # | Domain  [1900- 19.07.21] |  | Search term | Medline | PubMed | Embase | PsycINFO | Web of Knowledge | Cochrane | ASSIA | Dissertations & Thesis | Scopus |
| --- | --- | --- | --- | --- | --- | --- | --- | --- | --- | --- | --- | --- |
| 1 | Population |  | Child* |  |  |  |  |  |  |  |  |  |
|  |  |  | Infant* |  |  |  |  |  |  |  |  |  |
|  |  |  | Adolesc* |  |  |  |  |  |  |  |  |  |
|  |  |  | Antenatal |  |  |  |  |  |  |  |  |  |
|  |  |  | Neonat* |  |  |  |  |  |  |  |  |  |
|  |  |  | p$ediatric |  |  |  |  |  |  |  |  |  |
|  |  |  | young |  |  |  |  |  |  |  |  |  |
|  |  |  | teen |  |  |  |  |  |  |  |  |  |
|  |  |  | youth |  |  |  |  |  |  |  |  |  |
|  |  |  | baby |  |  |  |  |  |  |  |  |  |
|  |  |  | babies |  |  |  |  |  |  |  |  |  |
|  |  |  | Prenat* |  |  |  |  |  |  |  |  |  |
|  |  |  | newborn |  |  |  |  |  |  |  |  |  |
|  |  |  | boys |  |  |  |  |  |  |  |  |  |
|  |  |  | girls |  |  |  |  |  |  |  |  |  |
|  |  | # | OR | 2,772,093 | 2,860,411 | 3,431,223 | 1,109,592 | 5,515,906 | 3,262 | 2,345,366 | 422,724 | 4,395,406 |
| 2 | Exposure |  | Maltreat* |  |  |  |  |  |  |  |  |  |
|  |  |  | Abuse* |  |  |  |  |  |  |  |  |  |
|  |  |  | Neglect* |  |  |  |  |  |  |  |  |  |
|  |  |  | Family NEAR/2 violen* |  |  |  |  |  |  |  |  |  |
|  |  |  | “IPV” |  |  |  |  |  |  |  |  |  |
|  |  |  | “intimate partner violence” |  |  |  |  |  |  |  |  |  |
|  |  |  | Domestic NEAR/2 violen* |  |  |  |  |  |  |  |  |  |
|  |  |  | F$etal NEAR/2 alcohol |  |  |  |  |  |  |  |  |  |
|  |  |  | “parent* alcohol” |  |  |  |  |  |  |  |  |  |
|  |  |  | Neonatal NEAR/2 abstinence |  |  |  |  |  |  |  |  |  |
|  |  |  | “parent* substance” |  |  |  |  |  |  |  |  |  |
|  |  |  | ACE |  |  |  |  |  |  |  |  |  |
|  |  |  | "adverse child*" |  |  |  |  |  |  |  |  |  |
|  |  |  | "adversive child*" |  |  |  |  |  |  |  |  |  |
|  |  | # | OR | 256,863 | 351,399 | 336,386 | 186,919 | 570,084 | 196 | 387,889 | 77,892 | 551,677 |
| 3 | Study Type |  | Meta-analy* |  |  |  |  |  |  |  |  |  |
|  |  |  | metaanaly* |  |  |  |  |  |  |  |  |  |
|  |  |  | quantitative NEAR/2 synthesis |  |  |  |  |  |  |  |  |  |
|  |  |  | “research synthesis” |  |  |  |  |  |  |  |  |  |
|  |  |  | “quantitative review” |  |  |  |  |  |  |  |  |  |
|  |  |  | meta NEAR/2 review |  |  |  |  |  |  |  |  |  |
|  |  |  | “meta-regression” |  |  |  |  |  |  |  |  |  |
|  |  |  | meta-NEAR/2 synthesis |  |  |  |  |  |  |  |  |  |
|  |  |  | IPD |  |  |  |  |  |  |  |  |  |
|  |  |  | "Individual Participant Data" |  |  |  |  |  |  |  |  |  |
|  |  |  | "individual patient data" |  |  |  |  |  |  |  |  |  |
|  |  | # | OR | 215,826 | 215,087 | 281,140 | 45,078 | 320,654 | 3050 | 39,874 | 11.074 | 249,257 |
|  | Combined | #1 AND #2 AND #3 | | 876 | 1,123 | 1,101 | 799 | 1,291 | 47 | 810 | 142 | 1,111 |

Table 2: Search results July 2021-2023

| # | Domain  [19.07.21- 05.05.23] |  | Search term | Medline | PubMed | Embase | PsycINFO | Web of Knowledge | Cochrane | ASSIA | Dissertations & Thesis | Scopus |
| --- | --- | --- | --- | --- | --- | --- | --- | --- | --- | --- | --- | --- |
| 1 | Population |  | Child* |  |  |  |  |  |  |  |  |  |
|  |  |  | Infant* |  |  |  |  |  |  |  |  |  |
|  |  |  | Adolesc* |  |  |  |  |  |  |  |  |  |
|  |  |  | Antenatal |  |  |  |  |  |  |  |  |  |
|  |  |  | Neonat* |  |  |  |  |  |  |  |  |  |
|  |  |  | p$ediatric |  |  |  |  |  |  |  |  |  |
|  |  |  | young |  |  |  |  |  |  |  |  |  |
|  |  |  | teen |  |  |  |  |  |  |  |  |  |
|  |  |  | youth |  |  |  |  |  |  |  |  |  |
|  |  |  | baby |  |  |  |  |  |  |  |  |  |
|  |  |  | babies |  |  |  |  |  |  |  |  |  |
|  |  |  | Prenat* |  |  |  |  |  |  |  |  |  |
|  |  |  | newborn |  |  |  |  |  |  |  |  |  |
|  |  |  | boys |  |  |  |  |  |  |  |  |  |
|  |  |  | girls |  |  |  |  |  |  |  |  |  |
|  |  | # | OR | 581,739 | 2,859 | 509,347 | 108,324 | 692,303 | 313 | 28,048 | 10,123 | 331,659 |
| 2 | Exposure |  | Maltreat* |  |  |  |  |  |  |  |  |  |
|  |  |  | Abuse* |  |  |  |  |  |  |  |  |  |
|  |  |  | Neglect* |  |  |  |  |  |  |  |  |  |
|  |  |  | Family NEAR/2 violen* |  |  |  |  |  |  |  |  |  |
|  |  |  | “IPV” |  |  |  |  |  |  |  |  |  |
|  |  |  | “intimate partner violence” |  |  |  |  |  |  |  |  |  |
|  |  |  | Domestic NEAR/2 violen* |  |  |  |  |  |  |  |  |  |
|  |  |  | F$etal NEAR/2 alcohol |  |  |  |  |  |  |  |  |  |
|  |  |  | “parent* alcohol” |  |  |  |  |  |  |  |  |  |
|  |  |  | Neonatal NEAR/2 abstinence |  |  |  |  |  |  |  |  |  |
|  |  |  | “parent* substance” |  |  |  |  |  |  |  |  |  |
|  |  |  | ACE |  |  |  |  |  |  |  |  |  |
|  |  |  | "adverse child*" |  |  |  |  |  |  |  |  |  |
|  |  |  | "adversive child*" |  |  |  |  |  |  |  |  |  |
|  |  | # | OR | 50,685 | 107 | 47,054 | 24,480 | 72,666 | 11 | 9,683 | 1,753 | 48,315 |
| 3 | Study Type |  | Meta-analy* |  |  |  |  |  |  |  |  |  |
|  |  |  | metaanaly* |  |  |  |  |  |  |  |  |  |
|  |  |  | quantitative NEAR/2 synthesis |  |  |  |  |  |  |  |  |  |
|  |  |  | “research synthesis” |  |  |  |  |  |  |  |  |  |
|  |  |  | “quantitative review” |  |  |  |  |  |  |  |  |  |
|  |  |  | meta NEAR/2 review |  |  |  |  |  |  |  |  |  |
|  |  |  | “meta-regression” |  |  |  |  |  |  |  |  |  |
|  |  |  | meta-NEAR/2 synthesis |  |  |  |  |  |  |  |  |  |
|  |  |  | IPD |  |  |  |  |  |  |  |  |  |
|  |  |  | "Individual Participant Data" |  |  |  |  |  |  |  |  |  |
|  |  |  | "individual patient data" |  |  |  |  |  |  |  |  |  |
|  |  | # | OR | 85,452 | 187 | 101,571 | 9,596 | 2036 | 321 | 213 | 374 | 59,215 |
|  | Combined | #1 AND #2 AND #3 | | 529 | 12 | 366 | 298 | 381 | 503 | 62 | 20 | 275 |

Table 3: Search results Jan 2023-2024

| # | Domain  [Jan 2023-Jan 2024] |  | Search term | Medline & Embase  26.01.24 | PubMed  26.01.24 |  | PsycINF1O  25.01.24 | Web of Knowledge  25.01.24 | Cochrane  26.01.24 | ASSIA  26.01.24 | Dissertations & Thesis  26.01.24 | Scopus  25.01.24 |
| --- | --- | --- | --- | --- | --- | --- | --- | --- | --- | --- | --- | --- |
| 1 | Population |  | Child* |  |  |  |  |  |  |  |  |  |
|  |  |  | Infant* |  |  |  |  |  |  |  |  |  |
|  |  |  | Adolesc* |  |  |  |  |  |  |  |  |  |
|  |  |  | Antenatal |  |  |  |  |  |  |  |  |  |
|  |  |  | Neonat* |  |  |  |  |  |  |  |  |  |
|  |  |  | p$ediatric |  |  |  |  |  |  |  |  |  |
|  |  |  | young |  |  |  |  |  |  |  |  |  |
|  |  |  | teen |  |  |  |  |  |  |  |  |  |
|  |  |  | youth |  |  |  |  |  |  |  |  |  |
|  |  |  | baby |  |  |  |  |  |  |  |  |  |
|  |  |  | babies |  |  |  |  |  |  |  |  |  |
|  |  |  | Prenat* |  |  |  |  |  |  |  |  |  |
|  |  |  | newborn |  |  |  |  |  |  |  |  |  |
|  |  |  | boys |  |  |  |  |  |  |  |  |  |
|  |  |  | girls |  |  |  |  |  |  |  |  |  |
|  |  | # | OR | 404,718 | 185,114 |  | 36,789 | 314,629 | 209 | 10,028 | 348,577 | 261,628 |
| 2 | Exposure |  | Maltreat* |  |  |  |  |  |  |  |  |  |
|  |  |  | Abuse* |  |  |  |  |  |  |  |  |  |
|  |  |  | Neglect* |  |  |  |  |  |  |  |  |  |
|  |  |  | Family NEAR/2 violen* |  |  |  |  |  |  |  |  |  |
|  |  |  | “IPV” |  |  |  |  |  |  |  |  |  |
|  |  |  | “intimate partner violence” |  |  |  |  |  |  |  |  |  |
|  |  |  | Domestic NEAR/2 violen* |  |  |  |  |  |  |  |  |  |
|  |  |  | F$etal NEAR/2 alcohol |  |  |  |  |  |  |  |  |  |
|  |  |  | “parent* alcohol” |  |  |  |  |  |  |  |  |  |
|  |  |  | Neonatal NEAR/2 abstinence |  |  |  |  |  |  |  |  |  |
|  |  |  | “parent* substance” |  |  |  |  |  |  |  |  |  |
|  |  |  | ACE |  |  |  |  |  |  |  |  |  |
|  |  |  | "adverse child*" |  |  |  |  |  |  |  |  |  |
|  |  |  | "adversive child*" |  |  |  |  |  |  |  |  |  |
|  |  | # | OR | 6825 | 18,825 |  | 5,960 | 35,383 | 12 | 1,513 | 37,618 | 23 |
| 3 | Study Type |  | Meta-analy* |  |  |  |  |  |  |  |  |  |
|  |  |  | metaanaly* |  |  |  |  |  |  |  |  |  |
|  |  |  | quantitative NEAR/2 synthesis |  |  |  |  |  |  |  |  |  |
|  |  |  | “research synthesis” |  |  |  |  |  |  |  |  |  |
|  |  |  | “quantitative review” |  |  |  |  |  |  |  |  |  |
|  |  |  | meta NEAR/2 review |  |  |  |  |  |  |  |  |  |
|  |  |  | “meta-regression” |  |  |  |  |  |  |  |  |  |
|  |  |  | meta-NEAR/2 synthesis |  |  |  |  |  |  |  |  |  |
|  |  |  | IPD |  |  |  |  |  |  |  |  |  |
|  |  |  | "Individual Participant Data" |  |  |  |  |  |  |  |  |  |
|  |  |  | "individual patient data" |  |  |  |  |  |  |  |  |  |
|  |  | # | OR | 91,210 | 43,390 |  | 4,151 | 54,698 | 172 | 1,033 | 19,732 | 47,241 |
|  | Combined | #1 AND #2 AND #3 | | 109 | 227 |  | 123 | 244 | 60 | 22 | 246 | 2 |

# Appendix S2: Decision flow-chart


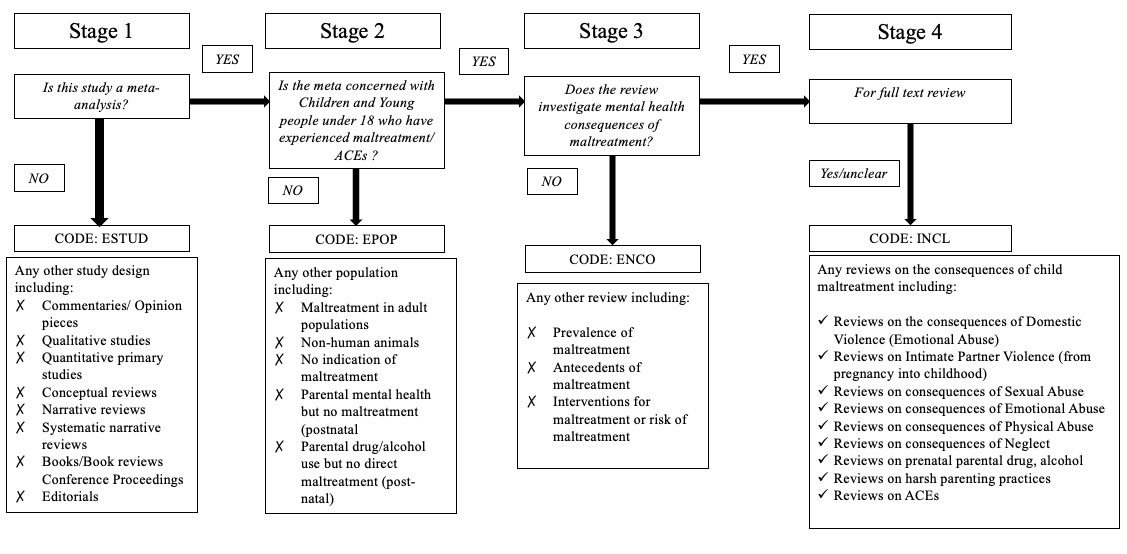


# Appendix S3: Quality coding form

Quality rating form

|  | Item | Response | Scoring |
| --- | --- | --- | --- |
| 1 | Was the search systematic? | Yes | 1 |
|  |  | No/unclear | 0 |
| 2 | Was the N for the effect size generated on 1000 people or more? | Yes | 1 |
|  |  | No | 0 |
| 3 | Was there evidence of heterogeneity? | Yes | 0 |
|  | (e.g., I^2^ >50%, significant Cochran's Q) | No/NR | 1 |
| 4 | Was there evidence of publication bias? | Yes | 0 |
|  | (e.g., significant egger's test, asymmetrical funnel plot) | No/NR | 1 |
| 5 | Was there a quality assessment? | Yes | 1 |
|  |  | No/NR | 0 |
| 6 | Was title and abstract screening completed by two or more coders independently? | Yes | 1 |
|  |  | No/NR | 0 |
| 7 | Was the review at full text completed by two or more coders independently? | Yes | 1 |
|  |  | No/NR | 0 |
| 8 | Was data extraction completed by two or more coders independently? | Yes | 1 |
|  |  | No/NR | 0 |
| 9 | Was quality assessment completed by two or more coders independently? | Yes | 1 |
|  |  | No/NR | 0 |

nr = not reported; Scores of six are higher were rated as high quality.

# Appendix S4: Data and R code

https://osf.io/mbj4c/?view_only=a8c3017f12fe4bd082e36ff62aad1c3

# Appendix S5: Adjustment to Protocol

There were three key adjustments to the preregistered protocol.

1. We were unable to explore the influence of sociodemographic and certain methodological factors (e.g., measurement of child maltreatment) on the association between maltreatment and mental health. This was due to the variation between meta-analysis regarding their reporting practices for subgroups.
2. A date restriction was added to safeguard against excessive overlap in primary studies.
3. We excluded meta-analytic associations that were based on analysis of fewer than four primary studies (cf. Bakermans–Kranenburg, Van IJzendoorn, & Juffer, 2003; Juffer & Van IJzendoorn, 2007)

To note the registered protocol also includes details about the review on physical health/neurobiological and education/social outcomes.

# Appendix S6: Additional information about included studies

| **authors** | **Type of Assessment** | ***Quotes from paper regarding definitions of child maltreatment** | ***Quotes from papers regarding definition of outcome** | **study design** |
| --- | --- | --- | --- | --- |
| Agnew-Blais 2016 | mixed | We included articles that satisfied the following criteria: definition of childhood adversities consistent with maltreatment (physical abuse, sexual abuse, emotional abuse, neglect, or family conflict) before age of 18 years; (p343) | No further information | mixed |
| Alameda 2021 | self-report | Exposure to CA measured as follows: by a (a) general measure of adversity (GA) broadly defined by a composite measure that includes either cumulative scores of different traumas, or use a binary measure that select patients having been exposed to any of the subtypes; (b) SA, (c) physical abuse (PA), (d) emotional abuse (EA), (e) physical neglect (PN), and (f) emotional neglect (EN), (g) bullying, and (h) household discord or equivalent experiences; (6) examine the positive, negative, disorganized, depressive, and manic/excited symptom dimensions13,14 and are measured using a well-validated instrument (p3) | (3) included only psychotic patients with the following diagnoses: schizophrenia, schizophreniform/ brief psychotic episode, bipolar disorder with psychotic features, schizoaffective disorder, major depression with psychotic features, and psychosis not otherwise specified; (4) defined diagnoses according to the Diagnostic and Statistical Manual of Mental Disorders (DSM-III, DSM-III-R, DSM-IV, and DSM-IV-TR17), Research Diagnostic Criteria, or International Classification of Diseases, 9th or 10th Revision (ICD-9 or ICD-10, respectively18; (p2) | mixed |
| Amado 2015 | mixed | as the involvement of a child in sexual activity that he or she does not fully comprehend, is unable to give informed consent to, or for which the child is not developmentally prepared and cannot give consent] P 50 | Defined as any internalising | Mixed |
| Angelakis 2020a | self-report | Child maltreatment including sexual, physical, emotional abuse, emotional neglect no further definition (p3) | suicide experiences, including suicide thoughts, plans, and/or attempts; (p3) | mixed |
| Angelakis 2020b | nr | reported a quantitative outcome of the association between any type of childhood maltreatment (e.g. sexual, physical, emotional abuse; physical and/or emotional neglect) experienced before the age of 18 years (p1058) | suicide attempts or any other mode of suicidal experiences, such as thoughts, plans, intentions or deaths; (p1058) | mixed |
| Angelakis 2018 | self-report | based on participants aged 18 years or older, who were exposed to childhood maltreatment such as abuse or neglect,(p3) | suicidality including suicide attempts, suicidal ideation or suicide deaths in adults exposed to childhood maltreatment (e.g. before the age of 18 years old) or reported a quantitative outcome of the association between childhood maltreatment and suicidality in adults; (p3) | mixed |
| Augsburger 2019 | nr | Studies measuring childhood trauma, defined as: (1) must have occurred before age 18 or be described as in “childhood” or “adolescence” (2) must be differentiated from adulthood trauma (3). The types of childhood trauma included in the review were: childhood sexual abuse (e.g., intercourse, sexual touching), childhood physical abuse (physical harm and punishment), childhood emotional abuse (also called psychological abuse; e.g., harsh treatment or teasing by parents), childhood physical neglect (e.g., failure to provide food, hydration, or clothing), childhood emotional neglect (e.g., unresponsive to a child’s emotional needs), bullying (e.g., repeated assault, intimation, or name-calling). Parental death and parental separation were not included. Studies were included if childhood trauma was measured in at least 90% of participants. (p1112) | Studies including clinical samples with a diagnosis of schizophrenia, schizoaffective disorder, schizophreniform, psychosis not otherwise specified, first episode psychosis, delusional disorder, depression with psychotic features, and bipolar disorder with psychotic features based on DSM-III, DSM-III-R, DSM-IV, DSM-IV-TR, DSM-5, ICD-9, ICD-10, determined by semi-structured interview or psychiatrist or psychologist evaluation. (p1112) | mixed |
| Baer 2006 | Mixed | Child protection involvement and behavioural rating scale of mother's caretaking | Strange situation procedure or modified version | nr |
| Bailey 2018 | nr | Studies measuring childhood trauma, defined as: (1) must have occurred before age 18 or be described as in “childhood” or “adolescence” (2) must be differentiated from adulthood trauma (3). The types of childhood trauma included in the review were: childhood sexual abuse (e.g., intercourse, sexual touching), childhood physical abuse (physical harm and punishment), childhood emotional abuse (also called psychological abuse; e.g., harsh treatment or teasing by parents), childhood physical neglect (e.g., failure to provide food, hydration, or clothing), childhood emotional neglect (e.g., unresponsive to a child’s emotional needs), bullying (e.g., repeated assault, intimation, or name-calling). Parental death and parental separation were not included. Studies were included if childhood trauma was measured in at least 90% of participants. (p1112) | Studies including clinical samples with a diagnosis of schizophrenia, schizoaffective disorder, schizophreniform, psychosis not otherwise specified, first episode psychosis, delusional disorder, depression with psychotic features, and bipolar disorder with psychotic features based on DSM-III, DSM-III-R, DSM-IV, DSM-IV-TR, DSM-5, ICD-9, ICD-10, determined by semi-structured interview or psychiatrist or psychologist evaluation. (p1112) | mixed |
| Baldini 2023 | self-report | Suicide behaviors were classified as suicidal ideation, suicide attempt, and suicide death. P 2 | Suicide behaviors were classified as suicidal ideation, suicide attempt, and suicide death. P 2 | mixed |
| Baldwin 2023 | mixed | Included measures of childhood maltreatment in humans, defined as any of the following experiences before age 18: physical abuse, sexual abuse, emotional abuse, physical neglect, emotional neglect, institutional neglect/ deprivation, harsh physical discipline/corporal punishment, or broader measures of victimization/adverse childhood experiences (ACEs) that included the above forms of maltreatment. As such, the primary focus of this meta-analysis was on maltreatment rather than ACEs more generally (p3-4) | Examined associations with mental health outcomes, defined as diagnoses or symptoms of internalizing, externalizing, neurodevelopmental, or thought disorders, or general psychopathology, assessed concurrent to or after the observational period for maltreatment (p4) | quasi-experimental (p4) |
| Beaumont 2018 | nr | 2) The study included as part of IPV measurement a measure of parental physical IPV. Thus, the definition of children's exposure to IPV was children residing in families in which occurrences of physical IPV were reported sometimes in addition to other forms of IPV. This definition is consistent with the definitions used in other reviews (Evans et al., 2008; Kitzmann et al., 2003; Wolfe et al., 2003). Studies that explored interparental conflict, but not parental physical IPV, were excluded. Studies examining childhood exposure to community violence, exposure to only verbal aggression or parent-sibling aggression were also excluded. Also, only witnessed IPV was included and not studies where the children were also victims of the IPV. (p26) | 3) The study examined child trauma symptoms or PTSD. Studies that either only included other child adjustment variables (e.g., internalising, externalising or physical health outcomes) or where the statistics reported meant that child trauma symptoms were included amongst other adjustment variables that could not be separately analysed were excluded. (p4) | mixed |
| Bellis 2019 | self-report | We included studies that were predominantly focused on adults and populations that were not at a known high risk of ACEs; that had sample sizes of at least 1000 people; that provided prevalence data for the number of ACEs experienced (ACE count); and that presented the odds ratio (OR), relative risk (RR), or hazard ratio (HR) for one ACE and multiple ACEs (in any ACE count categories, permitting synthesis into categories of one and at least two ACEs). To be included, studies were required to have measured at least three types of ACE. (pe519) | Life course health associated outcomes no further definition | mixed |
| Bodicker 2022 | mixed | childhood maltreatment (defined as sexual, physical or emotional abuse and emotional or physical neglect before the age of 18 years) Studies on lifetime sexual or physical abuse (including traumatic experiences in childhood and adulthood with no differentiation) were not included. (p526) | cognitive affective body image no further definition | Mixed |
| Boumpa 2022 | mixed | Childhood sexual abuse only | Binary yes/no variable of PTSD diagnosis defined by any diagnostic system ICD-11, ICD-10, DSM-III, DSMIV, and DSM-5 (p2) | mixed |
| Braga 2018 | mixed | Our definition of maltreatment was based on the WHO and the ISPCAN; specifically, actions occurring within a relationship of responsibility, trust or power, which may cause harm to the children's health, development or dignity (WHO & ISPCAN, 2006). These actions include to the intentional use of physical force (i.e., physical abuse), the child's involvement in sexual activities to which he or she is incapable of giving informed consent or that otherwise violates the law (i.e., sexual abuse), blaming, threatening, or other non-physical aggressive actions (i.e., emotional abuse), and failure to provide for the developmental needs of the child regarding health, education, emotional development, nutrition, shelter, and/or safety (i.e., neglect). (p93) | Antisocial behaviors were defined as those that violate norms and values of the society (e.g., lying, theft, and aggression). These behaviors violate others' rights and, in some cases, existing legal codes (Kazdin, 1992). We considered adult antisocial behaviors as those occurring at or after age 18, as this is a common legal age of majority. (p93) | Prospective longitudinal (p91) |
| Braga 2017 | mixed | Maltreatment was defined, according to the World Health Organization (WHO) and the International Society for Prevention of Child Abuse and Neglect (ISPCAN), as actions “resulting in actual or potential harm to the child's health, development or dignity in the context of a relationship of responsibility, trust or power” (WHO & ISPCAN, 2006, p. 9). The definition includes: physical abuse, referring to the intentional use of physical force; sexual abuse, defined as the child's involvement in sexual activities to which he or she is incapable of giving informed consent or that otherwise violates the law; emotional abuse, including blaming, threatening, or other non-physical aggressive actions; and neglect, defined as failure to provide for the developmental needs of the child regarding health, education, emotional development, nutrition, shelter, and/or safety. Other forms of adverse childhood experiences, such as exposure to intimate partner violence, were not the target of the meta-analysis. (p39) | Antisocial behaviors were defined as those that violate norms and values of the society, and include a wide variety of acts, such as lying, theft, and aggression (Kazdin, 1992). The definition includes antisocial behaviors that are criminal law violations, usually referred as delinquent or crimes, as well as acts that are not subject to criminal justice sanctions, such as externalizing or disruptive acts. Although we acknowledge the conceptual distinctions between antisocial behaviors and delinquency, these terms are used interchangeably hereafter for the purpose of simplicity. (p39) | Prospective longitudinal (p37) |
| Braithwaite 2017 | mixed | Childhood maltreatment or harsh and neglectful parenting was defined as any act or series of acts of commission (physical abuse, sexual abuse, emotional/ psychological abuse or harsh parenting) or omission (neglect) by a parent, caregiver or other person that leads to harm, the potential for harm or the threat of harm to a child (up to 18 years of age). (p2) | age). Depression was assessed either as a clinical depression diagnosis or continuous measure of depressive symptoms using scales with reported validity and reliability. The participants were children (10 years or over at outcome measure) or adults in any country and in any setting, including inpatients. (p2) | Prospective longitudinal (p2) |
| Brewin 2000 | nr | nr | DSM-III/DSM-IV | Mixed |
| Carmichael 2019 | mixed | childhood sexual (CSA), physical (CPA) and emotional abuse (CEA) and physical (CPN) or emotional neglect (CEN) no further definition (p6) | paranoia no further definition | case-control, cross-sectional or prospective cohort (p19) |
| Caslini 2016 | self-report | CSA was defined by the occurrence of at least one of the following scenarios in an individual younger than 18 years (46): 1. a sexual relationship with physical contact with a family member; 2. unwanted or forced sexual relationships with physical contact with a nonfamily adult member; and 3. a sexual relationship with a person at least 5 years older. We then defined CPA as “a continuous physical assault by a person older than 18 years which leads to identifiable pain in a subject under the age of 18” (47). Finally, CEA was defined as an act of omission and commission, which is judged based on a combination of community standards and professional expertise to be psychologically damaging. It is committed by parents or significant others who are in a position of differential power that render the child vulnerable, damaging immediately or ultimately the behavioral, cognitive, affective, social, and physiological functioning of the child (48). (p2) | We used DSM (III-R, IV) criteria for anorexia nervosa (AN), bulimia nervosa (BN), and binge eating disorder (BED). (p2) | observational studies (p2) |
| Castellvi 2017 | nr | assessed any form of IPV: (i)child maltreatment (CPA, CSA, CEA, or neglect);(ii) bullying; (iii) dating violence; or (iv) community violence (p197) | Suicide attempt was defined as any act of self-injury with intention to die, and suicide was defined as any fatal act done with the intention to take one’s own life (24). (p197) | Case controlled |
| Christy 2023 | mixed | evaluated the presence of CA, defined as having occurred before age 18 and measured as aggregated CA (here named as general adversity) or specific trauma subtype (SA, PA, EA, PN, and EN; (p287) | evaluated functional outcomes (general functioning [GF], social, or vocational functioning (p287) | mixed |
| Cri»ôan 2023 | mixed | We coded data on cumulative adverse childhood experiences, as well as specific trauma, childhood maltreatment, dysfunctional parental behaviours, household dysfunction and peer bullying as ACEs. (p1197) | cluster C personality disorders (assessed through clinical interviews or questionnaires), (p1197) | mixed |
| Croft 2021 | mixed | participants who reported exposure to trauma prior to 18 years of age and those who did not (p3) | External locus of control. A locus of control (LOC) refers to the extent to which an individual believes themselves to be accountable for their actions and is a specific dimension of attributional style [21, 22]. External attribution bias. Attribution theory refers to the way in which an individual ascribes causality to events; either to personal qualities (internal) or to others or situational factors (external) [23, 24]. Source monitoring. Source Monitoring refers to an individual’s ability to track actions and speech as produced by themselves or others and is also referred to as reality monitoring [9, 25]. Jumping to conclusions bias. A ‘jumping to conclusions’ bias refers to individuals making judgements hastily based on limited information, which can lead to reaching unwarranted conclusions [13, 26]. Top-down processing bias. An overreliance on prior expectations when perceiving new stimuli, also referred to as a greater influence of ‘top-down’ modulation in visual and auditory domains [27, 28]. This has been observed in visual and auditory domains [10, 29]. Bias against disconformity evidence. A bias against disconformity evidence (BADE) refers to a bias against revising initial probability estimations when presented with additional evidence that may contradict an individual’s initial estimation [30, 31] (p3) | mixed |
| Cui 2020 | mixed | Child maltreatment All forms of physical and/or nonphysical abuse, neglect, or negligent treatment by parents, guardians, or other family members in the household resulting in actual or potential harm to the child’s health, survival, development, or dignity. The meta-analysis focuses on its three forms: physical abuse, emotional abuse, and neglect. Physical abuse The physical force against a child by parents, guardians, or other family members in the household that results in or has a high likelihood of resulting in harm to the child’s health, survival, development, or dignity. This includes hitting, beating, kicking, shaking, biting, strangling, scalding, burning, poisoning, suffocating, and other violent acts. Emotional abuse Nonphysical forms of rejection or hostile treatment by parents, guardians, or other family members that may have a high probability of damaging the child’s physical or mental health or the child’s physical, mental, spiritual, moral, or social development, such as the restriction of movement, belittling, blaming, threatening, frightening, discriminating against, humiliating, or ridiculing. Neglect Failure to provide for the development and well-being of the child in health, education, emotional development, nutrition, shelter, or safe living conditions by parents, guardians, or other family members. | Externalizing behaviors: Acts directed to the social environment and characterized by disinhibitory control. These include aggression, delinquency/antisocial behavior, and hyperactivity/attention deficit. Aggression: Physical or verbal behaviors that harm or threaten to harm others including children, adults, and animals. Delinquency/antisocial behaviorActs (not including violent acts) that break rules or laws such as lying, cheating, stealing, and committing antisocial acts with bad companions. Hyperactivity/attention deficitAn excess of motor activity, restlessness, or attention deficits in which the children are unable to sustain and modulate their attention in a controlled setting such as the classroom. Internalizing behaviors: Negative behaviors directed inward to children themselves. These include anxiety, depression, somatic complaints, and suicide (attempts, ideation, or nonsuicidal self-injury behavior). Anxiety: Symptoms of worry, nervousness, and apprehension without cause. Depression: Symptoms characterized by excessive sadness and loss of interest in usually enjoyable activities. Somatic complaints: Physical symptoms with no identifiable, specific physiological cause. Suicide: The act (suicidal attempt) and thoughts (suicidal ideation) of intentionally causing one’s own death or the act of deliberately harming the surface of one’s own body without the intention of causing death (self-injury). | mixed |
| Cyr 2010 | mixed | "Children included in the 10 maltreatment studies were either physically abused, sexually abused, neglected, emotionally maltreated, or had experienced multiple forms of maltreatment. The most widely accepted definitions of types of maltreatment have been described in Cicchetti and Valentino (2006). Based on these descriptions, we defined (a) sexual abuse as sexual contact or attempted sexual contact between a caregiver or other responsible adult and a child, (b) physical abuse as injuries inflicted by an adult on a child by nonaccidental means, (c) neglect as the failure to provide minimum standards of physical care, and (d) emotional maltreatment as the persistent and extreme refusal to consider a child’s basic emotional needs (e.g., belittling, intimidating, severe indifference)." P91 | strange situation or attachment q sort | nr |
| De 2021 | mixed | Childhood physical or sexual abuse no further definition (p3) | Cannabis use measure (lifetime, past year, frequency) no further definition (p3) | mixed |
| De 2022 | mixed | CM as defined by the World Health Organization (WHO): Child maltreatment is the abuse and neglect that occurs to children under 18 years of age. It includes all types of physical and/or emotional ill-treatment, sexual abuse, neglect, negligence and commercial or other exploitation, which results in actual or potential harm to the child’s health, survival, development or dignity in the context of a relationship of responsibility, trust or power. Exposure to intimate partner violence is also sometimes included as a form of child maltreatment [60]. (p6) | ) psychopathy measured by means of self-report or clinician rated scales. (p6) | mixed |
| Ditzer 2023 | self-report | Child maltreatment has been acknowledged as a global public health and social welfare risk (Carr et al., 2020; Finkelhor et al., 2015; Gilbert et al., 2009; Norman et al., 2012; Peterson et al., 2018; Wegman & Stetler, 2009). It includes active (i.e., abuse) and passive (i.e., neglect) forms, as well as physical, emotional, and sexual dimensions. Due to the lack of social, cultural, and legal consensus over what child-rearing practices are harmful or unacceptable (Barnett et al., 1993; Herrenkohl, 2005; Manly, 2005), there has been considerable debate about how to define child maltreatment. While various classifications exist, a distinction between emotional, physical, and sexual abuse as well as emotional and physical neglect is widely accepted (Herrenkohl, 2005; Manly, 2005). For the purpose of the present meta-analysis, we thus adopted this classification.P 312 | alexithymia literally means “no words for feelings.” It is defined by (a) difficulty identifying and describing feelings; (b) difficulty differentiating between emotional states and physiological sensations; and (c) a concrete, externally oriented cognitive style (Bermond et al., 2007; Preece et al., 2017; 2020; Watters et al., 2016). | retrospective |
| Dolan 2018 | self-report | quantitative measurement of childhood sexual abuse. no further definition (p24) | Participants had a diagnosis of a psychosis spectrum disorder or were members of the general population. (p24) | mixed |
| Downing 2022 | mixed | Childhood maltreatment was measured retrospectively, with the main outcome measures being the Childhood Trauma Questionnaire-Short Form (CTQ-SF; Bernstein et al., 2003). Other measures included the Child Abuse and Trauma Scale (CAT; Sanders & BeckerLausen, 1995), the Traumatic Life Events Questionnaire (TLEQ; Kubany et al., 2000a) and the Invalidating Childhood Environments Scale (ICES; Mountford, Corstophine, Tomlinson, & Waller, 2007) (p7) | Outcome measures for self-compassion were the SCS and SCS-SF. (p7) | mixed |
| Duarte 2020 | self-report | included CM assessment with the CTQ (p559) | A suicide attempt was defined as any act carried out with a certain intent to die, distinct from non-suicidal self-injury (p559) | observational |
| Mandelli 2015 | Mixed | Definitions provided in Table 3 | The presence of depression in adulthood assessed by means of clinical or structured interview or self-report questionnaires. The most frequently employed method to diagnose depression was on the basis of the Diagnostic and Statistical Manual criteria (DSM-IIIR, DMS-IV, DSM-IV-TR) P 668 | Mixed |
| Evans 2008 | nr | "Different terms have been used to describe children who have been exposed to domestic violence. Early research often described children as being a “witness” or “observer” of such violence; more recently, however, researchers have begun to use the term “exposure” to domestic violence (Holden, 1998). Within the empirical literature, however, few studies articulate what is meant by “childhood exposure” and many do not report information about the type or extent of violence to which the child is exposed. Thus, to date, no standardized definition of childhood exposure to violence has emerged (Mohr, Lutz, Fantuzzo, & Perry, 2000). Despite such lack of consensus, most researchers agree that exposure to domestic violence occurs when children see, hear, are directly involved in (i.e., attempt to intervene), or experience the aftermath of physical or sexual assaults that occur between their caregivers (Edleson, 1999; Jouriles, McDonald, Norwood, & Ezell, 2001; Wolak & Finkelhor, 1998). Additionally, while much of the research surrounding childhood exposure to domestic violence has focused on male-perpetrated violence (Wolak & Finkelhor, 1998), researchers studying family violence must recognize that children may also be exposed to violence in which their mother is the perpetrator or to bidirectional acts of violence between caregivers." P 132 | "The distinguishing feature for inclusion in the meta-analysis was that the study examined the relationship between exposure to physical violence between intimate partners and child psychosocial outcomes, specifically child internalizing and externalizing problems, and trauma symptoms. Thus, studies examining childhood exposure to community violence, exposure to only verbal aggression or parent–sibling aggression were excluded. Moreover, studies were only included if child internalizing and externalizing problems were measured using a standardized instrument of known reliability and validity. P 134" | Mixed |
| Fitton 2017 | mixed | CT occurred, and was recorded/measured before 18 years old p21 | VO were measured as violent acts, rather than aggression, violent thoughts or verbal violence. p21 | prospective |
| Fossati 1999 | mixed | nr | DSM-III/ Gunderson/ Mixed | Mixed |
| Francis 2023 | self-report | Subjective measures were defined as an individual’s perception of their own adverse childhood experiences, captured through self-reported interviews or questionnaires. These measures assessed whether an event occurred (e.g. maltreatment) rather than its subjective impact. Objective measures were defined as assessments unlikely to be affected by the target individual’s perception of their experience, such as (a) official records (e.g. child protection records, crime records or medical records) or (b) reports derived from multiple individuals who are not directly related to the individual (e.g. peer nominations for bullying) p1186 | Psychopathology was defined as diagnoses or symptoms of a psychiatric illness p1186 | mixed |
| Gao 2023a | self-report | No further definition | No further definition | cross-sectional or longitudinal |
| Gao 2023b | mixed | No further definition | No further definition | cross-sectional or longitudinal |
| Gardner 2019 | mixed | child maltreatment was defined as any form of sexual abuse, physical abuse, emotional abuse, neglect, or exposure to IPV experienced before the age of 18 years. Sexual abuse included any non-contact or contact abuse such as indecent exposure, molestation, and rape (Felitti et al., 1998). Physical abuse included being hit, beaten, kicked, shook, bit, strangled, scalded, burned, poisoned or suffocated (World Health Organization & International Society for Prevention of Child Abuse & Neglect, 2006). Emotional abuse included being verbally abused, threatened, ridiculed or humiliated (Chapman et al., 2004; Paquette, Tourigny, Baril, Joly, & Seguin, 2017). Neglect included a caregiver failing to provide adequate food, shelter, love, or support to a child in their care (Krug et al., 2002). Exposure to IPV included witnessing any form of abuse (sexual, physical, emotional, or verbal) between parents or caregivers (Leeb, Paulozzi, Melanson, Simon, & Arias, 2008). In addition to the five specific forms of child maltreatment, studies defining exposure as ‘any child maltreatment’ or reporting multiple forms of child maltreatment as a single estimate were included in an ‘any child maltreatment’ exposure group. (p2) | Depressive disorders included both major depressive disorder (MDD) and dysthymia and were defined according to the Diagnostic and Statistical Manual of Mental Disorders (DSM-IV) (American Psychiatric Association, 2000) and International Classification of Disease (ICD) criteria (World Health Organisation, 1993b). Anxiety disorders included all those defined according to DSM-IV (American Psychiatric Association, 2000) and ICD criteria, (World Health Organisation, 1993a) including; generalised anxiety disorder (GAD), panic disorder, phobic disorders (agoraphobia, social and specific phobias), obsessive-compulsive disorder (OCD), post-traumatic stress (PTSD) and acute stress disorders, and anxiety not otherwise specified (NOS) (Baxter, Vos Scott, Ferrari, & Whiteford, 2014). (p2) | mixed |
| Gibb 2002 | Mixed | "Of the studies providing operational definitions of childhood sexual maltreatment, three included both unwanted contact and noncontact sexual experiences (Gibb et al., 2001a,b; Pritt, 1998); the other four included only unwanted contact sexual experiences (Hundley, 1992; Mannarino & Cohen, 1996; McCord, 1985; Wenninger & Ehlers, 1998)." P232 | In the depression literature, theorists (e.g., Beck, 1987; Clark, Beck, & Alford, 1999; Rose & Abramson, 1992) have suggested that the link between negative events in childhood and later depression may be mediated by individuals’ characteristic ways of interpreting the negative events in their lives (i.e., cognitive styles). P224 | Mixed |
| Godbout 2017 | mixed | five forms of CM were targeted: sexual abuse, physical abuse, psychological abuse, neglect, and witnessing interparental violence. | type of IPV (perpetration or victimization), and form of IPV (sexual, physical, and psychological). no further definition | mixed |
| Goncy 2021 | mixed | Parents directing aggressive acts toward their child was defined as parent-to-child aggression. | DA outcome variables (i.e., victimization, perpetration), and forms (i.e., physical, psychological) were used to identify and extract information for effect size computation. Perpetration refers to acts of aggression toward one’s partner, whereas victimization refers to receipt of aggression by one’s partner. | mixed |
| Green 2017 | nr | Experience of childhood maltreatment, including physical or sexual abuse or any form of neglect, identified by (retrospective) self-report, informant report, or official records. | Violence to others, defined as acts of physical aggression directed toward other people, property, or animals, identified by self-report, informant report, formal records, or criminal sanction. Self-harm and suicide were not the focus of this review, as distinct risk factors may be implicated in the aetiology of self-injurious behavior. | mixed |
| Gruhn 2020 | nr | Experience of childhood maltreatment, including physical or sexual abuse or any form of neglect, identified by (retrospective) self-report, informant report, or official records. | Violence to others, defined as acts of physical aggression directed toward other people, property, or animals, identified by self-report, informant report, formal records, or criminal sanction. Self-harm and suicide were not the focus of this review, as distinct risk factors may be implicated in the aetiology of self-injurious behavior. | mixed |
| Halpern 2018 | self-report | We considered the following stressful events: CM, PA, SA, neglect and interparental violence. (p3) | had illicit drug abuse or dependence as a primary outcome no further definition (p3) | mixed |
| Heerde 2019 | nr | Family risk factors Perpetration and victimization (all outcomes) Risk of violent victimization Witnessed violence and/or victimization Physical violence victimization Perpetration of physically violent behavior (p496) | The definitions of physically violent behavior used in this study are informed by the World Health Organization’s definition of violence (World Health Organization, 2002) p493 | mixed |
| Hughes 2017 | nr | using a cumulative measure of at least four ACEs spanning both direct (e.g., maltreatment) and indirect (e.g., household dysfunction) types (p357) | Depression: depression (4, L; 2, C), depressed affect (1, L), major depression (1, 5 years), moderate or severe depression (1, C), depressive symptoms (1, C), frequent depressive symptoms (1, C), depressive disorders (1, 1 year), mood disorder (1, 1 year) Illicit drug use: drug use (1, L; 1, NR); illicit drug use (1, O;† 1, NR); street drug use (7, L); cannabis use (4, L), frequent cannabis use (1, C) 16 10 11 42816 1,7,8,9,13,24,30,33–35 Problematic alcohol use: alcoholism (1, L); problem drinker or alcoholic (6, L); self-reported alcoholic (1, L); alcohol disorders (1, 1 year; 1, L) Violence victimisation: been hit (4, 1 year); physical dating violence younger than 21 years (1); sexual violence (1, adulthood) 6 6 2 25 119 9,25,27,33–35 Violence perpetration: intimate partner violence (2, L); hit someone (4, 1 year); physical dating violence younger than 21 years (1); child neglect (1, C) (pe362) | mixed |
| Humphreys 2020 | self-report | dimensional measurement of child maltreatment using the CTQ (either the long or short form) p2 | dichotomous or dimensional assessment of depression; p6 | mixed |
| Infurna 2016 | self-report | dimensional measurement of child maltreatment using the CTQ (either the long or short form) p2 | dichotomous or dimensional assessment of depression; p7 | mixed |
| Ip 2015 | Mixed | In this review, CPA was defined as ‘‘the intentional use of physical force against a child that results in, or has the potential to result in, physical injury’’ (Centers for Disease Control and Prevention, 2008, p. 14), for example, hitting, kicking, punching, beating, and stabbing (Gilbert et al., 2009). P 574 | SYMPTOM AND DIAGNOSIS (574) | nr |
| Islas-Preciado 2021 | self-report | no further definition | no further definition | case-control |
| Jumper 1995 | Mixed | contact, non-contact and consensual P720 | For the purposes of this investigation, the term psychological symptomatology refers to those psychological difficulties experienced by individuals other than depression or impaired self-esteem, such as anxiety-related problems, personality disorders, suicidal behavior, and psychiatric illnesses, including psychotic, somatoform, and dissociative disorders. P 716 | mixed |
| Kane 2017 | mixed | oincludeameasureorindexofchildmaltreatmentthatinvolvedoneofthefourmajorformsoutlinedearlier(i.e.,physicalabuse,sexualabuse,emotionalabuse,and/orneglect). p874 | interpersonal dependency or DP no further definition | mixed |
| Kautz-Turnbull 2021 | self-report | study population included individuals with FASD or con-firmed PAE and at least one control group of nonexposed individuals or individuals with ADHD. (p2432) | measuring adaptive functioning using one of the following measures: Vineland Scales of Adaptive Behavior (Sparrow et al., 1984), SIB (Bruininks et al., 1985), ABAS (Harrison & Oakland, 2015), or Behavior Assessment for Children (BASC; Merenda, 1996) (p2432) | mixed |
| Khaleque 2015 | self-report | Parental Acceptance–Rejection Questionnaire for Mothers and for Fathers P1419 | child version of the Personality Assessment Questionnaire P1419 | cross-sectional |
| Khan 2022 | mixed | childhood maltreatment (including specific types of mal-treatment such as emotional, sexual, or physical abuse or emotional or physical neglect (p964) | alexithymia no further definition | mixed |
| Kim 2021 | nr | Exposure to family violence Exposure to family violence, mother/father IPV perpetration, physical interparental violence, Neglect(Child) neglect, emotional/physical neglect from mother/father Physical abuse(Child) physical abuse/maltreatment, physical abuse from mother/father Psychological abuse(Child) psychological/emotional abuse/maltreatment, psychological abuse from mother/father Sexual abuse(Child) sexual abuse (p66) | Perceived attachment with parents is defined in this study as perceived childhood attachment styles(secure, anxiety-related, avoidance-related) with each parent or both parents. p68 | mixed |
| Kitzmann 2003 | Mixed | "Several terms need to be defined for purposes of this review. First, the term domestic violence has been used to refer to a wide range of behaviors including physical, sexual, and psychological abuse, shown by both adults and children in the context of family life. In the current article, we use the term domestic violence more specifically to refer to incidents of physical aggression (including slapping, pushing, punching, kicking, choking) between adults or parent figures in the family. We use the terms interparental violence and marital violence synonymously, although these specific terms do not apply to all families." P 340 | Decisions about categorization were guided by the factor structure of the widely used Child Behavior Checklist (CBCL; Achenbach & Edelbrock, 1991). For example, any measure of somatic complaints was classified as “internalizing” because somatic complaints are part of the internalizing factor on the CBCL, whereas any measure of attention problems was classified as “other psychological problems” because attention problems are part of the “other psychological problems” factor on the CBCL P343 | Mixed |
| Lai 2023 | self-report | participants experienced CA before the age of 18. | depression was assessed using established objective measures or diagnostic interviews, p3 | mixed |
| Lavi 2019 | mixed | Most of the studies included in the meta-analysis used official records of maltreatment or observational data taken by child welfare professionals asan indication of past maltreatment, either as an indication that the child had been maltreated or asan indication that the parent was maltreating. (p1507) | we considered a range of variables relevant to the categories of emotion reactivity and regulation | mixed |
| Lee 2021 | mixed | only two measures, namely, the Invalidating Childhood Environments Scale (ICES; Mountford et al., 2007) and the Coping with Children’s Negative Emotions Scale (CCNES; Fabes et al., 1990), were found to closely measure or align with all four components of invalidating environment (inaccuracy, misattribution, discouragement of negative emotions, and oversimplification of problem-solving) as defined by Linehan (1993). Hence, in this review, studies that utilized the ICES (Mountford et al., 2007) and the CCNES (Fabes et al., 1990) were included (p574) | BPD symptoms no further definition | mixed |
| Leiva-Bianchi 2023 | nr | Different forms of abuse (no further definition) | consistent evidence supporting the construct validity of the ICD-11 for PTSD and CPTSD has accumulated in recent years (Brewin, 2020; Brewin et al., 2017). Mainly using the International Trauma Questionnaire (ITQ; Cloitre et al., 2015; Cloitre et al., 2018) self-report instrument for the evaluation of the diagnoses of PTSD and CPTSD according to the ICD-11 (World Health Organization, 2018) (ITQ versions; https://www.traumameasuresglobal.com/itq). In clinical populations (Bottche et al., 2018; Choi et al., 2021; Dhingra et al., 2015; Dorahy et al., 2009; Folke et al., 2021; Kazlauskas et al., 2018) and general (nonclinical) population exposed to trauma (Frost et al., 2019a; Cloitre et al., 2013; Camden et al., 2023; Frewen et al., 2023) studies that confirm the six dimensions of CPTSD, findings consistent with the ICD11 definition (World Health Organization, 2018). P 342 | cohort/cross-sectional |
| LeMoult 2020 | mixed | dichotomous or continuous measurement of ELS (p843) | MDD assessment prior to a mean age of 18 years (p843) | mixed |
| Li 2020a | mixed | child sexual assault no further definition | For depression, studies on depressive disorder, major depressive disorder and dysthymic disorder were included. | mixed |
| Li 2016 | Mixed | Various types of abuse (see Table 1; P 721) | "use clear diagnosis criteria for depression or anxiety in adulthood, specifically DSM and its updates (APA, 2013), ICD-10 (WHO, 1992) or other generally accepted diagnostic criteria" P719 | prospective |
| Li 2022 | mixed | child maltreatment no further definition | had clear diagnostic criteria for depression or depressive symptom, specifically Diagnostic and Statistical Manual (DSM) and its updates, International Classification of Diseases (ICD) or other generally accepted criteria; | observational study designs, including case-control, cohort, and cross-sectional studies; |
| Li 2020b | self-report | Childhood maltreatment, defined as violent acts including physical abuse, psychological abuse, sexual abuse, and neglect against children (p2) | Intimate partner violence (IPV) perpetration, defined as the behavior within an intimate relationship that causes physical, sexual, or psychological harm (p2) | mixed |
| Lindert 2014 | self-report | nr | validated scales or clinical diagnoses | mixed (all but on cross-sectional) |
| Liu 2017 | self-report | use of the CTQ to evaluate childhood maltreatment (p148) | Suicidal behavior includes suicidal ideation (thoughts and plans of ending one's life), suicide attempt (engagement in potentially self-injurious behavior that does not result in death), and completed suicide (ending one's life) (p147) | mixed |
| Liu 2023b | self-report | The study specified an assessment of ACEs (i.e., broadly defined as any stressful and traumatic events that occurred while growing up or before the age of 18) using self-report, other-report or official records. (p3) | The study specified an assessment procedure for and nature of any sleep disturbance using self-report, other-report or official records. p3 | mixed |
| Liu 2023a | mixed | studies in which individuals experienced maltreatment/abuse/ neglect or at least one maltreatment subtype during childhood under 18 years of age and | social anxiety no further definition | mixed |
| Liu 2018 | self-report | assessed any form of childhood maltreatment, distinct from other constructs (e.g., other adverse childhood experiences) p52 | assessed non-suicidal self-injury separately from other constructs p52 | mixed |
| Longobardi 2022 | self-report | assessed any form of childhood maltreatment, distinct from other constructs (e.g., other adverse childhood experiences) p52 | assessed non-suicidal self-injury separately from other constructs p52 | mixed |
| Lucia 2020 | self-report | Studies examining exposure to all types of violence perpetrated by adults during childhood were included. | The outcome measures included the use or abuse of cannabis during adolescence, and sex differences were analysed where possible. | mixed |
| Luke 2013 | nr | nr | Social understanding is conceptualised here as the ability to understand feelings, beliefs and desires and their role in social behaviour, an ability often termed ‘theory of mind’ (Premack & Woodruff, 1978), ‘perspective taking’ or ‘mentalising’ (Carpendale & Lewis, 2006). It includes what has been called ‘emotion knowledge’: the ability to recognise specific emotional expressions in others and to understand the type of situations that can give rise to particular emotions (Sullivan, Bennett, Carpenter, & Lewis, 2008). This kind of awareness and understanding of others’ emotions may also be conceptualised as one important component of empathy. However, it has a distinctively cognitive dimension, in comparison with other aspects of empathy which are principally affective (‘‘feeling what another person is feeling’’) or behavioural (‘‘responding compassionately to another person’s distress’’; Levenson & Ruef, 1992, p. 234). P 2 | mixed (mainly cross-sectional) |
| MacMillan 1999 | self-report | "Most studies (70%) defined sexual experiences to be CSA if a sizable age discrepancy existed between the child or adolescent and other person, regardless of the younger person's willingness to participate; 20% of the studies restricted their definition of CSA to unwanted sexual experiences only. Most studies (73%) defined CSA to include both contact and noncontact (e.g., exhibitionism) sexual experiences; 24% restricted their definition to contact experiences only." P31 | 3. Depression-based on the Depression subscales of the SCL-90R, the HSCL, the BSI, the TSC-33 and 40, the MMPI form R, the Hugo Short Form of the MMPT (HSF; Hugo, 1971), and the MCMI; depression-related items from the Clinical Analysis Questionnaire (CAQ: Cattell, 1973); the Beck Depression Inventory (BDI; Beck, Ward, Mendelson, Mock, & Erbaugh, 1961); and investigator-authored items. P28 | mixed |
| Magalhaes 2023 | mixed | Child maltreatment involves acts (i.e., abuse) and/or omissions (i.e., neglect) which cause or has the potential to cause harm to children (McCoy & Keen, 2013). Different subtypes of child maltreatment have been described in the literature, namely a) physical abuse (i.e., nonaccidental physically punitive actions, such as hitting or kicking); b) emotional/psychological abuse (i.e., threatening, insulting, or humiliating the child); c) sexual abuse (i.e., sexual contact (or attempt) aimed at the sexual gratification of another person (adult or other child) and where true consent must be absent); d) physical neglect (i.e., lack of provision basic care such as hygiene or food and lack of supervision); and e) emotional neglect (i.e., emotional deprivation, absence of a secure and responsive environment, absence of responsiveness to emotional needs) (Barnett et al., 1993; Mathews & Collin-Vezina, 2019; Starr et al., 1990). P 93 | Internalizing symptoms comprises problems related to internal difficulties such as anxiety, depression, isolation, or somatic complaints. P 90 | mixed |
| Martijn 2020 | nr | Childhood maltreatment indicators, specifically those related to childhood sexual abuse, were included no further definition p531 | sexual offending no further definition | mixed |
| Matheson 2013 | Mixed | Childhood adversity covers a range of potentially harmful experiences, including emotional or psychological abuse, physical abuse, sexual abuse, neglect and other negative life events. P 225 | diagnosis | Case-control and cross-sectional |
| McIntosh 2019 | self-report | mothers’ experience of IPV victimization in the anteor perinatal periods and subsequent mother–offspring attachment security in early childhood p888 | subsequent mother–offspring attachment security in early childhood (i.e., 5 years and under p888 | mixed |
| McKay 2021 | mixed | We defined trauma based on the 10 sub-categories of child-hood trauma used by the U.S. Centres for Disease Control & Prevention (CDC) Adverse Childhood Experiences Study (‘Centre for Disease Control and Prevention’).15 These are emotional, physical and sexual abuse; household partner violence (domestic violence), household substance abuse, mental illness in household (maternal mental illness, pater-nal mental illness and sibling mental illness), loss of a par-ent (parental death, separation or divorce) or incarceration of a parent; and either emotional or physical neglect. p191 | mental disorder outcomes included both general terms (e.g. anxiety disorder, mood disorder, major de-pressive disorder, bipolar disorder and psychotic disorder) and specific terms for all DSM-V sub-categories of mental disorder p191 | mixed |
| Mitiku 2024 | nr | nr | ADHD/Depression/ emotional or behavioural problems ( see P 171) | mixed |
| Molendijk 2017 | self-report | CM (before the age of 18 years). We largely followed the definition and categorization of the WHO in what we considered as exposure to CM. The WHO (2016b) distinguishes five subtypes of CM: physical CM, sexual CM, neglect, emotional CM and exploitation. However, given that neglect and emotional abuse are often reported as one entity we decided to pool these categories as ‘emotional CM’. Furthermore, since exploitation is hardly a topic in the ED literature, we decided to not include this category in our meta-analysis p1403 | presence of a current or lifetime ED as defined by the DSM p1403 | mixed |
| Mootz 2022 | mixed | Being abused as a child no further definition | IPV perpetration no further definition | mixed |
| Muluneh 2021 | mixed | history of child and family abuse p1 | Gender-based violence was measured using the Demographic and Health survey (DHS) tool. p3 | mixed |
| Nelson 2017 | nr | study includes an assessment of childhood maltreatment defined as sexual, physical or emotional abuse, and/or physical or emotional neglect up to age 18 p1 | study includes an assessment of a diagnosis of depressive disorder or severity of depressive symptoms p1 | mixed |
| Neumann 1996 | Mixed | Most studies examined in the meta-analysis defined CSA as involving physical contact of a sexual nature, ranging from fondling to intercourse (n 26). Six included noncontact sexual abuse (e.g., forced observation of nudity; Roland, Zelhart, & Dubes, 1989). The remaining five studies did not specify contact or noncontact. P 8 | Results were based on empirical measures (as opposed to clinical impressions). P 4 | Mixed |
| Ng 2019 | self-report | childhood sexual abuse no further definition | qualitative or quantitative measure of suicidal behaviour. The primary outcome measure of interest was the incidence of suicidal attempts in both exposed (history of childhood sexual abuse) and control groups. p133 | mixed |
| Nilsen 2020 | self-report | childhood sexual abuse no further definition | qualitative or quantitative measure of suicidal behaviour. The primary outcome measure of interest was the incidence of suicidal attempts in both exposed (history of childhood sexual abuse) and control groups. p133 | mixed |
| Noonan 2020 | Mixed | included a measure of physical, sexual, p3 | To measure attachment, studies commonly used a validated self-report measure (n = 6) such as the Inventory of Parent and Peer Attachment (IPPA; Armsden & Greenberg, 1987), or a validated objective measures (n = 6), most commonly the Attachment Q-Set (AQS; Waters, 1987). p5 | mixed |
| Norman 2013 | Mixed | Child maltreatment is defined as all forms of physical and/or emotional ill-treatment, sexual abuse, neglect or negligent treatment, or commercial or other exploitation of children that results in actual or potential harm to a child's health, survival, development, or dignity in the context of a relationship of responsibility, trust, or power [1]. Four types of maltreatment are commonly recognised: sexual abuse, physical abuse, emotional abuse (also referred to as psychological abuse), and neglect (Table 1). Child maltreatment is defined as all forms of physical and/or emotional ill-treatment, sexual abuse, neglect or negligent treatment, or commercial or other exploitation of children that results in actual or potential harm to a child’s health, survival, development, or dignity in the context of a relationship of responsibility, trust, or power [1]. Four types of maltreatment are commonly recognised: sexual abuse, physical abuse, emotional abuse (also referred to as psychological abuse), and neglect (Table 1). P 4 | self-report and diagnostic criteria | Mixed |
| Ou 2021 | self-report | CM should be defined as the exposure to CPA, CEA, CSA, CPN, and CEN before 18 years old; p2 | OCD DSM diagnostic criteria, measured by YBOCS p4 | mixed |
| Palmier-Claus 2016 | Mixed | We defined childhood adversity as the experience of neglect, abuse, bullying or the loss of parents before the age of 19 years. Studies exploring loss through separation (e.g. divorce of parents), expressed emotion and/or stressful life events occurring in adulthood (after the age of 18 years) were not included | formal diagnosis of bipolar disorder according to the DSM (DSM-III or later) or ICD (ICD-9 or -10) p455 | mixed |
| Paolucci 2001 | Mixed | A standard definition of child sexual abuse has not yet been reached (Genuis, 1991; Kassim & Kassim, 1995; Violato & Genuis, 1993, 1994; Violato & Travis, 1995). Although some researchers have restricted their definition of CSA exclusively to acts of sexual intercourse and penetration, others have included a broader range of victimization, such as the witnessing of a sexual act between others, being fondled, or being spoken to in a sexual manner (Metcalfe, Oppenheimer, Dignon, & Palmer, 1990). For the present meta-analysis, we have defined CSA as any unwanted sexual contact (ranging from genital touching and fondling to penetration) during the period in which the victim is considered a child by legal definition and the perpetrator is in a position of relative power vis à vis the victim (Violato & Genuis, 1993, p. 37). P 21 | DSM-IV | Mixed |
| Pastore 2020 | Mixed | We defined childhood adversity as the experience of neglect, abuse, bullying or the loss of parents before the age of 19 years. Studies exploring loss through separation (e.g. divorce of parents), expressed emotion and/or stressful life events occurring in adulthood (after the age of 18 years) were not included | formal diagnosis of bipolar disorder according to the DSM (DSM-III or later) or ICD (ICD-9 or -10) p455 | mixed |
| Peh 2018 | nr | All studies must have recruited individuals at high risk of psychosis, as determined by the following criteria: (i) CHR, as assessed by SIPS (Miller et al., 2002), or (ii) UHR, as assessed by the CAARMS (Yung et al., 2005). p1090 | Types of childhood adversities include (i) childhood trauma, (ii) bullying victimisation and (iii) parental separation or loss. p1090 | mixed |
| Petruccelli 2019 | self-report | The CDC-Kaiser ACE scale is a distinct scale used to measure these outcomes. The term ACEs originally referred to the following types of events: physical abuse, verbal abuse, sexual abuse, and household dysfunction such as domestic violence, household substance abuse, mental illness, and criminal activity. p2 | Health outcome was broadly defined as any medical or psychosocial outcome, acknowledging that psychosocial outcomes can be just as detrimental, if not more harmful, to overall quality of life and health than medical diagnoses can be. There was no date restriction. p3 | mixed |
| Pilkington 2021 | self-report | Emotional neglect: A child's emotional needs are not met as 'his or her environment is missing something important, such as stability, understanding, or love' (Young et al., 2003, p. 10). Physical neglect Parent is physically absent or does not adequately meet child's physical or supervisory needs. This includes inadequate attention or monitoring, including leaving a child alone for more than a reasonable period or parenting a child while affected by alcohol or other drugs. Physical abuse: Physical violence including hitting, choking, threatening violence and physical punishment. Sexual abuse: Sexual experiences with an adult before age 16, sexual experiences with a parent or other traumatic sexual experiences p573 | analysed one or more of the 18 early maladaptive schemas(as defined by Young et al.) p571 | mixed |
| Pinquart 2017a | self-report | The studies assessed parental warmth, behavioral control, harsh control, psychological control, autonomy granting (vs. overprotection), and/or parenting styles defined by Maccoby and Martin (1983). p620 | They assessed internalizing symptoms of the children (e.g., anxiety, depression, sum measures of internalizing symptoms). p620 | mixed |
| Pinquart 2017b | nr | The studies assessed parental warmth, behavioral control, harsh control, psychological control, autonomy granting (vs. overprotection), and/or parenting styles defined by Maccoby and Martin (1983). p876 | They assessed externalizing problems of the children (e.g., aggressive behavior, delinquency, and sum measures of externalizing problems). p876 | mixed |
| Pinquart 2021 | nr | The studies assessed parenting styles defined by Baumrind (1966) and/or Maccoby and Martin (1983). p466 | They assessed the level of moral reasoning based on Kohlberg (1984), Gilligan (1977), or Eisenberg (Eisenberg-Berg, 1979) in pre-schoolers, pupils, or college students. p466 | mixed |
| Pinquart 2019 | self-report | Parenting styles were most often assessed with the Parental Authority Questionnaire (Buri 1991; 56 studies), the Parenting Styles and Dimensions Questionnaire (PSDQ; Robinson et al. 2001; 10 studies), and related instruments (50 studies). p2020 | Self-esteem was assessed with the Rosenberg Self-Esteem Scale (Rosenberg 1965; 68 studies), the Coopersmith Self-Esteem Inventory (Coopersmith 1967; 10 studies), versions of Harter’s Self-Perception Profile (Harter 1985; Harter 1988; 8 studies), and related instruments (30 studies). p2020 | mixed |
| Porter 2020 | nr | All studies were required to have a systematic quantitative measure of childhood adversity defined as reporting neglect, abuse, bullying or the loss of parents before the age of 19. p8 | diagnosis BPD according to the Diagnostic and Statistical Manual of Mental Disorders (DSM-III, DSM-IIIR, DSM-IV, DSM-IV-TR & DSM-5) or International Classification of Diseases (ICD-9 or ICD-10) of Emotionally Unstable Personality Disorder according to the International Classification of Diseases system p8 | mixed |
| Porter 2018 | self-report | Childhood adversity was defined as experiencing neglect, abuse, bullying or the loss of parents before the age of 19 p16 | BPD according to the Diagnostic and Statistical Manual (DSM-III, DSM-IIIR, DSMIV, DSM-IV-TR & DSM-5) or ICD diagnosis of Emotionally Unstable Personality Disorder according to the International Classification of Diseases (ICD-9 or ICD-10). p16 | mixed |
| Racine 2021 | nr | Adverse Childhood Experiences (ACEs) included cumulative retrospective self-reports of child adversity prior to the age of 18 years, including maltreatment and household dysfunction p2 | Prenatal depressive symptoms included depressive symptoms either self-reported (e.g., Center for Epidemiologic Studies Depression Scale) or assessed (e.g., diagnostic codes or diagnosed depressive disorder) during pregnancy. Postpartum depressive symptoms included depressive symptoms either self-reported Racine et al. BMC Psychiatry (2021) 21:28 Page 2 of 10 (e.g., Edinburgh Postnatal Depression Scale) or assessed (e.g., diagnosis of depression) after the birth of a child and prior to 12-months postpartum. Prenatal anxiety symptoms included anxiety symptoms either self-reported (e.g., State Trait Anxiety Scale) or assessed (e.g., diagnosed anxiety disorder) during pregnancy. Postpartum anxiety symptoms included those either self-reported (e.g., Generalized Anxiety Disorder, 7-item) or assessed (e.g., diagnosed disorder) after the birth of a child and prior to 12months postpartum. Perinatal depression and perinatal anxiety referred to measurements of anxiety and depression that occurred in both pregnancy and the postpartum period. p2 | mixed |
| Rafiq 2018 | self-report | childhood adversities considered in the current evidence synthesis (i.e. physical abuse, sexual abuse, emotional abuse, physical neglect, emotional neglect, natural disasters, mass violence and bullying), p510 | (i) schizophrenia and related psychotic disorders; (ii) personality disorders; or (iii) bipolar disorder based on ICD-9,ICD-10, DSM-III, DSM-III-R, DSM-IV, DSM-IV-TR or DSM-5 diagnostic criteria. p511 | mixed |
| Ran 2022 | mixed | described as any form of physical and emotional ill treatment, sexual abuse, neglect, and exploitation, resulting in actual or potential harm to children’s dignity, health, or development p3462 | Aggression can be regarded as a forceful behavior, attitude, or action which is conveyed physically, verbally, or symbolically p3462 | mixed |
| Ranu 2022 | mixed | described as any form of physical and emotional ill treatment, sexual abuse, neglect, and exploitation, resulting in actual or potential harm to children’s dignity, health, or development p3462 | Aggression can be regarded as a forceful behavior, attitude, or action which is conveyed physically, verbally, or symbolically p3462 | mixed |
| Shamblaw 2018 | self-report | all participants with POE (or polydrug exposure including opioids) p106 | hyperactive/impulsive and/or inattentive behaviors reported via caregiver or teacher ratings p106 | mixed |
| Silva 2017 | mixed | exposure: clear definition (given by the authors) of physical, sexual, or psychological violence perpetrated by the intimate partner during pregnancy p473 | outcome: behavioral, externalizing, and/or internalizing problems in children and adolescents, evaluated with validated tools p473 | mixed |
| Smith-Marek 2015 | nr | This meta-analysis concentrates on the first two phenomena: (a) children witnessing IPV and (b) children experiencing physical abuse and later involvement in violent intimate relationships. | nr | retrospective |
| Smolak 2002 | Mixed | Almost all of the studies defined the abused and non-abused groups based on whether people had experienced CSA as determined by a scale like FinkelhorÕs (Finkelhor & Browne, 1986). P139 | Eating Disorders Inventory (EDI) and the Eating Attitudes Test (EAT), bulimia measures, and clinical diagnoses P 138 | mixed |
| Souama 2023 | self-report | The main exposure was the presence of childhood maltreatment in any of the following categories: physical, emotional, and/or sexual abuse, before the age of 18. Physical and emotional maltreatment were defined by the following: (1) self-reported history of regular or more frequent abuse (“often true”, “very often true”, “regularly”, “often”, or “very often” frequency ratings depending on the instrument) when a frequency assessment was available or (2) self-reported history of abuse in case of a dichotomous assessment. Sexual abuse was defined by the report of at least one occurrence of sexual abuse in childhood. Cases of childhood maltreatment were identified when criteria were met for either maltreatment type. Neglect was not included in the definition of childhood maltreatment because participating studies either did not assess physical and emotional neglect (n=6) or assessed them in discrepant manners. p3 | The presence of depression was defined by the following: (1) the presence of a lifetime (eight cohorts) or current (one cohort) diagnosis of major depressive disorder assessed with (semi-)structured clinical interviews or (2) current depressive symptomatology (four cohorts) assessed with self-report scales using validated clinical cut-offs. Cohort-specific measures and criteria used to identify depression cases p3 | mixed |
| Tan 2023 | self-report | ACEs were measured by ACE-IQ; p2 | outcome variable defined as the incidence of depression; p2 | observational studies |
| Tang 2020 | nr | reporting at least one psychosocial risk factor; p156 | using measures of qualified psychometrics to assess risk factors and depressive symptoms p156 | mixed |
| Tetik 2021 | mixed | Form, definition, and age of abuse can differ according to the assessment method used, such as self-rated scales or clinical interviews. p1557 | diagnosis of vaginismus or/and dyspareunia and the presence of a control group; iv) diagnosis of vaginismus and dyspareunia assessed by clinical or structured interviews; p1557 | mixed |
| Todorov 2023 | mixed | specified at least one of the maltreatment criteria (i.e., physical abuse, sexual abuse, emotional abuse, physical neglect, emotional neglect). p3 | included a measure of CU traits and a measure of maltreatment, p3 | mixed |
| Toutountzidis 2022 | self-report | include a measure of childhood trauma for events before the age of 18 years (excluding peer victimisation; i.e., bullying; to assess for trauma where the perpetrator is an adult), | test for associations with schizotypy using any standardised measure of either single symptom/trait or multidimensional schizotypal personality traits in adults ( | mixed |
| Trentacosti 2021 | nr | Childhood maltreatment: Any act or series of acts of commission or omission by a parent or other caregiver (e.g., clergy, coach, teacher) that results in harm, potential for harm, or threat of harm to a child (Wissink, Van Vugt, Moonen, Stams, & Hendriks, 2015). Child sexual abuse: A form of child abuse that includes sexual activity with a minor, including but not limited to rape, fondling, sexual assault, exposure, voyeurism, and the commercial sexual exploitation of children (Norman et al., 2012). Child physical abuse: Purposefully hurting a child causing injuries such as bruises, broken bones, burns, and cuts (Norman et al., 2012). Child emotional abuse: This is described as emotional maltreatment or emotional neglect of a child and can involve intentionally trying to scare, humiliate, isolate or ignore a child (Slep, Heyman, & Snarr, 2011). Child neglect: The lack of meeting a child's basic needs, including the failure to provide adequate health care, supervision, clothing, nutrition, housing as well as their physical, emotional, social, educational and safety needs (U.S. Department of Health and Human Services, 2007). | Borderline Personality Disorder (BPD): A severe form of psychopathology depicted by instability of affect, self-harm, impulsivity, identity disturbance and chaotic interpersonal relationships (Tomko, Trull, Wood, & Sher, 2014). | mixed |
| Trotta 2015 | Mixed | The term ‘childhood adversity’ is a broad concept which includes child maltreatment (all forms of physical and/or emotional ill-treatment, sexual abuse, neglect or negligent treatment or commercial or other exploitation), peer victimization (e.g. bullying), experiences of parental loss and separation, war-related trauma, natural disasters, and witnessing domestic or non-domestic violence (Butchart et al. 2006). P2481 | diagnosis and symptoms | Mixed |
| Varese 2012 | Mixed | Measures of childhood adversity and trauma were considered eligible if: (1) the adverse events were assessed at the individual level and (2) exposure was specifically measured prior to the age of 18 (including measures assessing trauma in childhood and adolescence without additional timing details). Types of trauma included in the current meta-analysis were defined as: childhood sexual abuse (sexual acts toward a child, including intercourse, touching, etc.), childhood physical abuse (violent acts leading to physical injury or harm, such as harsh physical punishment), childhood emotional abuse (exposure to behaviour that might result in trauma, such as harshness, name calling by parents during childhood), childhood physical neglect (failure of those who are responsible for physical care to provide this care during childhood, e.g., by failing to provide food or clothes), childhood emotional neglect (failure of those who are responsible to provide emotional care to provide this care during childhood, e.g., by being unresponsive to a child’s emotional needs), and bullying (an act of repetitively aggressive behavior by a peer with the intention to hurt the child, such as physical assault or intimidation or repeated name-calling). Parental death was defined as death of one of the parents before the age of 18. Parental loss or separation was deemed only eligible if this was equal to parental death due to the high heterogeneity in the definition of separation (varying between being separated from one of the parents for a period of 2 weeks to parental death). P 662 | diagnosis and symptoms | mixed |
| Vibhakar 2019 | mixed | All study participants were exposed to trauma as defined by the A1 Criteria for PTSD in either DSM-IV or DSM-5, or separate data for the trauma-exposed group was available; (exposure to trauma was determined by study authors typically by a demographic survey assessing exposure, or living in an area affected by a disaster) | We included both self-report and diagnostic (i.e. interview) measures. We recorded the informant, the type of measure, cut-off score used, the period for which depression was assessed (1–6 months, <1 year, >1 year, unknown) and how long after the trauma the depression measurement was administered. | mixed |
| Vonderlin 2018 | self-report | Population with any form of childhood abuse/neglect (age <18); ( | use of the DES (Dissociative Experience Scale) | mixed |
| Vu 2016 | nr | ) The study included a measurement of parental physical IPV. Thus, our definition of children's exposure to IPV was children living in families in which occurrences of physical IPV were reported. This definition is consistent with the definitions used in other reviews | The study examined child adjustment problems within the domains of externalizing and/or internalizing problems. Studies that only included other child adjustment variables (e.g., those related to academic functioning and physical health outcomes) were excluded | mixed |
| Wang 2022a | self-report | As shown in Table 1, five assessment tools were used to evaluate CSA, and six assessment tools were used to evaluate sexual dysfunction in 15 studies. Most of the studies were conducted by using participants’ self-reports, four studies (Kinzl et al., 1995; Sarwer & Durlak, 1996; Sarwer et al., 1997; Steel & Herlitz, 2007) used the Diagnostic and Statistical Manual of Mental Disorders (DSM) to assess sexual function, and two studies (Lalchandani et al., 2020; Luo et al., 2008) used assessment instruments were from the National Health and Social Life Survey Of American (Carpenter et al., 2009). Definitions of CSA and sexual functioning also varied among the included studies. This variation in definitions poses a challenge for the consistent analysis and clarification of CSA and sexual dysfunction. Table 2 shows the definitions of CSA used in the studies, as well as the relevant indicators used to assess sexual function. | Definitions of CSA and sexual functioning also varied among the included studies. This variation in definitions poses a challenge for the consistent analysis and clarification of CSA and sexual dysfunction. no further definition | mixed |
| Wang 2022b | self-report | As shown in Table 1, five assessment tools were used to evaluate CSA, and six assessment tools were used to evaluate sexual dysfunction in 15 studies. Most of the studies were conducted by using participants’ self-reports, four studies (Kinzl et al., 1995; Sarwer & Durlak, 1996; Sarwer et al., 1997; Steel & Herlitz, 2007) used the Diagnostic and Statistical Manual of Mental Disorders (DSM) to assess sexual function, and two studies (Lalchandani et al., 2020; Luo et al., 2008) used assessment instruments were from the National Health and Social Life Survey Of American (Carpenter et al., 2009). Definitions of CSA and sexual functioning also varied among the included studies. This variation in definitions poses a challenge for the consistent analysis and clarification of CSA and sexual dysfunction. Table 2 shows the definitions of CSA used in the studies, as well as the relevant indicators used to assess sexual function. | Definitions of CSA and sexual functioning also varied among the included studies. This variation in definitions poses a challenge for the consistent analysis and clarification of CSA and sexual dysfunction. no further definition | mixed |
| Watters 2023 | mixed | sample participants had a history of childhood trauma, abuse, or maltreatment; | studies included one of the four instruments of trait resilience; (c) studies included measures of individual outcomes of depression; | mixed |
| Willems 2018 | self-report | the study included a correlation between family violence (on any relational level) and self-control | no further definition | mixed |
| Wilson 2010 | mixed | Child physical abuse typically is defined as any nonaccidental physical injury to a child caused by a parent or caretaker, whereas child neglect is a failure to meet parental obligations with regard to food, clothing, shelter, supervision, education, or medical care that endangers the child’s physical or psychological health (Barnett, Miller-Perrin, & Perrin, 2005). P 541 | The second category, aversive behavior, involves verbal or nonverbal actions by a child that display negative affect and hence are likely experienced as unpleasant by the parent. Aversive behaviors thus communicate anger, annoyance, disapproval, and/or resistance. Examples of specific behavioural indicators include vocal negative, physical aggression, verbal aggression, and noncompliance (Bousha & Twentyman, 1984) as well as hostility, oppositionality, and demandingness (Lau, Valerie, McCarty, & Weitz, 2006). Although aversiveness could be seen simply as the flip side of positivity (i.e., negative rather than positive valence), children frequently engage in aversive behaviors during "power struggles" in which they resist parental attempts to regulate their actions; thus aversive behaviors also may be interpreted in terms of dominance or control (Bugental & Happaney, 2000; Reid, 1986). P 542 | Mixed |
| Winsper 2016 | mixed | Participants were 19 years of age or under at index assessment, as consistent with the World Health Organisation (WHO) definition of adolescence (Sacks, 2003); | The main outcome parameter was BPD diagnosis, which was defined according to definitions used in the individual studies. Studies were included if they reported a dichotomous BPD outcome, which was assessed with an established interview or scale (e.g., the Child Diagnostic Interview for Borderlines [C-DIB]) and defined by the study as “clinically relevant” according to predetermined thresholds. Our secondary outcome parameter was BPD symptoms measured continuously with any established BPD scale (e.g., McLean Screening Instrument for BPD). | mixed |
| Witt 2019 | nr | no further definition | all participants had presented either to the accident and emergency department, general hospital departments, dedicated suicide prevention centres, and/or mental health services (including both in and out-patient services) following an episode of non-fatal self-harm; | mixed |
| Wolfe 2003 | mixed | nr | Note. CBCL = Child Behavior Checklist; (R)BPC = (Revised) Behavior Problem Checklist; YSR = Youth Self-Report; ECBI = Eyberg Child Behavior Inventory. | Mixed |
| Xiao 2023 | mixed | The term “emotional abuse” refers to the commission of hostile acts by the caregivers toward the child (McGee & Wolfe, 1991) P3050 | suicidal ideation (i.e., Positive and Negative Suicide Ideation). P3054 | mixed |
| Yang 2024 | self-report | Childhood maltreatment (CM) encompasses the abuse and neglect of individuals under 16 years old, involving actual or potential harm by a parent or caregiver, and is recognized as a global public health issue. It typically encompasses physical, emotional, and sexual abuse, further categorized into physical abuse and neglect, emotional abuse and neglect, and sexual abuse (Bernstein et al., 2003). P | Mentalizing serves as a comprehensive term encompassing essential mental processes that revolve around various facets of social cognition, including empathy, mindfulness, theory of mind (ToM), alexithymia, and insight (Choi-Kain & Gunderson, 2008). P 2 | cross-sectional |
| Yeo 2024 | self-report | Adverse childhood experiences (ACEs) refer to traumatic experiences in the first 18 years of life, such as abuse, neglect, and living in a stressful household or community environment (Boullier and Blair, 2018). P 387 | Following National Institutes of Health Roundtable (2018), positive well-being is conceptualized as emotional well-being (EWB) with three components: (a) eudaimonia—a sense of meaning and purpose in life; (2) evaluative well-being—reflective, general judgments (or perceptions) of life satisfaction; and (3) hedonic (or experiential) wellbeing—momentary emotional states. P388 | mixed |
| Yu 2022 | self-report | Five main ACE subtypes, including sexual abuse, physical abuse, emotional abuse, neglect, and family dysfunction, were extracted. | no further definition | mixed |
| Yu 2017 | nr | no further definition | no further definition | mixed |
| Zatti 2017 | informant | no further definition | We excluded all studies that covered only the risk of suicide without SA as well as those who actually completed suicide. Studies with subjects with active suicide ideation, but no SA, were also excluded. | mixed |
| Zhang 2023a | self-report | some of or all the samples had experienced one or more of the five types of child maltreatment, | empathy were assessed with reliable and valid measurement tools, | mixed |
| Zhang 2023b | self-report | some or all samples experienced child maltreatment, such as physical abuse, emotional abuse, sexual abuse, emotional neglect, or physical neglect; p2 | self-compassion assessed using reliable and valid measurement tools; p2 | mixed |
| Zhang 2022a | self-report | child maltreatment such as physical abuse, emotional abuse, sexual abuse, emotional neglect, or physical neglect; | Regarding the measurement of self-esteem, most studies used Rosenberg’s Self-Esteem Scale (RSE) (about 80.79%). The remaining studies used other tools, such as the Coopersmith Self-Esteem Inventory or self-developed scales. | mixed |
| Zhang 2021 | self-report | As there is no conclusive list of ACEs, we felt it was preferable to include as many types of ACEs as possible in the meta-analysis and thereby produce a richer understanding. In addition to the 10 items in the early ACE studies (Felitti et al., 1998), we applied the extended list (physical abuse, sexual abuse, emotional abuse, emotional neglect, physical neglect, exposure to domestic violence, household substance abuse, household mental health problems, parental separation or divorce, parental problems with police, spanking, peer victimization, household gambling problems, foster care placement or child protection agency contact, poverty, and neighborhood safety) suggested by a recent factor analysis study based on data from 1000 children p2586 | adults with diagnosis of PD (panic group) p2586 | mixed |
| Zhang 2022b | self-report | Dichotomous or continuous measurement of any form of ACEs. p3 | Diagnosis of subsequent ADHD or ADD according toanystandardizedcriteria,suchasDSM-III,DSM-III-R,DSM-IV,DSM-IV-TR,DSM-5,orICD-10;ordiagnosticinformationforADHDcouldbeobtainedfromreliablerecords,likevaccine-relatedrecords,clinicregisters, hospital records, and the like; or self-report and informed guardian-reported diagnosis for ADHD made by a professional p3 | mixed |
| Zhang 2023c | self-report | Child abuse, an important issue in the worldwide, refers to any form of psychological/or physical abuse, sexual abuse, neglect or negligent handling, or commercial or other exploitation, leading to actual or potential harm to the health survival, development or dignity of the child in the situation of a relationship of responsibility, trust, or power (Alazri & Hanna, 2020 Gubbels et al., 2021; Zhang, Ma et al. 2022). P 2 | Internet addiction is generally defined as a kind of problematic Internet use behavior, referring to individual’s excessive use of the Internet, resulting in neurological damage, psychological distress, and reducing socialization in daily life (Servidio et al., 2021). P2 | cross-sectional |
| Zhu 2023a | mixed | Adverse Childhood Experiences (ACEs) were assessed using cumulative retrospective self-reports or file review from child abuse P 4 records. Exposure to child adversity included maltreatment and household dysfunction experienced prior to age 18. Child adversity was measured using either the original 8-item ACEs (Felitti et al., 1998), which included physical abuse, sexual abuse, emotional abuse, parent substance use, parent mental health issues, parent divorce or separation, parent incarceration, and exposure to domestic violence; the 10-item measure, which included additional items assessing physical and emotional neglect; or an alternative composite measure of ACEs. | (1) drug use, (2) illicit drug use, (3) street drug use (ST1) | retrospective |
| Zhu 2023b | self-report | nr | Gratitude is a moral emotion that arises from recognizing the benevolent intentions of others (McCullough et al., 2001; Tsang et al., 2006). P 2 | cross-sectional |

*Contains direct quotations from papers; nr= not reported; mixed assessment = combination of self-report and informant report’ mixed study design = mix of study designs in meta-analysis (e.g., cross-sectional and longitudinal)

# Appendix S7: Moderator and outlier analysis

Table 1: Moderator analysis: Quality

| Mental health outcome | Quality rating | k | r | 95% CI (L) | 95% CI (U) | Tau^2^ | tau | I^2^ | p value |
| --- | --- | --- | --- | --- | --- | --- | --- | --- | --- |
| Externalising | High | 16 | 0.22 | 0.17 | 0.28 | 0.01 | 0.11 | 99.70% | 0.33 |
|  | L/M | 16 | 0.19 | 0.16 | 0.23 | 0.00 | 0.07 | 98.80% |  |
| Internalising | High | 24 | 0.22 | 0.19 | 0.25 | 0.01 | 0.08 | 99.80% | 0.83 |
|  | L/M | 22 | 0.22 | 0.20 | 0.25 | 0.00 | 0.06 | 99.70% |  |
| Thought Problems | High | 23 | 0.25 | 0.20 | 0.29 | 0.01 | 0.11 | 97.70% | 0.40 |
|  | L/M | 15 | 0.22 | 0.18 | 0.27 | 0.01 | 0.08 | 99.10% |  |
| Suicidal distress | High | 12 | 0.24 | 0.17 | 0.31 | 0.01 | 0.12 | 99.90% | 0.47 |
|  | L/M | 7 | 0.21 | 0.14 | 0.28 | 0.01 | 0.08 | 99.10% |  |
| Substance misuse | High | 10 | nr | nr | nr | nr | nr | nr | nr |
|  | L/M | 3 | nr | nr | nr | nr | nr | nr |  |
| Other psychological | High | 28 | 0.21 | 0.17 | 0.24 | 0.01 | 0.09 | 99.60% | 0.07 |
|  | L/M | 22 | 0.28 | 0.20 | 0.35 | 0.03 | 0.18 | 99.20% |  |

High = high quality; L/M = low/moderate quality; nr = not reported

Table 2: Moderator Analysis: Transformed effect size

| Mental health outcome | Effect Size  converted to R? | k | r | 95% CI (L) | 95% CI (U) | Tau^2^ | tau | I^2^ | p value |
| --- | --- | --- | --- | --- | --- | --- | --- | --- | --- |
| Externalising | Yes | 19 | 0.22 | 0.17 | 0.26 | 0.01 | 0.10 | 99.60% | 0.4153 |
|  | No | 13 | 0.19 | 0.15 | 0.24 | 0.01 | 0.08 | 99.20% |  |
| Internalising | Yes | 30 | 0.23 | 0.20 | 0.26 | 0.01 | 0.07 | 99.90% | 0.1953 |
|  | No | 16 | 0.20 | 0.17 | 0.24 | 0.00 | 0.06 | 99.10% |  |
| Thought Problems | Yes | 23 | 0.27 | 0.22 | 0.31 | 0.01 | 0.11 | 98.50% | 0.01 |
|  | No | 15 | 0.19 | 0.16 | 0.22 | 0.00 | 0.05 | 93.60% |  |
| Suicidal distress | Yes | 17 | nr | nr | nr | nr | nr | nr | nr |
|  | No | 2 | nr | nr | nr | nr | nr | nr | nr |
| Substance misuse | Yes | 12 | nr | nr | nr | nr | nr | nr | nr |
|  | No | 1 | nr | nr | nr | nr | nr | nr | nr |
| Other psychological | Yes | 29 | 0.23 | 0.20 | 0.26 | 0.01 | 0.08 | 99.40% | 0.45 |
|  | No | 21 | 0.26 | 0.17 | 0.34 | 0.04 | 0.19 | 99.60% |  |

Table 3: Moderator Analysis Estimated N

| Mental health outcome | Estimated N | k | r | 95% CI (L) | 95% CI (U) | Tau^2^ | tau | I^2^ | p value |
| --- | --- | --- | --- | --- | --- | --- | --- | --- | --- |
| Externalising | No | 21 | 0.20 | 0.16 | 0.24 | 0.01 | 0.09 | 99.60% | 0.87 |
|  | Yes | 11 | 0.21 | 0.15 | 0.27 | 0.01 | 0.09 | 99.30% |  |
| Internalising | No | 37 | 0.22 | 0.20 | 0.24 | 0.00 | 0.06 | 99.70% | 0.71 |
|  | Yes | 9 | 0.21 | 0.14 | 0.28 | 0.01 | 0.10 | 99.90% |  |
| Thought Problems | No | 29 | 0.23 | 0.20 | 0.27 | 0.01 | 0.10 | 98.10% | 0.67 |
|  | Yes | 9 | 0.25 | 0.17 | 0.32 | 0.01 | 0.10 | 99.00% |  |
| Suicidal distress | Yes | 3 | nr | nr | nr | nr | nr | nr | nr |
|  | No | 16 | nr | nr | nr | nr | nr | nr |  |
| Substance misuse | No | 9 | 0.18 | 0.08 | 0.27 | 0.02 | 0.13 | 99.70% | 0.28 |
|  | Yes | 4 | 0.24 | 0.09 | 0.38 | 0.01 | 0.10 | 100.00% |  |
| Other psychological | No | 40 | 0.25 | 0.20 | 0.29 | 0.02 | 0.15 | 99.60% | 0.21 |
|  | Yes | 10 | 0.21 | 0.18 | 0.25 | 0.00 | 0.05 | 98.50% |  |

Table 4: Moderator Analysis: Homogeneity of variance

| Mental health outcome | Rating | k | r | 95% CI (L) | 95% CI (U) | Tau^2^ | tau | I^2^ | p value |
| --- | --- | --- | --- | --- | --- | --- | --- | --- | --- |
| Externalising | Low heterogeneity | 8 | 0.20 | 0.18 | 0.23 | 0.00 | 0.03 | 90.10% | 0.83 |
|  | High heterogeneity | 24 | 0.21 | 0.17 | 0.25 | 0.01 | 0.10 | 99.60% |  |
| Internalising | Low heterogeneity | 5 | 0.20 | 0.13 | 0.26 | 0.00 | 0.05 | 93.80% | 0.30 |
|  | High heterogeneity | 41 | 0.22 | 0.20 | 0.25 | 0.01 | 0.07 | 99.80% |  |
| Thought Problems | Low heterogeneity | 6 | 0.17 | 0.07 | 0.27 | 0.01 | 0.10 | 97.70% | 0.06 |
|  | High heterogeneity | 32 | 0.25 | 0.22 | 0.28 | 0.01 | 0.09 | 98.60% |  |
| Suicidal distress | Low heterogeneity | 5 | 0.15 | 0.06 | 0.24 | 0.01 | 0.07 | 96.70% | **0.01** |
|  | High heterogeneity | 14 | 0.26 | 0.20 | 0.31 | 0.01 | 0.10 | 99.90% |  |
| Substance misuse | Low heterogeneity | 2 | nr | nr | nr | nr | nr | nr |  |
|  | High heterogeneity | 11 | nr | nr | nr | nr | nr | nr |  |
| Other psychological | Low heterogeneity | 8 | 0.26 | 0.15 | 0.37 | 0.02 | 0.14 | 99.40% | 0.60 |
|  | High heterogeneity | 42 | 0.23 | 0.19 | 0.28 | 0.02 | 0.14 | 99.60% |  |

Table 5: Moderator Analysis: Publication bias

| Mental health outcome | Rating | k | r | 95% CI (L) | 95% CI (U) | Tau^2^ | tau | I^2^ | p value |
| --- | --- | --- | --- | --- | --- | --- | --- | --- | --- |
| Externalising | No publication bias | 17 | 0.19 | 0.16 | 0.22 | 0.00 | 0.07 | 99.00% | 0.28 |
|  | Publication bias indicated | 15 | 0.22 | 0.17 | 0.28 | 0.01 | 0.11 | 99.70% |  |
| Internalising | No publication bias | 21 | 0.23 | 0.20 | 0.26 | 0.01 | 0.07 | 99.50% | 0.46 |
|  | Publication bias indicated | 25 | 0.21 | 0.19 | 0.24 | 0.01 | 0.07 | 99.90% |  |
| Thought Problems | No publication bias | 18 | 0.24 | 0.20 | 0.27 | 0.00 | 0.07 | 97.20% | 0.87 |
|  | Publication bias indicated | 20 | 0.24 | 0.19 | 0.29 | 0.01 | 0.12 | 98.90% |  |
| Suicidal distress | No publication bias | 6 | 0.24 | 0.13 | 0.34 | 0.01 | 0.11 | 98.20% | 0.84 |
|  | Publication bias indicated | 13 | 0.23 | 0.16 | 0.29 | 0.01 | 0.11 | 99.90% |  |
| Substance misuse | No publication bias | 4 | 0.21 | -0.06 | 0.46 | 0.03 | 0.18 | 99.80% | 0.79 |
|  | Publication bias indicated | 9 | 0.19 | 0.12 | 0.26 | 0.01 | 0.10 | 99.90% |  |
| Other psychological | Publication bias indicated | 24 | 0.25 | 0.18 | 0.32 | 0.03 | 0.17 | 99.10% | 0.49 |
|  | No publication bias | 26 | 0.23 | 0.18 | 0.27 | 0.01 | 0.10 | 99.70% |  |

Table 6: Moderator Analysis: Outliers

| Mental health outcome | Outlier ratings | k | r | CI(L) | CI(U) | tau^2 | tau | i2 |
| --- | --- | --- | --- | --- | --- | --- | --- | --- |
| Externalising | Outliers removed  (k = 1; Hughes 2017) | 31 | 0.20 | 0.17 | 0.22 | 0.005 | .07 | 99.1% |
| Internalising | No outliers identified | nr | nr | nr | nr | nr | nr | nr |
| Thought Problems | Outliers removed  (k = 1; Porter 2018) | 37 | 0.23 | 0.20 | 0.26 | 0.01 | 0.08 | 98.50% |
| Suicidal distress | No outlier identified | nr | nr | nr | nr | nr | nr | nr |
| Substance misuse | No outlier identified | nr | nr | nr | nr | nr | nr | nr |
| Other psychological | Outliers removed  (k = 1; Cyr 2010 ) | 49 | 0.23 | 0.20 | 0.26 | 0.01 | 0.11 | 99.5% |

# Appendix S8: Quality Rating Scores

Table 1: Quality Rating Scores

| **Authors** | **Mental health outcome** | **Systematic Search** | **N above 1000?** | **Heterogeneity identified?** | **PB identified?** | **Quality**  **assessed?** | **T/A**  **double**  **coded?** | **FT**  **double coded?** | **Extraction double coded?** | **Qual**  **double**  **coded?** | **Qual score** | **Overall rating** |
| --- | --- | --- | --- | --- | --- | --- | --- | --- | --- | --- | --- | --- |
| Augsburger 2019 | Externalising | Yes | Yes | Yes/nr | No | Yes | Yes | Yes | Yes | Yes | 8 | high |
| Baldwin 2023 | Externalising | Yes | Yes | Yes/nr | Yes/nr | Yes | Yes | Yes | Yes | Yes | 7 | high |
| Braga 2017 | Externalising | Yes | Yes | Yes/nr | Yes/nr | Yes | No/nr | No/nr | Yes | Yes | 5 | L/M |
| Braga 2018 | Externalising | Yes | Yes | Yes/nr | No | Yes | No/nr | Yes | Yes | Yes | 7 | high |
| Cui 2020 | Externalising | No | Yes | Yes/nr | No | Yes | Yes | Yes | Yes | Yes | 7 | high |
| Evans 2008 | Externalising | No | Yes | Yes/nr | Yes/nr | No/nr | No/nr | Yes | Yes | No/nr | 3 | L/M |
| Fares-Otero 2023 | Externalising | Yes | No | No | No | Yes | Yes | Yes | Yes | Yes | 8 | high |
| Fitton 2017 | Externalising | Yes | Yes | Yes/nr | No | Yes | No/nr | No/nr | No/nr | No/nr | 4 | L/M |
| Gershoff 2016 | Externalising | Yes | Yes | Yes/nr | No | No/nr | Yes | Yes | Yes | No/nr | 6 | high |
| Godbout 2017 | Externalising | Yes | Yes | Yes/nr | No | No/nr | No/nr | No/nr | Yes | No/nr | 4 | L/M |
| Goncy 2021 | Externalising | Yes | Yes | Yes/nr | No | No/nr | No/nr | No/nr | Yes | No/nr | 4 | L/M |
| Green 2017 | Externalising | Yes | Yes | No | Yes/nr | Yes | No/nr | No/nr | Yes | Yes | 6 | high |
| Heerde 2019 | Externalising | Yes | Yes | Yes/nr | Yes/nr | Yes | Yes | Yes | Yes | Yes | 7 | high |
| Hughes 2017 | Externalising | Yes | Yes | Yes/nr | Yes/nr | Yes | Yes | Yes | Yes | Yes | 7 | high |
| Kitzmann 2003 | Externalising | Yes | Yes | No | Yes/nr | No/nr | No/nr | Yes | Yes | No/nr | 5 | L/M |
| Lavi 2019 | Externalising | Yes | Yes | Yes/nr | Yes/nr | No/nr | Yes | Yes | Yes | No/nr | 5 | L/M |
| Li 2020b | Externalising | Yes | Yes | Yes/nr | No | No/nr | No/nr | No/nr | Yes | No/nr | 4 | L/M |
| MacMillan 1999 | Externalising | Yes | Yes | Yes/nr | Yes/nr | No/nr | No/nr | No/nr | Yes | No/nr | 3 | L/M |
| Magalhaes 2023 | Externalising | Yes | Yes | Yes/nr | No | No/nr | Yes | Yes | No/nr | No/nr | 5 | L/M |
| Martijn 2020 | Externalising | Yes | Yes | No | No | No/nr | No/nr | No/nr | Yes | No/nr | 5 | L/M |
| Mootz 2022 | Externalising | Yes | Yes | Yes/nr | No | No/nr | No/nr | Yes | Yes | No/nr | 5 | L/M |
| Neumann 1996 | Externalising | Yes | Yes | Yes/nr | Yes/nr | No/nr | No/nr | No/nr | No/nr | No/nr | 2 | L/M |
| Nilsen 2020 | Externalising | Yes | Yes | Yes/nr | Yes/nr | Yes | Yes | Yes | Yes | Yes | 7 | high |
| Norman 2013 | Externalising | Yes | Yes | No | Yes/nr | Yes | No/nr | Yes | Yes | Yes | 7 | high |
| Petruccelli 2019 | Externalising | Yes | Yes | Yes/nr | Yes/nr | Yes | Yes | Yes | Yes | Yes | 7 | high |
| Pinquart 2017a | Externalising | Yes | Yes | No | No | No/nr | No/nr | No/nr | Yes | No/nr | 5 | L/M |
| Ran 2022 | Externalising | Yes | Yes | Yes/nr | No | No/nr | No/nr | No/nr | Yes | No/nr | 4 | L/M |
| Ranu 2022 | Externalising | Yes | Yes | No | Yes/nr | Yes | Yes | Yes | Yes | Yes | 8 | high |
| Silva 2017 | Externalising | Yes | Yes | No | Yes/nr | Yes | Yes | No/nr | Yes | Yes | 7 | high |
| Smith-Marek 2015 | Externalising | Yes | Yes | Yes/nr | No | Yes | No/nr | No/nr | Yes | Yes | 6 | high |
| Vu 2016 | Externalising | Yes | Yes | Yes/nr | No | No/nr | Yes | Yes | Yes | No/nr | 6 | high |
| Zhu 2023 | Externalising | Yes | Yes | Yes/nr | No | No/nr | No/nr | No/nr | Yes | No/nr | 4 | L/M |
| Agnew-Blais 2016 | Internalising | Yes | Yes | Yes/nr | No | Yes | No/nr | No/nr | Yes | Yes | 6 | high |
| Alameda 2021 | Internalising | Yes | Yes | Yes/nr | Yes/nr | Yes | Yes | Yes | Yes | Yes | 7 | high |
| Amado 2015 | Internalising | Yes | Yes | Yes/nr | Yes/nr | No/nr | No/nr | No/nr | Yes | No/nr | 3 | L/M |
| Baldwin 2023 | Internalising | Yes | Yes | Yes/nr | Yes/nr | Yes | Yes | Yes | Yes | Yes | 7 | high |
| Bellis 2019 | Internalising | Yes | Yes | Yes/nr | Yes/nr | Yes | Yes | Yes | Yes | Yes | 7 | high |
| Braithwaite 2017 | Internalising | Yes | Yes | Yes/nr | Yes/nr | Yes | No/nr | No/nr | Yes | Yes | 5 | L/M |
| Cui 2020 | Internalising | No | Yes | Yes/nr | No | Yes | Yes | Yes | Yes | Yes | 7 | high |
| Evans 2008 | Internalising | No | Yes | No | Yes/nr | No/nr | No/nr | Yes | Yes | No/nr | 4 | L/M |
| Gardner 2019 | Internalising | Yes | Yes | Yes/nr | Yes/nr | No/nr | No/nr | Yes | Yes | No/nr | 4 | L/M |
| Gershoff 2016 | Internalising | Yes | Yes | Yes/nr | No | No/nr | Yes | Yes | Yes | No/nr | 6 | high |
| Gibb 2002 | Internalising | Yes | Yes | Yes/nr | No | No/nr | No/nr | No/nr | No/nr | No/nr | 3 | L/M |
| Hughes 2017 | Internalising | Yes | Yes | Yes/nr | No | Yes | Yes | Yes | Yes | Yes | 8 | high |
| Humphreys 2020 | Internalising | Yes | Yes | Yes/nr | Yes/nr | No/nr | No/nr | No/nr | Yes | No/nr | 3 | L/M |
| Infurna 2016 | Internalising | Yes | Yes | Yes/nr | No | No/nr | No/nr | No/nr | Yes | No/nr | 4 | L/M |
| Ip 2015 | Internalising | Yes | Yes | Yes/nr | No | Yes | Yes | Yes | Yes | Yes | 8 | high |
| Islas-Preciado 2021 | Internalising | Yes | Yes | No | Yes/nr | Yes | Yes | Yes | Yes | Yes | 8 | high |
| Jumper 1995 | Internalising | Yes | Yes | Yes/nr | Yes/nr | No/nr | No/nr | No/nr | No/nr | No/nr | 2 | L/M |
| Kitzmann 2003 | Internalising | Yes | Yes | No | Yes/nr | No/nr | No/nr | Yes | Yes | No/nr | 5 | L/M |
| Lai 2023 | Internalising | Yes | Yes | Yes/nr | No | No/nr | Yes | Yes | Yes | No/nr | 6 | high |
| LeMoult 2020 | Internalising | Yes | Yes | Yes/nr | No | Yes | No/nr | No/nr | Yes | No/nr | 5 | L/M |
| Li 2016 | Internalising | Yes | Yes | Yes/nr | No | Yes | No/nr | Yes | Yes | Yes | 7 | high |
| Li 2020a | Internalising | Yes | Yes | Yes/nr | No | Yes | No/nr | No/nr | Yes | Yes | 6 | high |
| Li 2022 | Internalising | Yes | Yes | Yes/nr | Yes/nr | Yes | Yes | Yes | Yes | Yes | 7 | high |
| Lindert 2014 | Internalising | No | Yes | Yes/nr | No | Yes | Yes | No/nr | No/nr | No/nr | 4 | L/M |
| Liu 2023a | Internalising | Yes | Yes | Yes/nr | No | Yes | Yes | Yes | Yes | Yes | 8 | high |
| MacMillan 1999 | Internalising | Yes | Yes | No | Yes/nr | No/nr | No/nr | No/nr | Yes | No/nr | 4 | L/M |
| Magalhaes 2023 | Internalising | Yes | Yes | Yes/nr | Yes/nr | No/nr | Yes | Yes | No/nr | No/nr | 4 | L/M |
| Nelson 2017 | Internalising | Yes | Yes | Yes/nr | No | Yes | No/nr | No/nr | Yes | Yes | 6 | high |
| Neumann 1996 | Internalising | Yes | Yes | Yes/nr | Yes/nr | No/nr | No/nr | No/nr | No/nr | No/nr | 2 | L/M |
| Norman 2013 | Internalising | Yes | Yes | Yes/nr | Yes/nr | Yes | No/nr | Yes | Yes | Yes | 6 | high |
| Paolucci 2001 | Internalising | Yes | Yes | Yes/nr | Yes/nr | Yes | No/nr | No/nr | No/nr | No/nr | 3 | L/M |
| Petruccelli 2019 | Internalising | Yes | Yes | Yes/nr | Yes/nr | Yes | Yes | Yes | Yes | Yes | 7 | high |
| Pinquart 2017b | Internalising | Yes | Yes | Yes/nr | Yes/nr | Yes | No/nr | No/nr | Yes | Yes | 5 | L/M |
| Racine 2021 | Internalising | Yes | Yes | Yes/nr | Yes/nr | Yes | Yes | No/nr | Yes | Yes | 6 | high |
| Shamblaw 2018 | Internalising | Yes | Yes | Yes/nr | No | Yes | Yes | No/nr | Yes | Yes | 7 | high |
| Silva 2017 | Internalising | Yes | Yes | No | Yes/nr | Yes | Yes | No/nr | Yes | Yes | 7 | high |
| Souama 2023 | Internalising | No | Yes | Yes/nr | Yes/nr | No/nr | No/nr | No/nr | No/nr | No/nr | 1 | L/M |
| Tan 2023 | Internalising | Yes | Yes | Yes/nr | No | Yes | Yes | Yes | Yes | Yes | 8 | high |
| Tang 2020 | Internalising | Yes | Yes | Yes/nr | No | Yes | Yes | Yes | Yes | Yes | 8 | high |
| Vibhakar 2019 | Internalising | Yes | Yes | Yes/nr | Yes/nr | No/nr | No/nr | Yes | Yes | No/nr | 4 | L/M |
| Vu 2016 | Internalising | Yes | Yes | Yes/nr | No | No/nr | Yes | Yes | Yes | No/nr | 6 | high |
| Wang 2022b | Internalising | Yes | Yes | Yes/nr | No | Yes | No/nr | No/nr | Yes | Yes | 6 | high |
| Watters 2023 | Internalising | Yes | Yes | Yes/nr | No | No/nr | No/nr | No/nr | No/nr | No/nr | 3 | L/M |
| Yu 2017 | Internalising | Yes | Yes | Yes/nr | No | No/nr | No/nr | No/nr | No/nr | No/nr | 3 | L/M |
| Zhang 2021 | Internalising | Yes | Yes | Yes/nr | Yes/nr | Yes | No/nr | No/nr | Yes | Yes | 5 | L/M |
| Mandelli 2015 | Internalising | No | Yes | Yes/nr | Yes/nr | Yes | No/nr | No/nr | No/nr | No/nr | 2 | L/M |
| Agnew-Blais 2016 | Thought problems | Yes | Yes | Yes/nr | No | Yes | No/nr | No/nr | Yes | Yes | 6 | high |
| Alameda 2021 | Thought problems | Yes | Yes | Yes/nr | No | Yes | Yes | Yes | Yes | Yes | 8 | high |
| Bailey 2018 | Thought problems | Yes | Yes | Yes/nr | No | Yes | No/nr | No/nr | Yes | Yes | 6 | high |
| Baldwin 2023 | Thought problems | Yes | No | Yes/nr | Yes/nr | Yes | Yes | Yes | Yes | Yes | 6 | high |
| Bodicker 2022 | Thought problems | Yes | Yes | Yes/nr | No | Yes | Yes | Yes | Yes | Yes | 8 | high |
| Boumpa 2022 | Thought problems | Yes | Yes | Yes/nr | No | Yes | No/nr | No/nr | No/nr | No/nr | 4 | L/M |
| Brewin 2000 | Thought problems | Yes | Yes | No | Yes/nr | No/nr | Yes | Yes | Yes | No/nr | 6 | high |
| Caslini 2016 | Thought problems | Yes | Yes | Yes/nr | No | Yes | Yes | Yes | Yes | Yes | 8 | high |
| Cri»ôan 2023 | Thought problems | Yes | Yes | Yes/nr | Yes/nr | Yes | No/nr | No/nr | No/nr | No/nr | 3 | L/M |
| De 2022 | Thought problems | Yes | Yes | Yes/nr | No | No/nr | Yes | Yes | Yes | No/nr | 6 | high |
| Dolan 2018 | Thought problems | Yes | Yes | Yes/nr | Yes/nr | Yes | No/nr | No/nr | No/nr | No/nr | 3 | L/M |
| Fossati 1999 | Thought problems | Yes | Yes | Yes/nr | Yes/nr | No/nr | No/nr | No/nr | No/nr | No/nr | 2 | L/M |
| Gao 2023b | Thought problems | Yes | Yes | Yes/nr | Yes/nr | Yes | No/nr | No/nr | No/nr | No/nr | 3 | L/M |
| Gardner 2019 | Thought problems | Yes | Yes | Yes/nr | Yes/nr | No/nr | No/nr | Yes | Yes | No/nr | 4 | L/M |
| Ip 2015 | Thought problems | Yes | Yes | Yes/nr | No | Yes | Yes | Yes | Yes | Yes | 8 | high |
| Lee 2021 | Thought problems | Yes | Yes | No | No | No/nr | No/nr | No/nr | Yes | No/nr | 5 | L/M |
| Leiva-Bianchi 2023 | Thought problems | Yes | Yes | Yes/nr | No | Yes | Yes | Yes | No/nr | No/nr | 6 | high |
| Longobardi 2022 | Thought problems | Yes | Yes | Yes/nr | No | Yes | Yes | Yes | Yes | Yes | 8 | high |
| MacMillan 1999 | Thought problems | Yes | Yes | No | Yes/nr | No/nr | No/nr | No/nr | Yes | No/nr | 4 | L/M |
| Matheson 2013 | Thought problems | Yes | Yes | Yes/nr | No | Yes | Yes | Yes | Yes | Yes | 8 | high |
| Molendijk 2017 | Thought problems | Yes | Yes | Yes/nr | No | Yes | Yes | Yes | Yes | Yes | 8 | high |
| Neumann 1996 | Thought problems | Yes | Yes | Yes/nr | Yes/nr | No/nr | No/nr | No/nr | No/nr | No/nr | 2 | L/M |
| Norman 2013 | Thought problems | Yes | Yes | Yes/nr | Yes/nr | Yes | No/nr | Yes | Yes | Yes | 6 | high |
| Ou 2021 | Thought problems | Yes | Yes | No | No | Yes | No/nr | No/nr | Yes | Yes | 7 | high |
| Palmier-Claus 2016 | Thought problems | Yes | Yes | Yes/nr | No | Yes | Yes | Yes | Yes | Yes | 8 | high |
| Paolucci 2001 | Thought problems | Yes | Yes | Yes/nr | Yes/nr | Yes | No/nr | No/nr | No/nr | No/nr | 3 | L/M |
| Pastore 2020 | Thought problems | Yes | Yes | Yes/nr | Yes/nr | Yes | Yes | Yes | Yes | Yes | 7 | high |
| Peh 2018 | Thought problems | Yes | Yes | Yes/nr | Yes/nr | Yes | No/nr | Yes | Yes | Yes | 6 | high |
| Porter 2018 | Thought problems | Yes | No | Yes/nr | Yes/nr | Yes | Yes | Yes | Yes | Yes | 6 | high |
| Porter 2020 | Thought problems | Yes | Yes | Yes/nr | Yes/nr | Yes | Yes | Yes | Yes | Yes | 7 | high |
| Rafiq 2018 | Thought problems | Yes | Yes | Yes/nr | Yes/nr | Yes | Yes | Yes | No/nr | No/nr | 5 | L/M |
| Smolak 2002 | Thought problems | Yes | Yes | Yes/nr | Yes/nr | No/nr | No/nr | No/nr | No/nr | No/nr | 2 | L/M |
| Toutountzidis 2022 | Thought problems | Yes | Yes | Yes/nr | Yes/nr | Yes | Yes | Yes | Yes | Yes | 7 | high |
| Trentacosti 2021 | Thought problems | Yes | Yes | Yes/nr | Yes/nr | No/nr | No/nr | No/nr | No/nr | No/nr | 2 | L/M |
| Trotta 2015 | Thought problems | Yes | Yes | No | No | Yes | No/nr | No/nr | No/nr | No/nr | 5 | L/M |
| Varese 2012 | Thought problems | Yes | Yes | Yes/nr | No | No/nr | Yes | Yes | Yes | No/nr | 6 | high |
| Vonderlin 2018 | Thought problems | Yes | Yes | Yes/nr | Yes/nr | No/nr | No/nr | No/nr | Yes | No/nr | 3 | L/M |
| Winsper 2016 | Thought problems | Yes | Yes | No | No | Yes | Yes | Yes | No/nr | No/nr | 7 | high |
| Agnew-Blais 2016 | Suicidal distress | Yes | Yes | No | No | Yes | No/nr | No/nr | Yes | Yes | 7 | high |
| Angelakis 2018 | Suicidal distress | Yes | Yes | Yes/nr | No | Yes | Yes | Yes | Yes | Yes | 8 | high |
| Angelakis 2020a | Suicidal distress | Yes | Yes | Yes/nr | Yes/nr | Yes | Yes | Yes | Yes | Yes | 7 | high |
| Angelakis 2020b | Suicidal distress | Yes | Yes | Yes/nr | No | Yes | Yes | Yes | Yes | Yes | 8 | high |
| Baldini 2023 | Suicidal distress | Yes | Yes | Yes/nr | Yes/nr | Yes | Yes | Yes | Yes | Yes | 7 | high |
| Baldwin 2023 | Suicidal distress | Yes | Yes | Yes/nr | Yes/nr | Yes | Yes | Yes | Yes | Yes | 7 | high |
| Castellvi 2017 | Suicidal distress | Yes | Yes | Yes/nr | Yes/nr | Yes | No/nr | No/nr | No/nr | No/nr | 3 | L/M |
| Duarte 2020 | Suicidal distress | Yes | Yes | No | No | Yes | Yes | Yes | Yes | Yes | 9 | high |
| Liu 2017 | Suicidal distress | Yes | Yes | Yes/nr | No | Yes | No/nr | No/nr | Yes | Yes | 6 | high |
| Liu 2018 | Suicidal distress | Yes | Yes | Yes/nr | Yes/nr | No/nr | Yes | No/nr | No/nr | No/nr | 3 | L/M |
| MacMillan 1999 | Suicidal distress | Yes | Yes | No | Yes/nr | No/nr | No/nr | No/nr | Yes | No/nr | 4 | L/M |
| Neumann 1996 | Suicidal distress | Yes | Yes | Yes/nr | Yes/nr | No/nr | No/nr | No/nr | No/nr | No/nr | 2 | L/M |
| Ng 2019 | Suicidal distress | Yes | Yes | Yes/nr | Yes/nr | No/nr | Yes | Yes | No/nr | No/nr | 4 | L/M |
| Norman 2013 | Suicidal distress | Yes | Yes | Yes/nr | Yes/nr | Yes | No/nr | Yes | Yes | Yes | 6 | high |
| Paolucci 2001 | Suicidal distress | Yes | Yes | Yes/nr | Yes/nr | Yes | No/nr | No/nr | No/nr | No/nr | 3 | L/M |
| Petruccelli 2019 | Suicidal distress | Yes | Yes | Yes/nr | Yes/nr | Yes | Yes | Yes | Yes | Yes | 7 | high |
| Witt 2019 | Suicidal distress | Yes | No | No | Yes/nr | Yes | Yes | Yes | Yes | Yes | 7 | high |
| Xiao 2023 | Suicidal distress | Yes | Yes | No | Yes/nr | Yes | Yes | Yes | Yes | Yes | 8 | high |
| Zatti 2017 | Suicidal distress | Yes | Yes | Yes/nr | No | Yes | No/nr | No/nr | No/nr | No/nr | 4 | L/M |
| Agnew-Blais 2016 | Substance misuse | Yes | Yes | Yes/nr | No | Yes | No/nr | No/nr | Yes | Yes | 6 | high |
| Baldwin 2023 | Substance misuse | Yes | Yes | Yes/nr | Yes/nr | Yes | Yes | Yes | Yes | Yes | 7 | high |
| Bellis 2019 | Substance misuse | Yes | Yes | Yes/nr | Yes/nr | Yes | Yes | Yes | Yes | Yes | 7 | high |
| De 2021 | Substance misuse | Yes | Yes | Yes/nr | Yes/nr | Yes | Yes | Yes | Yes | Yes | 7 | high |
| Gershoff 2016 | Substance misuse | Yes | Yes | Yes/nr | No | No/nr | Yes | Yes | Yes | No/nr | 6 | high |
| Halpern 2018 | Substance misuse | Yes | Yes | No | No | Yes | Yes | Yes | Yes | Yes | 9 | high |
| Hughes 2017 | Substance misuse | Yes | Yes | Yes/nr | No | Yes | Yes | Yes | Yes | Yes | 8 | high |
| Lucia 2020 | Substance misuse | No | Yes | Yes/nr | Yes/nr | Yes | Yes | Yes | No/nr | No/nr | 4 | L/M |
| MacMillan 1999 | Substance misuse | Yes | Yes | No | Yes/nr | No/nr | No/nr | No/nr | Yes | No/nr | 4 | L/M |
| Neumann 1996 | Substance misuse | Yes | Yes | Yes/nr | Yes/nr | No/nr | No/nr | No/nr | No/nr | No/nr | 2 | L/M |
| Norman 2013 | Substance misuse | Yes | Yes | Yes/nr | Yes/nr | Yes | No/nr | Yes | Yes | Yes | 6 | high |
| Petruccelli 2019 | Substance misuse | Yes | Yes | Yes/nr | Yes/nr | Yes | Yes | Yes | Yes | Yes | 7 | high |
| Zhu 2023a | Substance misuse | Yes | Yes | Yes/nr | Yes/nr | Yes | Yes | No/nr | Yes | Yes | 6 | high |
| Amado 2015 | other psychological difficulties | Yes | Yes | Yes/nr | Yes/nr | No/nr | No/nr | No/nr | Yes | No/nr | 3 | L/M |
| Baer 2006 | other psychological difficulties | No | No | Yes/nr | Yes/nr | No/nr | No/nr | No/nr | No/nr | No/nr | 0 | L/M |
| Baldwin 2023 | other psychological difficulties | Yes | Yes | Yes/nr | Yes/nr | Yes | Yes | Yes | Yes | Yes | 7 | high |
| Beaumont 2018 | other psychological difficulties | Yes | Yes | Yes/nr | No | No/nr | No/nr | No/nr | No/nr | No/nr | 3 | L/M |
| Carmichael 2019 | other psychological difficulties | Yes | Yes | Yes/nr | Yes/nr | Yes | No/nr | No/nr | No/nr | No/nr | 3 | L/M |
| Christy 2023 | other psychological difficulties | Yes | Yes | Yes/nr | No | Yes | Yes | Yes | Yes | Yes | 8 | high |
| Croft 2021 | other psychological difficulties | Yes | Yes | Yes/nr | Yes/nr | Yes | No/nr | Yes | Yes | Yes | 6 | high |
| Cyr 2010 | other psychological difficulties | No | No | Yes/nr | No | No/nr | No/nr | No/nr | Yes | No/nr | 2 | L/M |
| Deneault 2023 | other psychological difficulties | Yes | Yes | Yes/nr | Yes/nr | No/nr | No/nr | No/nr | No/nr | No/nr | 2 | L/M |
| Ditzer 2023 | other psychological difficulties | Yes | Yes | No | No | No/nr | Yes | Yes | Yes | No/nr | 7 | high |
| Downing 2022 | other psychological difficulties | Yes | Yes | Yes/nr | Yes/nr | Yes | No/nr | Yes | Yes | Yes | 6 | high |
| Fares-Otero 2023 | other psychological difficulties | Yes | No | Yes/nr | No | Yes | Yes | Yes | Yes | Yes | 7 | high |
| Francis 2023 | other psychological difficulties | Yes | Yes | Yes/nr | No | Yes | Yes | Yes | Yes | Yes | 8 | high |
| Gao 2023a | other psychological difficulties | Yes | Yes | Yes/nr | Yes/nr | Yes | No/nr | Yes | Yes | Yes | 6 | high |
| Gershoff 2016 | other psychological difficulties | Yes | Yes | Yes/nr | No | No/nr | Yes | Yes | Yes | No/nr | 6 | high |
| Gruhn 2020 | other psychological difficulties | Yes | Yes | Yes/nr | No | No/nr | No/nr | No/nr | No/nr | No/nr | 3 | L/M |
| Jumper 1995 | other psychological difficulties | Yes | Yes | Yes/nr | Yes/nr | No/nr | No/nr | No/nr | No/nr | No/nr | 2 | L/M |
| Kane 2017 | other psychological difficulties | Yes | Yes | Yes/nr | Yes/nr | No/nr | No/nr | No/nr | No/nr | No/nr | 2 | L/M |
| Kautz-Turnbull 2021 | other psychological difficulties | Yes | Yes | Yes/nr | No | No/nr | No/nr | No/nr | No/nr | No/nr | 3 | L/M |
| Khaleque 2015 | other psychological difficulties | Yes | Yes | No | No | No/nr | No/nr | No/nr | No/nr | No/nr | 4 | L/M |
| Khan 2022 | other psychological difficulties | Yes | Yes | Yes/nr | No | Yes | Yes | Yes | Yes | Yes | 8 | high |
| Kim 2021 | other psychological difficulties | Yes | Yes | Yes/nr | No | Yes | Yes | Yes | Yes | Yes | 8 | high |
| Kitzmann 2003 | other psychological difficulties | Yes | Yes | No | Yes/nr | No/nr | No/nr | Yes | Yes | No/nr | 5 | L/M |
| Lavi 2019 | other psychological difficulties | Yes | Yes | Yes/nr | Yes/nr | No/nr | Yes | Yes | Yes | No/nr | 5 | L/M |
| Liu 2023b | other psychological difficulties | Yes | Yes | Yes/nr | Yes/nr | Yes | Yes | Yes | Yes | Yes | 7 | high |
| Luke 2013 | other psychological difficulties | Yes | Yes | Yes/nr | Yes/nr | No/nr | No/nr | No/nr | No/nr | No/nr | 2 | L/M |
| MacMillan 1999 | other psychological difficulties | Yes | Yes | Yes/nr | Yes/nr | No/nr | No/nr | No/nr | Yes | No/nr | 3 | L/M |
| McIntosh 2019 | other psychological difficulties | Yes | Yes | Yes/nr | Yes/nr | No/nr | Yes | Yes | Yes | No/nr | 5 | L/M |
| McKay 2021 | other psychological difficulties | Yes | Yes | Yes/nr | Yes/nr | Yes | Yes | No/nr | No/nr | No/nr | 4 | L/M |
| Mitiku 2024 | other psychological difficulties | Yes | Yes | No | Yes/nr | Yes | No/nr | No/nr | Yes | Yes | 6 | high |
| Neumann 1996 | other psychological difficulties | Yes | Yes | Yes/nr | Yes/nr | No/nr | No/nr | No/nr | No/nr | No/nr | 2 | L/M |
| Noonan 2020 | other psychological difficulties | Yes | Yes | Yes/nr | No | Yes | No/nr | No/nr | Yes | Yes | 6 | high |
| Petruccelli 2019 | other psychological difficulties | Yes | Yes | Yes/nr | Yes/nr | Yes | Yes | Yes | Yes | Yes | 7 | high |
| Pilkington 2021 | other psychological difficulties | Yes | Yes | Yes/nr | Yes/nr | Yes | Yes | Yes | Yes | Yes | 7 | high |
| Pinquart 2019 | other psychological difficulties | Yes | Yes | No | Yes/nr | Yes | No/nr | No/nr | Yes | Yes | 6 | high |
| Pinquart 2021 | other psychological difficulties | Yes | No | Yes/nr | Yes/nr | Yes | No/nr | No/nr | Yes | Yes | 4 | L/M |
| Tetik 2021 | other psychological difficulties | Yes | Yes | No | No | Yes | Yes | Yes | Yes | Yes | 9 | high |
| Todorov 2023 | other psychological difficulties | Yes | Yes | Yes/nr | No | Yes | Yes | Yes | No/nr | No/nr | 6 | high |
| Vu 2016 | other psychological difficulties | Yes | Yes | Yes/nr | No | No/nr | Yes | Yes | Yes | No/nr | 6 | high |
| Wang 2022a | other psychological difficulties | Yes | Yes | No | No | Yes | Yes | No/nr | Yes | Yes | 8 | high |
| Wilson 2010 | other psychological difficulties | Yes | Yes | Yes/nr | No | No/nr | No/nr | No/nr | Yes | No/nr | 4 | L/M |
| Wolfe 2003 | other psychological difficulties | No | Yes | Yes/nr | Yes/nr | No/nr | No/nr | No/nr | Yes | No/nr | 2 | L/M |
| Xiao 2023 | other psychological difficulties | Yes | Yes | No | Yes/nr | Yes | Yes | Yes | Yes | Yes | 8 | high |
| Yang 2024 | other psychological difficulties | Yes | Yes | Yes/nr | No | Yes | No/nr | No/nr | Yes | Yes | 6 | high |
| Yeo 2024 | other psychological difficulties | Yes | Yes | Yes/nr | Yes/nr | Yes | Yes | Yes | Yes | Yes | 7 | high |
| Yu 2022 | other psychological difficulties | Yes | Yes | Yes/nr | Yes/nr | Yes | Yes | Yes | No/nr | Yes | 6 | high |
| Zhang 2022a | other psychological difficulties | Yes | Yes | Yes/nr | No | Yes | Yes | Yes | Yes | Yes | 8 | high |
| Zhang 2023a | other psychological difficulties | Yes | Yes | Yes/nr | No | Yes | Yes | Yes | Yes | Yes | 8 | high |
| Zhang 2023b | other psychological difficulties | Yes | Yes | Yes/nr | No | Yes | No/nr | No/nr | No/nr | No/nr | 4 | L/M |
| Zhang 2023c | other psychological difficulties | Yes | Yes | Yes/nr | No | Yes | Yes | No/nr | Yes | Yes | 7 | high |
| Zhu 2023b | other psychological difficulties | Yes | Yes | Yes/nr | No | Yes | No/nr | No/nr | No/nr | No/nr | 4 | L/M |

PB = publication bias; T/A = title and abstract; FT = Full text; Qual = quality. For more detailed instructions see Appendix S4: Quality rating form
